# Structural chemistry of layered lead halide perovskites containing single octahedral layers

**DOI:** 10.1107/S2052252521005418

**Published:** 2021-06-30

**Authors:** Jason A. McNulty, Philip Lightfoot

**Affiliations:** a School of Chemistry, University of St Andrews, St Andrews KY16 9ST, United Kingdom

**Keywords:** layered perovskites, symmetry mode analysis, hybrid materials

## Abstract

We present a comprehensive review of the structural chemistry of hybrid lead halides of stoichiometry *A*Pb*X*_4_, *A*_2_PbX_4_ or *A* *A*′Pb*X*_4_, where *A* and *A*′ are organic ammonium cations and *X* = Cl, Br or I. These compounds may be considered as layered perovskites, containing isolated, infinite layers of corner-sharing Pb*X*_4_ octahedra separated by the organic species. First, over 250 crystal structures were extracted from the CCDC and classified in terms of unit-cell metrics and crystal symmetry. Symmetry mode analysis was then used to identify the nature of key structural distortions of the [Pb*X*_4_]_∞_ layers. Two generic types of distortion are prevalent in this family: tilting of the octahedral units and shifts of the inorganic layers relative to each other. Although the octahedral tilting modes are well known in the crystallography of purely inorganic perovskites, the additional layer-shift modes are shown to enormously enrich the structural options available in layered hybrid perovskites. Some examples and trends are discussed in more detail in order to show how the nature of the interlayer organic species can influence the overall structural architecture; although the main aim of the paper is to encourage workers in the field to make use of the systematic crystallographic methods used here to further understand and rationalize their own compounds, and perhaps to be able to design-in particular structural features in future work.

## Abstract 2

A comprehensive review of hybrid lead halide perovskites based on [Pb*X*_4_]_∞_ layers is presented. We use a crystallographic approach based on symmetry mode analysis to systematize key types of distortions, particularly octahedral tilting and layer shifts, in this extensive and diverse family of materials.

## 1. Introduction   

Lead halide perovskites (LHPs) have recently revolutionized the field of solar cells, in addition to showing novel and promising properties in several other areas, such as luminescence, ferroelectricity *etc.* (Green *et al.*, 2014 ▸; Smith *et al.*, 2019 ▸; Zhang, Song, Cheng *et al.*, 2020 ▸). The diversity of chemical composition and structural architecture in this enormous and rapidly expanding family of materials not only creates great opportunities for the synthetic and structural solid-state chemist, but also makes the field somewhat overwhelming for the newcomer. For those of us with long memories, the excitement and opportunities available for the solid-state chemist are somewhat reminiscent of the explosion in work on layered cuprate perovskites in the late 80s and early 90s, at the peak of the high-*T*c superconductor revolution. Indeed, with the advent of ‘hybrid’ inorganic–organic systems in LHPs, the diversity of the field is clearly much greater than in more traditional inorganic-only systems. There have been many excellent reviews of the field of LHPs over the past few years (Saparov & Mitzi, 2016 ▸; Smith *et al.*, 2018 ▸; Mao *et al.*, 2019 ▸) which have focused on various aspects from the underlying chemistry and crystal structure to optimization of electronic and optical properties and further towards material processing and device manufacture. The purpose of the present review is to take a more crystallographically oriented view of the state-of-the-art in the area of ‘layered hybrid perovskites’, LHPs, specifically those containing a single ‘perovskite-like’ octahedral layer of stoichiometry [Pb*X*_4_]_∞_ (*X* = Cl, Br, I), in which the layers are separated by cationic organic moieties (*A*, *A*′) to give overall compositions *A*Pb*X*_4_, *A*_2_Pb*X*_4_ or *AA*′Pb*X*_4_. Even within this sub-field there are well over 250 crystal structures reported in the Cambridge Crystallographic Database (census date 11/11/20). Hence, we shall not refer to the copious body of work on 3D perovskite structures, such as (CH_3_NH_3_)PbI_3_ or the variety of ‘0D’ or ‘1D’ perovskite-related materials, based on chain-like structural fragments or isolated Pb*X*_6_ octahedra. Moreover, in order to keep the review of a manageable size and digestible to the less-expert reader, we also limit our analysis to so-called (001)-cut layered perovskites: hence neither the related (110) or (111)-cut families nor the (001)-cut families with double, triple or higher-order perovskite-like layer thicknesses will be covered here.

We first briefly introduce the various families of perovskites, before proceeding to describe the types of structural distortion that exist in layered perovskites, then using these as a means of classification of the currently known examples. We shall primarily focus on the detailed structural nature of the inorganic [Pb*X*_4_]_∞_ layers themselves, and then consider how this is influenced by the variety of *A*-site molecular cations which might determine the detailed architecture of these layers and their interactions: these features are ultimately the main driver influencing the physical properties of the resulting materials.

## 2. A brief introduction to perovskite crystallography   

### 2.1. What is a perovskite?   

**Figure 1:**
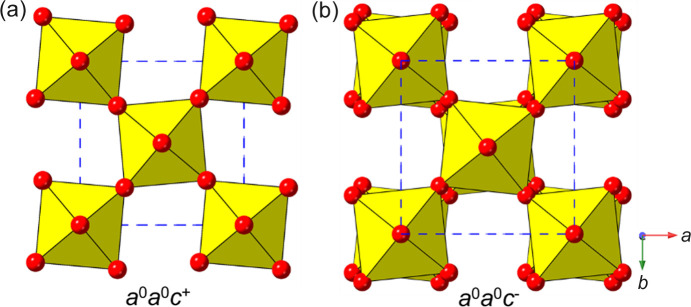
Octahedral framework of the ideal cubic perovskite showing the structure with (*a*) in-phase and (*b*) out-of-phase octahedral tilting, described by Glazer tilt notations *a* ^0^ *a* ^0^ *c* ^+^ and *a* ^0^ *a* ^0^ *c* ^−^, respectively. *B*-centred octahedra and *X* atoms are shown in yellow and red, respectively.

The name ‘perovskite’ originated in the discovery of the mineral Perovskite, CaTiO_3_, in 1839 (Raveau, 2007 ▸). This mineralogical curiosity later blossomed into arguably the most diverse and important class of compounds in solid-state chemistry. The generic composition of perovskites may be regarded as *ABX*_3_, where *A* and *B* are ‘large’ and ‘small’ cations, respectively, and *X* is an anion. The aristotype crystal structure has cubic symmetry, space group *Pm* 3 *m*, and consists of a cubic close-packed array of *A* and *X*, with *B* occupying 1/4 of the octahedral interstices, in an ordered manner [Fig. 1 ▸(*a*)]. Note that the word ‘cubic’ here is used in two different senses. A ‘cubic perovskite’ does not necessarily adopt a cubic crystal system, and symmetry-lowering is the norm, due to the well known tolerance factor and octahedral tilting effects; indeed Perovskite itself is orthorhombic! Moreover, there are many further variants on this basic compositional and crystal chemistry, and there is currently confusion and conflict in the literature regarding ‘what exactly is a perovskite?’ (Mercier, 2019 ▸; Breternitz & Schorr, 2018 ▸; Kieslich & Goodwin, 2017 ▸). This is unfortunate, but perhaps inevitable, in such a diverse field, and may require an international committee to propose some clear guidelines and definitions of nomenclature in this area. The use of the phrase ‘layered perovskite’ in the present work corresponds to the personal opinions and preferences of the authors and it is not intended to impose on other authors.

### 2.2. Octahedral tilting and symmetry mode analysis   

One ubiquitous type of distortion in cubic perovskites is ‘tilting’ of the octahedral *BX*_6_ units, which occurs due to a size mismatch between the *A* and *B* cations, governed by the Goldschmidt tolerance factor, *t*:

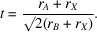

Glazer (1972 ▸) originally classified all the ‘simple’ tilts in cubic perovskites, and this was later updated by Howard and Stokes (1998 ▸) using group-theoretical analysis to give 15 possible simple tilt systems. The Glazer notation uses three lower case letters to specify the relative magnitudes of the tilts along the principal axes of the aristotype (‘parent’) unit cell. Superscripts ‘+’ or ‘−’ are used to specify whether these tilts occur ‘in-phase’ or ‘out-of-phase’ relative to each other, considering only a 2 × 2 × 2 array of corner-linked rigid octahedra. Thus, for example, Glazer tilt systems *a* ^0^ *a* ^0^ *c* ^+^ and *a* ^0^ *a* ^0^ *c* ^−^ are shown in Figs. 1 ▸(*a*) and 1(*b*).

These structural deviations from a high-symmetry parent structure can be regarded as ‘modes’ of distortion (like normal vibrational modes), which are amenable to the application of group-theoretical methods and representational analysis (Campbell *et al.*, 2006 ▸; Orobengoa *et al.*, 2009 ▸; Howard & Stokes, 1998 ▸). Indeed it is the application of these methods and, in particular the advent of user-friendly graphical software, such as *ISODISTORT* (Campbell *et al.*, 2006 ▸) and *AMPLIMODES* (Orobengoa *et al.*, 2009 ▸), amenable to non-expert users, that has allowed solid-state chemists to take a fresh look at structural phenomena of this type, in a much more rigorous and systematic way than was previously available. The *a* ^0^ *a* ^0^ *c* ^+^ and *a* ^0^ *a* ^0^ *c* ^−^ tilts in Glazer notation can be described with irreducible representations (irreps) with labels *M*_3_^+^ and *R*_4_^+^, respectively, using the notation by Miller & Love (1967 ▸). The distortions associated with these irreps correspond to the ‘freezing-out’ of phonon modes at specific points of the first Brillouin zone of the parent cell. This has two important consequences for solid-state chemists: (i) if a suitable ‘parent’ model for a particular structure type can be derived, experimentally or otherwise, then structural distortions in ‘real’ examples of this structure type can be easily and systematically understood in terms of these constituent irreps. (ii) Since the capital letter (*e.g.* M or R) in the irrep label corresponds to a particular point in reciprocal space, these distortions can quite easily be identified from a diffraction experiment, as they will give rise to particular types of supercell relative to the parent, higher-symmetry unit cell. Such ‘symmetry mode analysis’ is therefore an invaluable tool for the solid-state chemist in identifying common structural features across an otherwise apparently diverse range of (related) crystal structures (Campbell *et al.*, 2006 ▸; Orobengoa *et al.*, 2009 ▸; Talanov *et al.*, 2016 ▸; Boström *et al.*, 2018 ▸; Howard & Stokes, 1998 ▸). We shall see that, by using the standard ‘parent’ phases for either Ruddlesden–Popper (RP: *I*4/*mmm*) or Dion–Jacobson (DJ; *P*4/*mmm*) structures, we can easily identify tilt modes and other key types of distortion, unambiguously. Throughout this work we use the on-line tool *ISODISTORT* to perform this analysis (Campbell *et al.*, 2006 ▸). The symmetry labels for each type of distortion are dependent on the parent phase (and unit-cell origin choice) used, but they will be self-consistent for a given sub-family of LHPs. These will be introduced, as required.

### 2.3. Layered perovskites   

**Figure 2:**
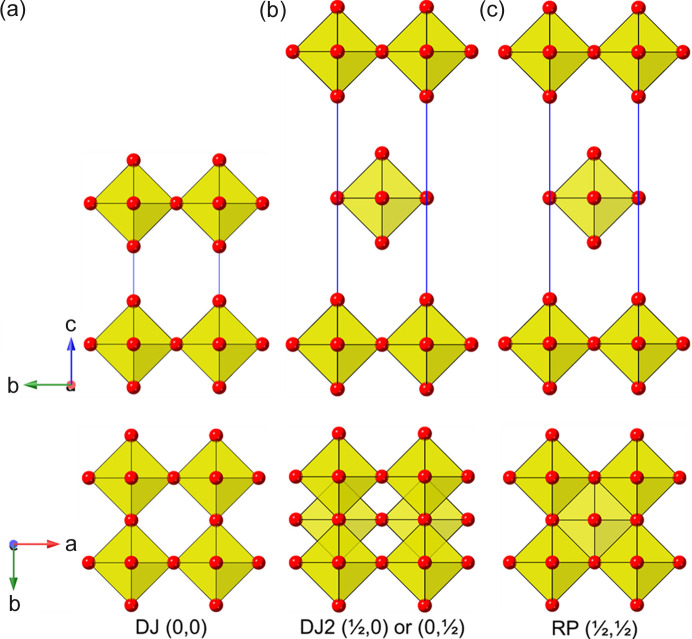
Undistorted structures of the simplest *n* = 1 layered perovskites viewed between layers (top) and along the layer stacking direction (bottom) highlighting the completely ‘eclipsed’, partially ‘staggered’ and completely ‘staggered’ adjacent layers of the (0, 0) and (1/2, 0) Dion–Jacobson (DJ and DJ2, respectively) and (1/2, 1/2) Ruddlesden–Popper (RP) phases.

Here, we shall use the term ‘layered perovskite’ to mean a compound with a crystal structure that can be easily derived from the cubic perovskite structure by ‘slicing’ through octahedral apices along a particular crystallographic direction, and inserting additional species between the resultant layers. There are several common types of layered perovskite, of which two are relevant in this work. RP and DJ phases were originally observed in mixed-metal oxides, (Ruddlesden & Popper, 1957 ▸; Dion *et al.*, 1981 ▸) and these were identified as having generic compositions *A*_2_ *A*′*_n_*_−1_ *B* *_n_* *X*_3*n*+1_ and *AA*′*_n_*_−1_ *B* *_n_* *X*_3*n*+1_, respectively. For the *n* = 1 case considered here, the aristotype compounds can be taken as the tetragonal systems K_2_NiF_4_ (space group *I*4/*mmm*; Balz & Plieth, 1955 ▸) and TlAlF_4_ (space group *P*4/*mmm*; Brosset, 1935 ▸), respectively. It should be noted that, in addition to the compositional differences stated above, a key structural distinction between the original two families is in the nature of the relative positioning of adjacent [*BX*_4_]_∞_ layers. Thus, we can see (Fig. 2 ▸) that the adjacent layers in the DJ family are ‘eclipsed’ relative to each other [a coordinate displacement of (0,0)], whereas those in the RP family are staggered by (1/2, 1/2). A further important variant of these two structure types is the intermediate case, with a staggering of (0, 1/2) or (1/2, 0): here the parent phase is orthorhombic, with space group *Ammm* [taking *c* as the ‘layer stacking’ direction; parent phase, NaWO_2_Cl_2_; Abrahams *et al.* (1991 ▸)].

Octahedral tilting is also a recognized and common feature in layered perovskites, (Benedek *et al.*, 2015 ▸; McCabe *et al.*, 2015 ▸) and we shall describe this in terms of both Glazer-like notation and using irrep labels for the relevant tilt modes. In the case of single-layer layered perovskites, the tolerance factor is clearly of no direct relevance, although the nature of the interaction of the interlayer species with the [*BX*_4_]_∞_ framework will certainly influence the nature of tilting. When we use the word ‘rotation’ rather than tilt this specifically applies to a mode acting around the axis perpendicular to the layer direction.

### 2.4. Hybrid layered perovskites   

With the advent of hybrid perovskites, inorganic solid-state chemistry had already paved the way for a useful description of the structural architectures of these compounds in terms of octahedral tilting and other distortions such as intra-octahedral distortion indices (Aleksandrov & Bartolomé, 2001 ▸; Woodward, 1997 ▸; Baur, 1974 ▸; Howard & Stokes, 1998 ▸). However, the inclusion of non-spherical, and often highly anisotropic, molecular species at the inter-layer *A* sites opens up a new level of complexity in hybrid layered perovskites. One particular feature of note is the much greater tendency for ‘slippage’, ‘shift’ or ‘staggering’ of adjacent perovskite blocks relative to each other, such that the conventional criteria used in recognizing RP versus DJ phases can no longer be applied simply. We will use ‘layer shift’ from now on to describe this. In fact, it has already been recognized that a range of degrees of shift of adjacent inorganic layers are observed in LHPs which span the RP–DJ regime (Tremblay *et al.*, 2019 ▸; Marchenko *et al.*, 2021 ▸). We therefore choose a definition of ‘RP’ and ‘DJ’ solely in terms of the degree of layer shift, rather than the original additional differences in *A*/*B* stoichiometry. In accord with Tremblay *et al.* (2019 ▸), we shall refer to any layered LHP, regardless of stoichiometry, as having an inter-layer offset close to (1/2, 1/2) as RP-like (‘near-RP’ or nRP), any having an offset near (0, 0) as DJ-like (nDJ) and any having an offset near (0, 1/2) as DJ2-like (nDJ2). Criteria similar to those of Tremblay will be used to define how close the structures are to one of the ideal types, by use of the layer-shift parameter Δ. We shall also see that layer shift can also be easily understood in terms of symmetry mode analysis, with specific modes occurring commonly, regardless of other simultaneous types of distortion.

The next sections describe a comprehensive survey and classification of all structurally well characterized (001)-cut LHPs of stoichiometry *A*PbBr_4_ or *A*_2_PbBr_4_. These structures are taken from the CCDC (Groom *et al.*, 2016 ▸) up to 11/11/20. In our initial survey, we began by classifying the unit-cell metrics of all the known structures in relation to either of the generic structure types RP, DJ or DJ2. It was immediately apparent that, although there is a wide diversity of variants spanning these ideal ‘end-members’, several common themes in types of distortion and types of supercell emerge. The space groups listed for each structure in Tables 1–5 are those of the complete structures (*i.e.* including the organic moieties), whereas the irreps reported are those of the space groups of the RP and DJ parent inorganic frameworks alone. We have derived all the tabulated structures from either the RP or DJ parent, *i.e.* K_2_NiF_4_-type (space group *I*4/*mmm*) or TlAlF_4_-type (space group *P*4/*mmm*), respectively. It is important to appreciate that irreps belonging to different space groups are actually distinct, even if they have the same semi-arbitrary label. Though the RP and DJ parent space groups share some common irrep labels, it should be clear to the reader which parent space group an irrep belongs to from the context of the relevant section. The space group need not be specified for each instance of an irrep label.

## 3. A classification of (001)-cut LHPs of stoichiometry *A*PbBr_4_ or *A*_2_PbBr_4_   

We find it convenient to classify this vast array of structures in terms of the observed unit-cell metrics, and their relationship to the parent RP or DJ. In particular we shall use the number of octahedral layers per unit cell repeat as a key discriminator. In other words, regardless of whether the resultant layer shift looks ‘RP-like’, ‘DJ-like’ or ‘DJ2-like’, we shall aim to derive all the structure types from either an RP parent (*I*4/*mmm*), for those with two or more layers per unit cell, or a DJ parent (*P*4/*mmm*) for those with one layer per unit cell. Structures that appear ‘DJ2-like’ will be derived from the RP parent. In many cases, it is equally feasible to use the alternate parent phase, leading to an equivalent result. For the case of LHPs, the parent tetragonal unit-cell metrics are *a*_RP_ ≃ *a*_DJ_ ≃ 5.5 – 6.5 Å, for chlorides to iodides, with *c*_RP_, *c*_DJ_ obviously being variables, dependent on the nature of the organic moieties.

In all the Tables, we refer to the individual structures using CCDC deposition numbers. We start with the two-layer cases as these more usefully illustrate some of the structural principles observed. The parent phase is the K_2_NiF_4_ type, *i.e.* with two adjacent, fully staggered layers forming the unit cell repeat perpendicular to the layer direction. Throughout the following survey it is worth noting that the ‘layered perovskite’ convention of taking the layer-stacking direction as the *c* axis does not always correspond to the conventional setting of the resultant space group; authors differ on which convention they follow, so occasionally different space group settings appear in the data presented herein. In Section 3.1 we describe and classify structures with two octahedral layers and include relatively simple structures with unit-cell volumes up to 2*a*_RP_ × 2*a*_RP_ × *c*_RP_, *i.e.* eight formula units per unit cell. In Section 3.2 we describe structures with one octahedral layer per unit cell, and in Section 3.3 we describe more complex structures with at least one axial metric more than double the parent phase.

### 3.1. Structures with two octahedral layers per unit cell (derived from RP parent)   

#### 3.1.1. Unit-cell metrics equivalent to the parent phase (∼*a*_RP_ × *a*_RP_ × *c*_RP_)   

**Figure 3:**
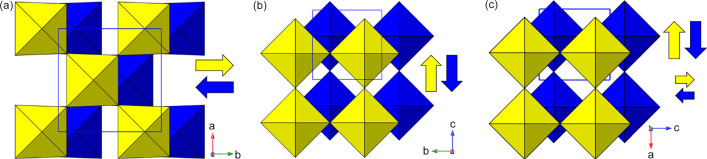
Three distinct M_5_^−^ displacive modes, derived from the RP parent. (*a*) M_5_^−^(a, 0) antiparallel displacements of adjacent layers along one in-plane axis of the supercell; (*b*) M_5_^−^(a, a) showing anti-parallel displacements of adjacent layers along one in-plane axis of the parent cell; (*c*) M_5_^−^(a,b) allows two different amplitudes of displacement along the in-plane axes of the parent cell. It can be seen that the (a, 0) mode results in layers shifted towards DJ type, (a, a) towards DJ2 type, and (a, b) provides both degrees of freedom. Note that each mode also allows a distortion of the octahedra, which is illustrated in (*a*) by the differing lengths of the *trans*-octahedral edges.

We first note that Balachandran *et al.* (2014 ▸) conducted a survey of known inorganic oxides adopting the *A*_2_ *BX*_4_ RP structure, and found the high-symmetry aristotype phase in space group *I*4/*mmm* to be the most common variant. In stark contrast, the crystal structures of only three LHPs have been reported in the aristotype space group *I*4/*mmm* (Table 1 ▸). These are structures of ‘high-temperature’ phases, exhibiting disordered organic moieties, and all subsequently undergo phase transitions to ordered polymorphs with lower symmetry supercells on cooling. In addition, there are two simple derivatives which retain the body-centred symmetry. There are three further examples of lower symmetry structures with these cell metrics in our survey. Note that it is impossible for such cell metrics to accommodate ordered octahedral tilting distortions; these require expanded supercells of at least twice the volume of the parent phase. Therefore, the driver for these lower symmetry yet ‘parent cell size’ structures turns out to be essentially a layer-shift mode. This mode is designated M_5_^−^; in fact, it has more flexibility than a ‘rigid mode’ layer shift, also allowing some intra-octahedral distortions. More specifically, there are often several distinct options for such a mode (and likewise for the octahedral tilt modes). In this case we find one example (1937296) of M_5_^−^(a, a) symmetry and one (1211182) of lower M_5_^−^(a, b) symmetry. The additional notation (a, a) *etc*. defines the so-called ‘order parameter direction’ (OPD) (Campbell *et al.*, 2006 ▸); for further details see the supporting information. Examples of these modes are shown schematically in Fig. 3 ▸, where the mode amplitudes are chosen to keep the octahedra close to regular. It can be seen that each mode, acting individually, causes a specific lowering of the symmetry from the parent symmetry; for example, the M_5_^−^(a,a) mode naturally results in a space group *Pmmn* with approximate cell metrics of the parent phase. In the case of 1937296 the layers are shifted along the *c* axis and the magnitude of this mode results in a structure close to (0, 1/2) staggering, *i.e.* nDJ2. The second example, 1211182, is a lower symmetry (polar) version of this, also of nDJ2 type. A third case (1186561) was a very early example of an LHP, and was refined with disordered octahedra: the authors stated that there was a possible ‘unresolved superlattice structure’, and we agree that this structure probably does contain octahedral tilting modes that were not identified, so does not formally belong in this section; in fact, a subsequent re-determination is included later (200737 in Section 3.2).

Note there is one further possible symmetry for the M_5_^−^ mode: M_5_^−^(a, 0), which we shall see in the next section. The relative directions of the shifts should be clear from Fig. 3 ▸. Although the OPDs are fixed relative to the crystallographic axes, it should be noted that there is no direct correspondence between the two components of the order parameter and the crystallographic directions themselves.

#### Metrics 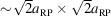 in the layer plane   

3.1.2.

These structures are detailed in Table 2 ▸. A very common distortion of a cubic perovskite unit cell (unit-cell parameter *a*_p_) is an approximate 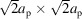 supercell caused by octahedral tilting. Such effects are seen, for example, in both of the Glazer systems in Fig. 2 ▸. It comes as no surprise that such features are also commonplace in layered perovskites. We note that the unit-cell volume is effectively doubled in all these derivatives, but the *c* axis remains equivalent to that of the parent phase (*i.e.* not doubled relative to the RP parent, but still encompassing two adjacent octahedral layers per *c* axis repeat). In addition, the body-centring is lost. It can be seen that there is a diversity of resultant space groups. As discussed above, we are now anticipating that structures may contain two particular types of distortion of the [Pb*X*_4_]_∞_ layers (*i.e.* octahedral tilting and layer shift). Our classification therefore considers those structures with layer shifts and octahedral tilting, either independently or cooperatively, starting from the simplest to the more complex.

*Structures with no octahedral tilting, but with layer shifts.* We include in Table 2 ▸, and subsequent tables, the parameter Δ which describes the extent of layer shift between neighbouring layers (highlighted in Fig. 3 ▸). In fact, in a general case this is a 2D parameter (Δ_1_, Δ_2_). We have included these parameters only for selected series of relatively simple structure types, using manually calculated Δ values, based on the relative displacements of Pb atoms only, in neighbouring layers. For more complex structures we have chosen to state a visually estimated layer shift outcome (*i.e.* nRP, nDJ or nDJ2). A Δ value of (0.25, 0.25) signifies the crossover between ‘nDJ’ (for Δ < 0.25) and ‘nRP’ (for Δ > 0.25). nDJ2 structures have Δ values closer to (1/2, 0), *i.e.* Δ_1_ > 0.25, Δ_2_ < 0.25.

**Figure 4:**
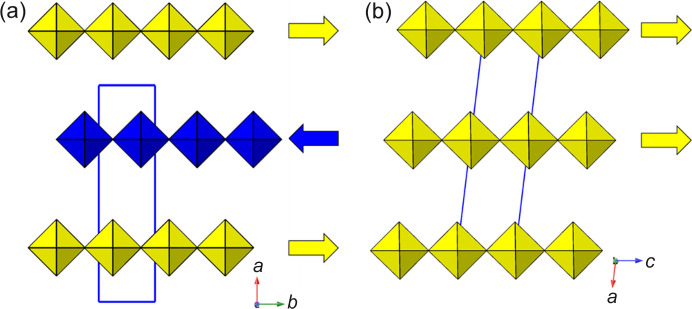
Comparative effects of the two generic types of layer-shift mode acting on the RP parent (*a*) M_5_^−^(a, a) mode (see also Fig. 3 ▸) and (*b*) the analogous Γ_5_^+^ mode. Note that the former leads to orthorhombic symmetry, whereas the latter corresponds to a monoclinic distortion.

There are two distinct types of layer shift which may be present in layered perovskites. These can be described and classified conveniently in the language of symmetry mode analysis. The first type is represented by a ‘gamma mode’ (*i.e.* acts at the Brillouin zone centre), usually designated Γ_5_^+^. It acts simply to slide adjacent layers in the same sense relative to each other along one crystallographic axis, and results in a lowering of symmetry to monoclinic (Fig. 4 ▸). The resultant Δ values for this type of distortion can be simply calculated from the unit-cell metrics (see, for example, the equations in Section 3.2.2), although direct graphical measurement [for example using *Crystalmaker* (Palmer, 2014 ▸)] also gives a good approximation. It will be shown that this type of monoclinic distortion is a common feature in LHPs, leading to bridging of the RP to DJ regimes.

The second type of layer-shift mode is the antiferrodistortive M_5_^−^ type introduced in Section 3.1.1, *i.e.* a shift of adjacent octahedral layers in *opposite* directions relative to an axis perpendicular to the layer direction (Figs. 3 ▸ and 4 ▸). The mode, acting alone, leads to a lowering of symmetry from tetragonal to orthorhombic, and so the corresponding Δ values are straigtforward to calculate, from the associated difference in *x*, *y* or *z* coordinates. The first and unique example here is 1852626, which occurs in space group *Cmcm*. This example is a high-temperature (413 K) polymorph of a phase that appears at ambient temperature in the space group *Pbcn* (1845548). It has no octahedral tilting, just the M_5_^−^(a, 0) displacive mode (Fig. 3 ▸), which is distinct from those seen in Section 3.1.1. As far as we are aware, there are no previously reported examples of any M_5_^−^ type of distortion in inorganic *A*_2_ *BX*_4_ structures. Two further examples also exhibit layer shifts without octahedral tilting: these examples (641642 and 167103) occur in space group *C*2/*c* and again feature no octahedral tilting; however, they have the antferrodistortive shift mode M_5_^−^, which describes a displacement along the *b* axis (as occurs in the first example) and, in addition, a purely displacive Γ_5_^+^ mode, which leads to an additional displacement along the *a* axis, and a monoclinic distortion.

**Figure 5:**
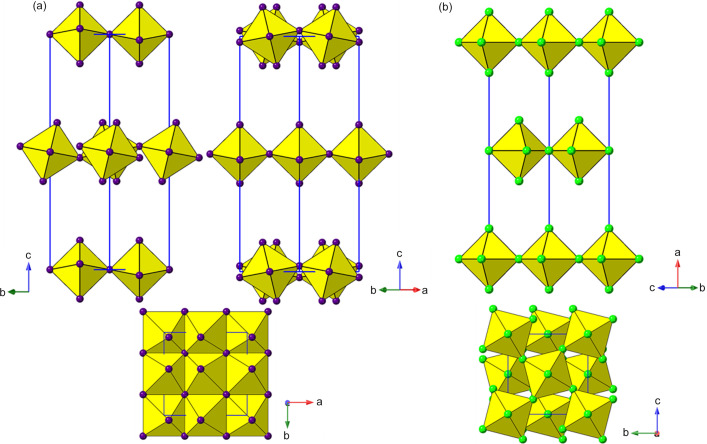
Octahedral frameworks of two structures with a single tilt mode (*a*) X_3_^+^(a, a) tilt mode (1863837) and (*b*) X_2_^+^(0, a) rotation mode (2016195). Note the out-of-phase tilting around the *ab* plane and alternating direction of tilts in (*a*) and the absence of layer-plane tilting but presence of rotations of the same degree around the *c* axis in (*b*).

*Structures with a single type of octahedral tilt and no layer shift.* The structure 1863837, in space group *P*4_2_/*ncm*, is a unique example of one of the simplest types of distortion in this family; Balachandran *et al.* (2014 ▸) reported five examples of oxides with this structure type. The structure exhibits an out-of-phase tilting of octahedra around the *ab* plane, but the direction of this tilt alternates in adjacent layers [Fig. 5 ▸(*a*)] hence retaining the tetragonal symmetry. The corresponding tilt mode is designated X_3_^+^(a, a) (see the supporting information for further details of some of these tilt mode descriptions). It is necessary to use an extended Glazer-like notation to describe the tilts in these systems that contain two adjacent octahedral layers which, while they may be symmetry-related, may also contain opposite directions, or signs, of the corresponding tilts. In the adapted Glazer-like notation, *e.g.* as used by Hayward (Zhang *et al.*, 2016 ▸), the tilt system here is *a* ^−^ *b* ^0^ *c* ^0^/*b* ^0^ *a* ^−^ *c* ^0^. Aleksandrov & Bartolomé (2001 ▸) undertook a comprehensive group-theoretical analysis of tilting in RP phases, using a different, but equivalent, notation and designated this tilt system ϕ00/0ϕ^−^0; we shall use the Glazer-like notation. A further two examples (1934896 and 1992692) are also based on a single X_3_^+^ tilt mode, but there are two key distinctions from the previous structure: first, the X_3_^+^ mode has a different OPD, and is designated X_3_^+^(0, a): the octahedral tilt system is *a* ^−^ *a* ^−^ *c* ^0^/−(*a* ^−^ *a* ^−^)*c* ^0^. This structure type is the most common tilted type reported amongst the inorganic oxide analogues by Balachandran *et al.* (2014 ▸). Second, there is an additional, purely displacive (Γ_5_^−^) mode which leads to a polar space group, *Aba*2 or *Cmc*2_1_, the former compound has been demonstrated to exhibit ferroelectricity (Zhang, Song, Chen *et al.*, 2020 ▸).

There are nine examples in Table 2 ▸ (commencing 2016195) of phases exhibiting a single X_2_^+^(0, a) rotation mode and no other significant mode. This results in unit-cell metrics 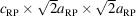 and space group *Cmca* (alternatively 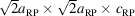 and non-standard space group *Acam*). Note that this, by coincidence only, is the same space group as for the examples discussed, with active mode X_3_^+^(0, a). The tilt system here can be designated *a* ^0^ *a* ^0^ *c*/*a* ^0^ *a* ^0^ *c*, with no tilting relative to the layer-plane but rotations around the axis perpendicular to the layers, with each layer having the same degree of rotation [Fig. 5 ▸(*b*)]. Note that we do not use the superscript notation for the *c* axis in the case of layered perovskites with single octahedral layers (as Glazer’s original concept explicitly relies on octahedra being linked in the third direction). We use ‘*c*’ to mean ‘rotated perpendicular to the layer direction’ and *c* ^0^ to mean ‘no rotation’ (Li, Clulow *et al.*, 2019 ▸). The symbol (−*c*) is used if the second layer is rotated contrary to the first (which is only really relevant if there is a partial layer shift from the ideal RP parent).

The next subset of structures (commencing 2003637) involves the same single rotation mode [X_2_^+^(0, a)] but the symmetry is lowered to *Cmc*2_1_. These ten examples are simple derivatives of the corresponding *Cmca* subset above, but they have an additional displacive mode (Γ_5_^−^) acting along the *c* axis, which leads to the polar space group. Nevertheless, they have Δ = (0.5, 0.5) and can be regarded as RP. There is often a considerable distortion of the Pb*X*_6_ octahedra present in these structures which leads to the observation that the polar axis (*c*) is significantly shorter than the other in-plane axis (*b*) in each case.

*Structures with a single type of octahedral tilt and layer shift.* There are several further examples of structures (237190–1975109) containing a single tilt mode, X_3_^+^(0, a); however, this is now supplemented by a further key mode, designated M_5_^−^ (0, −b), which describes a shift of adjacent octahedral layers in *opposite* directions along the *b* axis (Fig. 3 ▸). This type of superposition of two modes may be written as X_3_^+^ ⊕ M_5_^−^. The resulting space group is orthorhombic, *Pbcn*. Again, such an ‘antiferrodistortive’ displacement means that the octahedral layers are no longer in perfectly staggered configuration relative to each other. In other words, such structures to some extent fall between the extremes of ‘ideal RP’ and ‘ideal DJ’ types. In these cases, where the unit cell contains two adjacent layers per unit cell repeat, we choose to consider them to be derived from the RP rather than the DJ parent structure but, of course, the degree of layer shift will dictate whether these examples might be regarded as nDJ or nRP in the classification introduced by Tremblay *et al.* (2019 ▸). The parameter Δ therefore comes into play here (defined in these orthorhombic cases as simply the difference in *y* parameters between two Pb atoms in neighbouring layers, shown in Fig. 3 ▸). As can be seen, the majority of the examples here can be described as nDJ. Note that it is convenient in simple supercells with ‘ 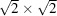 ’ in-plane metrics to determine the Δ parameters relative to the supercell axes, but Δ_1_ = Δ_2_ in these cases.

There are also several further, lower symmetry structures that are derived from a single rotation described by the X_2_^+^ mode, but with additional modes leading to lower symmetry space groups. This set consists of eight examples (1938882 onwards), which adopt the orthorhombic space group *Pnma*, with metrics 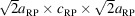 . The X_2_^+^(0, a) is a key mode, which could be described as tilt system *a* ^0^ *a* ^0^ *c*/*a* ^0^ *a* ^0^ *c*, in the case of a perfect RP structure. However, as in the case of the *Pbcn* structure types above, this is now supplemented by the further key mode, M_5_^−^ (0, b−), which describes a shift of adjacent octahedral layers in *opposite* directions along the *a* axis (Fig. 3 ▸). This again means such structures to some extent fall between the extremes of ‘ideal RP’ and ‘ideal DJ’ types. The parameter Δ signifies that each of the examples here are nRP. However, for significantly shifted layers, the choice of *c* or (−*c*) symbols is open to definition, and may be taken from the relative rotations of the ‘nearest’ octahedron in the adjacent layer. For the nDJ structures these should perhaps be described as *a* ^0^ *a* ^0^ *c*/*a* ^0^ *a* ^0^(−*c*). It should also be noted that the distortive effect of the M_5_^−^ mode is often more dominant than the octahedral rotation mode (perhaps a manifestation of the stereochemically active Pb^2+^ lone pair); an example is 1938883. It can be seen, in even in these relatively ‘simple’ examples of LHPs, that the unambiguous assignment of Glazer-like tilt systems is not as straightforward as it is in the traditional inorganic layered perovskite families. There is a further example (1914148) of a combination of X_2_^+^(0, a) rotation with a different shift mode, M_5_^−^ (b, 0), which naturally leads to space group *Pbcm*; in this case the rotational mode is again near zero.

*Structures with two types of octahedral tilt and no layer shift.* We now consider structures within this family of unit-cell metrics which accommodate two distinct types of tilt mode. This subset is very common, with 24 examples (commencing 1938881). It has contributions from the two modes we have seen individually: X_2_^+^(0, a) and X_3_^+^(b, 0), and results in the unit-cell metrics 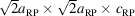 and space group *Pbca*. The tilt system can be regarded as *a* ^−^ *a* ^−^ *c*/−(*a* ^−^ *a* ^−^)*c*. It is perhaps not surprising that this tilt system is common, as it resembles the most common tilt system in 3D oxide perovskites, *a* ^−^ *a* ^−^ *c* ^+^ (or the GdFeO_3_ type).

**Figure 6:**
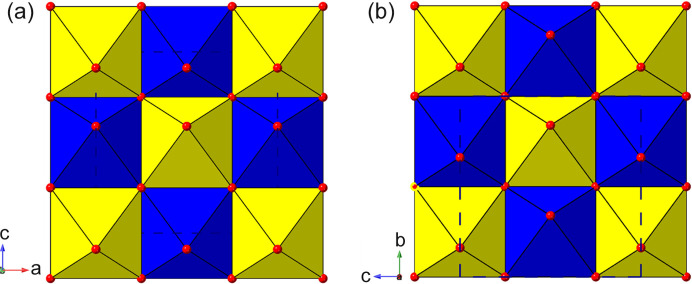
Comparison of the (*a*) X_3_^+^(0, a) and (*b*) X_4_^+^(0, a) modes, derived from the RP parent. Two layers are plotted, separated by half a unit cell. Notice that the top (yellow) layer tilt pattern is identical for each, but in the bottom (blue) layer tilts change in relative sense. The corresponding Glazer-like notation is *a* ^−^ *a* ^−^ *c* ^0^/−(*a* ^−^ *a* ^−^)*c* ^0^ and *a* ^−^ *a* ^−^ *c* ^0^/*a* ^−^ *a* ^−^ *c* ^0^, respectively.

*Structures with two types of octahedral tilt and layer shift.* Finally, we describe several classes of structure having, simultaneously, two tilts and one or two layer-shift modes. The first subset has the metrics 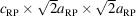 and space group either *C*2/*c* (centrosymmetric) or its polar derivative *Cc*. There are nine examples (1826587–956552). These structures have monoclinic, rather than orthorhombic, unit cells which means they have the additional degree of freedom, described by the Γ_5_^+^ strain mode, whereby the adjacent layers are permitted to slide ‘in-phase’ relative to each other leading to shifts intermediate between RP and DJ. The two tilt modes in this case are designated X_2_^+^(0, a) and X_4_^+^(0, a). This leads to the tilt system *a* ^−^ *a* ^−^ *c*/*a* ^−^ *a* ^−^ *c* for unshifted layers; however, the same issue of how to describe this taking into account nRP versus nDJ arises. Due to the additional complexity here, we will use the ideal ‘unshifted’ tilt system. At first sight, this may resemble the *a* ^−^ *a* ^−^ *c*/−(*a* ^−^ *a* ^−^)*c* system above. However, looking closely at the relationship between directions of tilts in neighbouring layers (Fig. 6 ▸), the distinction between the X_4_^+^ and X_3_^+^ tilt modes is clear.

The remaining structures in Table 2 ▸ fall into several types, which are mostly more complex variants, with different tilt systems, and different degrees of layer shift. We first describe those that are RP-like or nRP. The next two structures (1856671 and 1934873) are simply polar derivatives of the common *Pbca* type, above, with the tilt system *a* ^−^ *a* ^−^ *c*/−(*a* ^−^ *a* ^−^)*c*. The following structure (1903531) is again a derivative of the *Pbca* type: the lower symmetry is created, formally, by additional degrees of freedom in both layer shift and tilting [the modes are X_2_^+^(a, b) and X_3_^+^(c, 0), leading to the tilt system *a* ^−^ *a* ^−^ *b*/−(*a* ^−^ *a* ^−^)*c*]. However, mode decomposition shows that the true symmetry, at least as far as the inorganic network is concerned, is very close to *Pbca*. The next two structures (1119707 and 607740) can be regarded as lower symmetry variants of either the *Pnma* or *Pbca* structures above, having simultaneous X_2_^+^, X_3_^+^ and M_5_^−^ modes; *i.e.* a formal tilt system *a* ^−^ *a* ^−^ *c*/−(*a* ^−^ *a* ^−^)*c* together with a layer-shift mode. This places the first example close to RP type and the second closer to DJ. The remaining structures are closer to either DJ or DJ2 types. Taking the nDJ types first, three unusual examples are 1942543, 2016669 and 659021. These have a combination of X_2_^+^ and X_4_^+^ rotation/tilt together with the M_5_^−^ and Γ_5_^+^ shift modes.

The final examples in this section are most closely related to the DJ2 type, *i.e.* close to a neighbouring layer offset of (1/2, 0). The structures (1305732–1521055) in space group *P*2_1_/*c* exhibit simultaneous X_2_^+^ and X_3_^+^ tilt modes and the antiferrodistortive shift mode M_5_^−^ which again leads to a displacement along the *b* axis. However, in contrast to the two *P*2_1_2_1_2_1_ examples above, in this case there is also a simultaneous monoclinic distortion (Γ_5_^+^ mode) which describes the additional offset of adjacent layers along the *a* axis, resulting in DJ2 rather than DJ-like behaviour.

The final cases (1525376–1934876) have either tilt modes X_2_^+^ ⊕ X_4_^+^ or X_3_^+^ ⊕ X_4_^+^, each with additional M_5_^−^ and Γ_5_^+^ modes.

#### 3.1.3. Metrics ∼2*a*_RP_ × *a*_RP_ or 2*a*_RP_ × 2*a*_RP_ in the layer plane   

There are several structures with two octahedral layers per unit cell which also have one doubled cell axis in the layer plane, and some which have both axes doubled. These are presented in Table 3 ▸. We shall save the larger supercell structures for Section 3.3.

**Figure 7:**
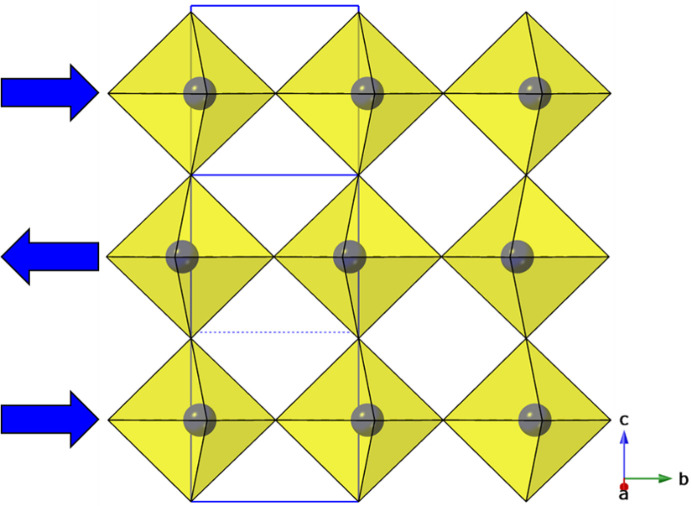
Antiferrodistortive displacement of Pb ions causes doubling of the *c* axis in nRP type [C_5_H_12_N]_2_PbCl_4_ (1962913).

*Structures with no octahedral tilting, but with layer shifts.* Five structures (1962913–1846391), all iodides, are reported in space group *C*2/*c* with metrics *c*_RP_ × *a*_RP_ × 2*a*_RP_. They display no octahedral tilting but have Γ_5_^+^ layer shifts: this degree of freedom leads to varied structure types between the RP and DJ2 types. Unit cell doubling along the *c* axis arises from an antiferrodistortive displacement of the Pb atoms along the *b* axis (Fig. 7 ▸), which is described by N_1_^−^ being the most significant mode. The mode is somewhat reminiscent of the Pb atom shifts in antiferroelectric PbZriO_3_ (Fujishita & Katano, 2000 ▸), and it is unique among the LHPs we have discussed so far. It should be noted that some of these examples show disorder within the inorganic framework. The next five structures (1938883–641643) have the cell metrics 2*a*_RP_× *c*_RP_ × *a*_RP_ and adopt the space group *Pnma* (or alternative setting *Pbnm*, *a*_RP_ × 2*a*_RP_ × *c*_RP_). These incorporate the M_5_^−^ displacement mode. In structures with these cells metrics, the M_5_^−^ mode acts along the doubled axis, but simultaneously there is also a Σ_4_ mode [full irrep label in this case is (a, a|0, 0; b, −b)] which, rather than being a tilt mode, acts to undulate the layers slightly out of plane. The structures vary between nRP and nDJ2 types.

*Structures with a single type of octahedral tilt and no layer shift.* Two quite high symmetry derivatives (1588974 and 1552603, space group *Imma*) have the cell metrics *a*_RP_ × 2*a*_RP_ × *c*_RP_. These are of particular interest as they are rare examples of *AA*′Pb*X*_4_ stoichiometries (*i.e.* having two distinct, ordered interlayer cations). These structures cannot be derived directly from the RP parent phase, so we use the (0, 1/2)-shifted parent in space group *Ammm* (Abrahams *et al.*, 1991 ▸). Indeed, they are perfect examples of DJ2 type, but they also have a single octahedral tilt mode. From the *Ammm* parent, the tilt mode is designated T_3_^+^, and the corresponding tilt system is *a* ^+^ *b* ^0^ *c* ^0^/−(*a* ^+^)*b* ^0^ *c* ^0^, although we note that 1552603 was modelled with disorder of the Br ligands. It should also be noted that there is no possibility of an *a* ^+^ tilt mode in an RP-derived structure (*i.e.* there is no suitable irrep of the space group *I*4/*mmm*).

**Figure 8:**
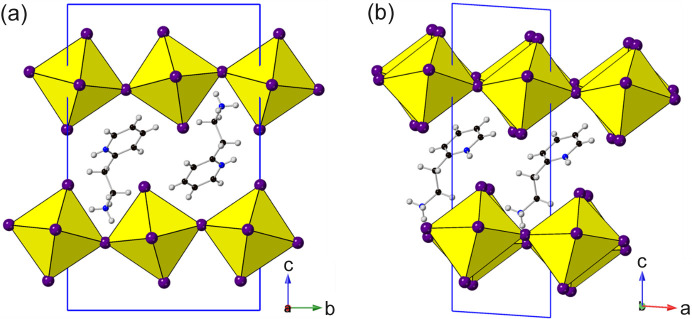
[C_7_H_12_N_2_]PbI_4_ (1944786) showing (*a*) *a* ^+^/*a* ^+^ tilts around *a* and (*b*) octahedral distortion (not a tilt) relative to *b*.

*Structures with more complex tilts and layer shifts.* Five further structures (1915486–659016) in the space group *P*2_1_/*n* with the metrics *a*_RP_ × 2*a*_RP_ × *c*_RP_ display a more complex set of distortions (starting from the RP parent phase these are designated Σ_3_, which is essentially a tilt, and Σ_4_, an octahedral distortion) and there is additional symmetry lowering due to two different types of layer shift: M_5_^−^ which acts along the *b* axis and Γ_5_^+^ (monoclinic distortion) which acts along *a*. The resulting tilts and displacements are shown in Fig. 8 ▸; although the symmetry is too low to define a rigorous tilt system, it is reminiscent of the *a* ^+^ *b* ^0^ *c* ^0^/−(*a* ^+^)*b* ^0^ *c* ^0^ type above. A further example (1841680) in *P*2_1_/*c* (*a*_RP_ × *c*_RP_ × 2*a*_RP_) has a similar resultant structure.

**Figure 9:**
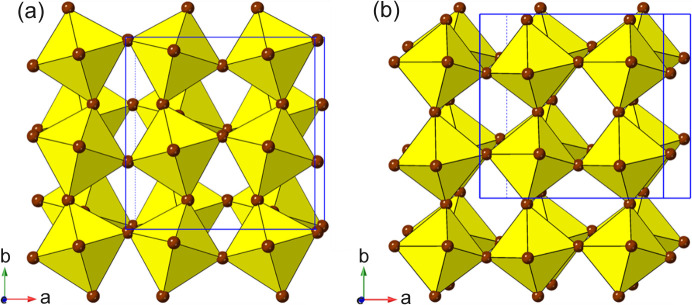
Comparison of two structures having the same unit-cell metrics (2*a*_RP_ × 2*a*_RP_ × *c*_RP_) and space group *C*2/*c*, but exhibiting significantly different resultant tilt and shift behaviour (*a*) [C_4_H_14_S_2_N_2_]PbBr_4_ (628793) and (*b*) [C_2_H_4_N_3_]_2_PbBr_4_ (1985833).

Finally, for this section, there are a few interesting and complex examples where both in-plane axes are doubled. The simplest of these is 628793, which has the metrics 2*a*_RP_ × 2*a*_RP_ × *c*_RP_, space group *C*2/*c*. A projection of the structure down the *c* axis is shown in Fig. 9 ▸(*a*). This combines two key modes: a rotation of octahedra around the *c* axis, designated P_4_ (a, −a), together with the now familiar M_5_^−^(a, a) shift mode, leading to an overall description P_4_ (a, −a|b, b). These can be regarded as the primary-order parameters, and acting together lead to the observed space group *C*2/*c* via the transformation matrix [(2,0,0) (0−2,0) (−1,0,−1)]. The P_4_ mode is new to us, but it is relatively common in purely inorganic RP phases (see Balachandran *et al.*, 2014 ▸). Acting alone, this mode would produce a unit cell size 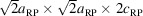 , whilst retaining a body-centred tetragonal symmetry and space group, *I*4_1_/*acd*. However, coupled with the M_5_^−^ mode [which in itself produces no expansion of unit-cell metrics, and space group *Pmmn*, seen in 1937296 (Section 3.1.1)] the unit-cell metrics become 2*a*_RP_ × 2*a*_RP_ × *c*_RP_. The resulting structure can be regarded as nDJ2, with a Glazer-like system approximately *a* ^0^ *a* ^0^ *c*/*a* ^0^ *a* ^0^(−*c*). Note that there is also a minor component of a more complex out-of-plane tilt mode [P_5_(0, 0; a, a)] allowed in these structures, in addition to the Γ_5_^+^ (monoclinic distortion) mode. This makes the pragmatic assignment of a Glazer-like tilt system difficult. For example, the following structure (1985833) has the same structure type but is nDJ, and contains a more significant P_5_ tilt mode [Fig. 9 ▸(*b*)]. In general, the most useful way to assign a tilt system here will depend on the relative significance of the P_4_ and P_5_ modes and the degree of layer shift. The next four examples in Table 3 ▸ (1043214–184082) are polar variants (*Cc*) of essentially the same mode combination. The resultant structure type can vary between nDJ, nDJ2 and nRP, depending on the relative degrees of the M_5_^−^ and Γ_5_^+^ displacements. For example, 2016668 is nRP and the P_5_ tilt mode is much more significant than the P_4_ rotation. The next example (1542463) in polar space group *Pn* appears to be a lower symmetry variant of the above, with an additional minor contribution from an antiferrodistortive Pb atom shift perpendicular to the layers, of symmetry N_2_^−^ (compare to 1962913).

The final three structures (1552604–1841683) in this section have the metrics 2*a*_RP_ × *c*_RP_ × 2*a*_RP_. These larger, low-symmetry unit cells have a diversity of allowed distortion modes, but often it is reasonable to pick out the most significant ones. The highest-symmetry example (1552604) has the space group *Pnnm*. This has the M_5_^−^ displacive mode leading to a nDJ2 structure, and tilt modes leading to the system *a* ^+^ *b* ^0^ *c*/−(*a* ^+^ *b* ^0^ *c*). The other two structures have four and two unique Pb sites, respectively. The former is close to RP type and has only a rotation mode around the *b* axis, but there are significant, and differing, distortions of each of the Pb sites. The latter is borderline, RP–DJ with *a* ^+^-like tilts, but again, other distortion modes are significant.

### 3.2. Structures with one octahedral layer per unit cell (derived from DJ parent)   

We chose to discuss structures with two octahedral layers per unit cell rather than a single layer first, not because they are ‘simpler’ but because they offer a much greater diversity of constituent distortions modes, from single tilt or displacement types to types with much greater degrees of freedom. In fact, the single-layer sub-family, discussed in this section, has far fewer degrees of freedom, but nevertheless has surprisingly few examples of ‘high symmetry’ structures (only one centrosymmetric and orthorhombic, for example). In contrast, despite the structural diversity described in Section 3.1, it can be noted that there is only a single example of a triclinic structure there. In this section we shall see that the vast majority of examples have monoclinic symmetry, and several derivatives of these have triclinic symmetry. In fact, 87 out of 108 structures in this section correspond to the same basic structure type! We shall derive these single-layer structures (Table 4 ▸) from the DJ-type parent (space group *P*4/*mmm*). As we shall see, the common features highlighted in Section 3.1, *viz*. octahedral tilting and layer-shift modes, also occur here, but the mode labels used to describe them are necessarily different (*i.e.* different parent Brillouin zone). It is therefore helpful to point out the different labels used to describe the corresponding tilt modes between the two sub-families. These are, for the *I*4/*mmm* (RP) and *P*4/*mmm* (DJ) parent, taking *c* as the unique axis: (1) rotation around the *c* axis: X_2_^+^ (RP); M_3_^+^ (DJ). (2) In-phase tilt around the *ab* plane: not possible for RP; X_3_^+^ (DJ). (3) Out-of-phase tilt around the *ab* plane: X_3_^+^ (RP); M_5_^+^ (DJ).

A thorough study of the possible combination of tilt modes in DJ phases was given by Aleksandrov & Bartolomé (2001 ▸) and a briefer version, in the context of hybrid systems by Li, Clulow *et al.* (2019 ▸). Layer-shift modes in these systems are described by symmetry-lowering to monoclinic or triclinic (strain modes, Γ_5_^+^ for example). Note that there is no option for the antiferrodistortive layer-shift mode (corresponding to the M_5_^−^ mode prevalent in Section 3.1, using the *I*4/*mmm* parent) in this section, although many of the structures exhibiting those modes could equally well be derived from the *P*4/*mmm* parent by a Z_5_^−^ mode, which leads to doubling of the number of layers per unit cell.

#### 3.2.1. Structures with no tilts but layer shifts, or tilts but no shifts   

There are two examples (993479 and 1871404) with layer shift, but no tilting; both can be regarded as nRP due to the layer shift (Γ_5_^+^ mode). The second of these has an unusually large octahedral distortion. There is only one example of a structure type with a single octahedral layer per unit cell, with no layer shift but with octahedral tilting: this is perhaps surprising and contrasts with the common occurrence of such tilted/unshifted structure types in inorganic DJ phases. The example (120686) has the M_3_^+^ rotation mode and resulting tilt system *a* ^0^ *a* ^0^ *c*.

#### Structures with metrics 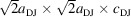 or 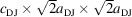


3.2.2.

Apart from the examples above, the vast majority of the structures (66) in this section (commencing 641641) also have the metrics 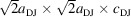 (space group *P*2_1_/*a*) or 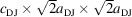 (space group *P*2_1_/*c*). These are essentially the same structure type, containing the M_3_^+^ rotation and M_5_^+^ (a, a) tilt modes [resulting in tilt system *a* ^−^ *a* ^−^ *c*, full model symbol (a|b, b)] and layer shift described, in part, by the β angle of the monoclinic unit cell (Γ_5_^+^ mode). More precisely, the Δ parameters (Δ_1_, Δ_2_) can be derived simply by the equations for the *P*2_1_/*a* examples:

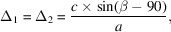

 and, for the *P*2_1_/*c* examples:

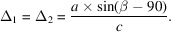

**Figure 10:**
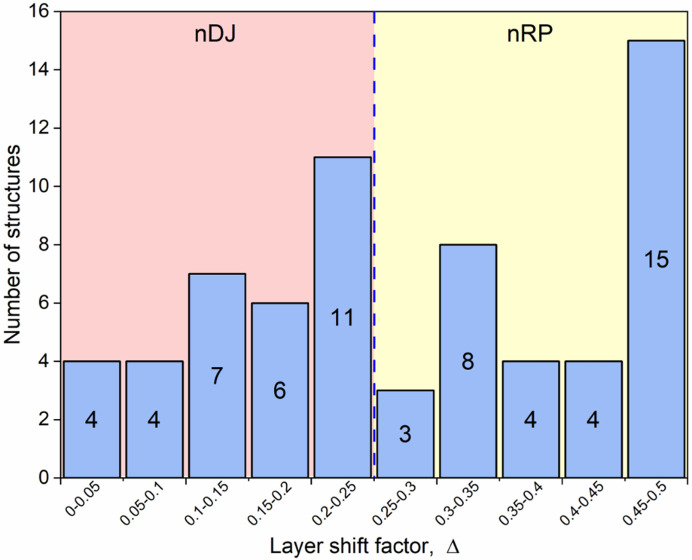
Histogram of layer shift factor values (Δ) for *P*2_1_/*a* and *P*2_1_/*c* structures from Table 4 ▸. Note that Δ consists of two components (Δ_1_, Δ_2_) but by symmetry Δ_1_ = Δ_2_ therefore only one of these is shown.

We recall (Section 3.1.2) that the corresponding tilt system [*a* ^−^ *a* ^−^ *c*/−(*a* ^−^ *a* ^−^)*c*] is also the most common in the RP-derived structures, but in that case it is common for these compounds to retain orthorhombic symmetry and have perfect RP-like staggering of adjacent layers. This is symmetry-disallowed in the single-layer structures, where a combination of these two tilt modes naturally leads to monoclinic symmetry. Nevertheless, it is permissible for these structures to be close to DJ-type, with Δ values close to (0, 0). In fact, the full range of Δ values spanning nDJ to nRP is observed (Table 4 ▸ and Fig. 10 ▸). The difference between the *P*2_1_/*a* and *P*2_1_/*c* types is simply a choice of crystallographic setting, and has no consequence. The remaining structures in this sub-section fall into three sub-groups of the above (*P*2_1_, *P* 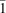 and *P*1). They have the same tilt system, *a* ^−^ *a* ^−^ *c*, but additional distortions; the additional flexibility in unit cells angles describes the tendency towards structures with layer shifts away from the DJ–RP line.

#### 3.2.3. Structures with metrics 2*a*_DJ_ or higher-order supercells   

**Figure 11:**
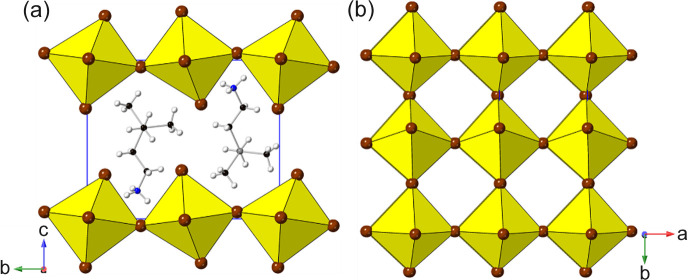
[C_5_H_16_PN]PbBr_4_ (1883687) showing (*a*) tilting around *a* and (*b*) the view down the *c* axis corresponding to the *a* ^+^ *b* ^0^ *c* ^0^ tilt system.

There are a few more complex supercells derived from the single-layer DJ parent. The first (1883687) has the metrics *a*_DJ_ × 2*a*_DJ_ × *c*_DJ_. The key distortion mode is an X_3_^+^(a, 0) tilt mode (Fig. 11 ▸) leading to the tilt system *a* ^+^ *b* ^0^ *c* ^0^. This in itself would lead to a doubled *b* axis and *Pmma* symmetry, but additional minor distortions lower the symmetry to *P*2_1_. The tilt is somewhat reminiscent of 1552603 in Section 3.1.2, but in that case the sense of the *a* ^+^ tilt alternates in adjacent layers [*a* ^+^ *b* ^0^ *c* ^0^/−(*a* ^+^)*b* ^0^ *c* ^0^], leading to a further cell doubling. The remaining structures in this section have a unit cell quadrupled in the layer plane, *i.e.* metrics of the type 2*a*_DJ_ × 2*a*_DJ_ or 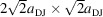 . They typically have low symmetry structures, but still exhibit conventional tilts as some of the largest amplitude modes, together with much smaller additional distortions. Structure 1942547 has the metrics 2*a*_DJ_ × *c*_DJ_ × 2*a*_DJ_, derived from simultaneous rotation [M_3_^+^(a)] and tilt [X_3_^+^(b, c)] modes. This combination leads to the ideal space group *Pmmn* and tilt system *a* ^+^ *b* ^+^ *c*, but the space group is lowered further to polar *Pn* due to very minor distortions from the ideal DJ type. Structures 295291–1963066 have combinations of rotation/tilt [M_3_^+^ and M_5_^+^(a, a)] plus a shift mode leading to the tilt system *a* ^−^ *b* ^0^ *c* and nDJ2 structure type. The metrically related structures 1939809 and 1831525 effectively have a simpler tilt system, *a* ^0^ *a* ^0^ *c*, and DJ type, with the M_5_^+^ and Γ_5_^+^ modes present, but near-zero. Further, minor distortions lower the symmetry slightly from tetragonal to polar monoclinic (see also Section 4). A series of structures (1816279–1846391) have the metrics 2*a*_DJ_ × 2*a*_DJ_ × *c*_DJ_, the first having space group *P*2_1_2_1_2.

**Figure 12:**
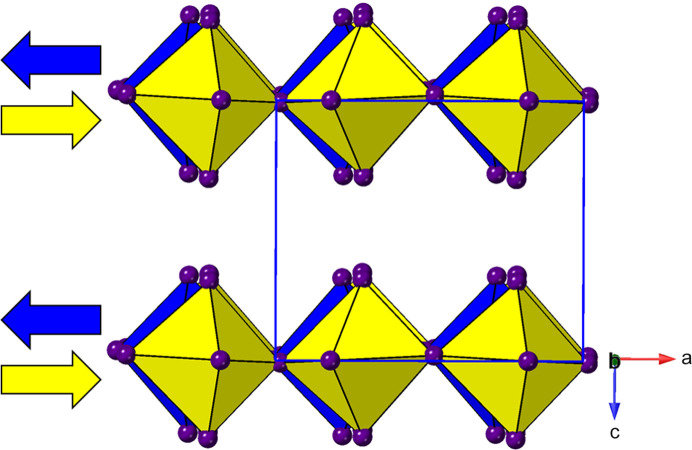
[C_6_H_16_N_2_]PbI_4_ (1816279) viewed along the *c* axis highlighting the fully ‘eclipsed’ DJ arrangement with an antiferrodistortive shift of the octahedra in the layer plane.

The dominant mode is the M_3_^+^ rotation. There is an additional mode, X_3_^−^, which describes an unusual in-plane antiferrodistortive shift of octahedra within each layer; however, the adjacent layers remain perfectly eclipsed, DJ-style (Fig. 12 ▸). The metrically related triclinic structures all have much higher pseudo-symmetry of the inorganic layers than the space group would suggest, typically with only a small number of modes with significant amplitudes. For example, 754084, 1498513 and 616101 have a dominant A_3_^+^ rotation mode: this is unfamiliar, but it is effectively the simple tilt system *a* ^0^ *a* ^0^ *c*/*a* ^0^ *a* ^0^(−*c*) which couples with a layer-shift mode, bringing the structure to nDJ2 type. Without the additional layer shift the A_3_^+^ mode would lead to a doubling of the *c* axis and space group *I*4/*mcm* (reminiscent of the situation in the standard Glazer system *a* ^0^ *a* ^0^ *c* ^−^, Fig. 1 ▸). Structure 2011085 has the same modes but resulting in an nRP structure. Structures 1861843 and 1846392 have the M_3_^+^ rotation mode, with an additional key mode X_2_^−^, which describes an antiferrodistortive displacement of Pb atoms away from their octahedral centres. The combination of these two modes does lead to a 2 × 2 × 1 supercell, but the highest, ideal symmetry is *Pmna*. The orthorhombic structure, 1875165, has the metrics 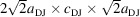 . The M_3_^+^ rotation is again the key mode, and the structure is DJ type, but further complexity arises from intra-octahedral distortions. Finally, 1934874 has a more complex superstructure with a unit cell six times the DJ aristotype ( 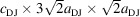 ), involving M_3_^+^ rotations, M_5_^+^ tilts and a rippling or undulation of the [Pb*X*_4_]_∞_ layers along the *b* axis. This type of layer undulation/rippling is discussed further below.

### 3.3. More complex derivatives   

In addition to the final example above, a few more complex structures have unit cells where at least one axis has a metric larger than 2*a*_RP_ or 2*c*_RP_. Ultimately, we find the most complex superstructure reported with a unit-cell volume 16 times the parent RP phase (*i.e.* 32 [Pb*X*_4_] units per unit cell). These complex structures are discussed here and summarized in Table 5 ▸. Unique and unusual features are highlighted. An interesting observation is that the majority of these examples have APb*X*_4_ stoichiometries.

**Figure 13:**
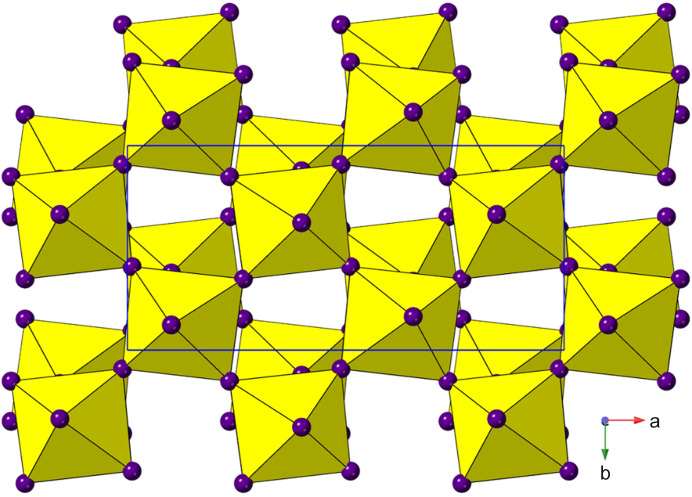
[C_5_H_16_N_2_]PbI_4_ (995699) viewed along the *c* axis highlighting the nDJ arrangement with the *a* ^0^ *a* ^0^ *c*/*a* ^0^ *a* ^0^ *c* ^−^ tilt system, corresponding to the M_5_^−^(b, 0) shift mode and octahedral rotation around the *c* axis attributable to X_2_^+^(0, a).

Several structures have metrics of the type 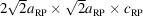 , the first set adopting the space group *Pbca* (995699–1995236). Although the unit-cell size here is four times the size of the RP parent, and therefore contains eight [Pb*X*_4_] units, the relatively high symmetry of these examples still makes an analysis based on *ISODISTORT* very informative. In 995699, we immediately recognize the X_2_^+^(0, a) mode (*i.e.* octahedral rotation around the *c* axis) and the M_5_^−^(b, 0) shift mode, which acts along *b*, leading to an nDJ structure (Fig. 13 ▸) and tilt system *a* ^0^ *a* ^0^ *c*/*a* ^0^ *a* ^0^ *c* ^−^. In addition, the type of ‘rippling’ distortion referred to above is also observed here.

**Figure 14:**
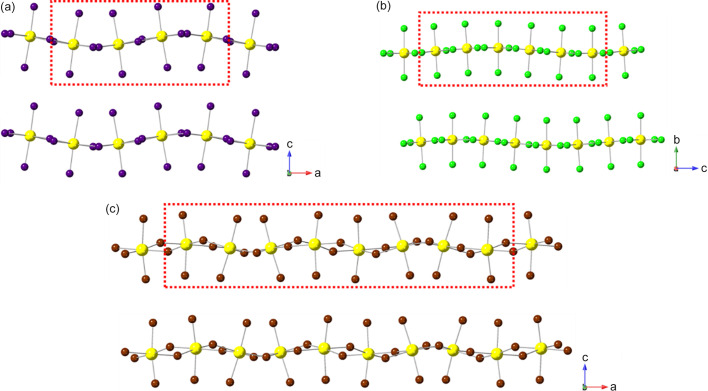
Ball and stick representations of (*a*) [C_5_H_16_N_2_]PbI_4_ (995699), (*b*) [C_2_H_4_N_3_]_2_PbCl_4_ ([TzH]_2_PbCl_4_) and (*c*) [C_9_H_14_N]_2_PbBr_4_ 1995236). These compositions feature doubled, tripled and quadrupled ‘sinusoidal rippling’, respectively. This is highlighted by the dashed red box in each.

Looking down the *b* axis, a ‘sinusoidal’ rippling of the [Pb*X*_4_]_∞_ layers can be seen, with a repeat length of four octahedra [Fig. 14 ▸(*a*)]. Acting alone, the X_2_^+^ and M_5_^−^ modes would produce a unit cell of metrics type 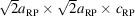 and space group *Pbcm*: we note from Section 3.1.2 that this type of distortion has not been seen in isolation. The full mode label for this X_2_^+^/M_5_^−^ combination is (0, a|b, 0). The additional supercell expansion is caused by the new layer-rippling feature, which is described by modes with labels Δ_3_ and Y_4_. This type of distortion was first noted in our recent example (TzH)_2_PbCl_4_ (Guo, Yang, McNulty *et al.*, 2020 ▸) which has a ‘triple ripple’ rather than a ‘double ripple’ [Fig. 14 ▸(*b*)]. The following six *Pbca* examples adopt the same structure type. Naturally, the amplitudes of octahedral rotation, layer shift and ‘rippling’ are variable within this family with 1838616, for example, showing almost zero octahedral rotation (X_2_^+^) and having a smaller M_5_^−^ shift, leading to nRP status. The next structure in Table 5 ▸ (724584) is a derivative of this structure type but with additional degrees of freedom. Although an X_3_^+^ rotation mode is permitted in this symmetry it has effectively zero amplitude; the resultant structure is nDJ, with tilt system close to *a* ^0^ *a* ^0^ *c*/*a* ^0^ *a* ^0^ *c* ^−^. The structure of 1995236 is related to those above, but with an additional doubling of the *b* axis. Taken together, the three structures in Fig. 14 ▸ show a trend where the layer-rippling feature produces axes of repeat lengths of four, six and eight octahedra, *i.e.* 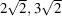 and 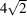 times the parent). Structure 1831521 has 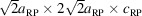 metrics and space group *P*2_1_/*c*. The structure is close to DJ, and has X_3_^+^ tilt, M_5_^−^ shift and more complex distortions.

**Figure 15:**
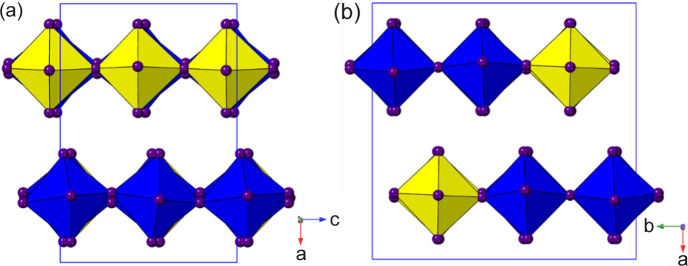
[CH_7_N_2_]_2_PbI_4_ (1937299) showing (*a*) no tilting of the Pb1 (yellow) and out-of-phase tilting of Pb2 (blue) octahedra around *b*. (*b*) Highlights that every third octahedron along the *b* axis is unique, giving rise to unit cell tripling.

Three structures are reported to adopt metrics with six times the volume of the parent RP phase. Two polymorphs (1937299 and 1937297) have a cell with the metrics *c*_RP_ × 3*a*_RP_ × 2*a*_RP_. These are part of a series of phases versus temperature; polymorphism and phase transitions are discussed further in Section 4.2. Structure 1937299 has the space group *Pccn*, with a very unusual tilt system. This is shown in Fig. 15 ▸, where the two crystallographically distinct Pb-centred octahedra are shown in different colours. Viewed down the *c* axis, it can be seen that every third octahedron (Pb1) along the *b* axis is unique, giving rise to unit cell tripling. Viewing along the *b* axis, the underlying reason for this can be seen: the Pb1 octahedron is effectively untilted around *b*, but the two Pb2 octahedra are tilted out of phase relative to each other (*a* ^−^ type). The unusual tilt pattern is described by a C2 (1/3, 1/2, 0) mode, with the other key mode being the expected M_5_^−^ shift.

**Figure 16:**
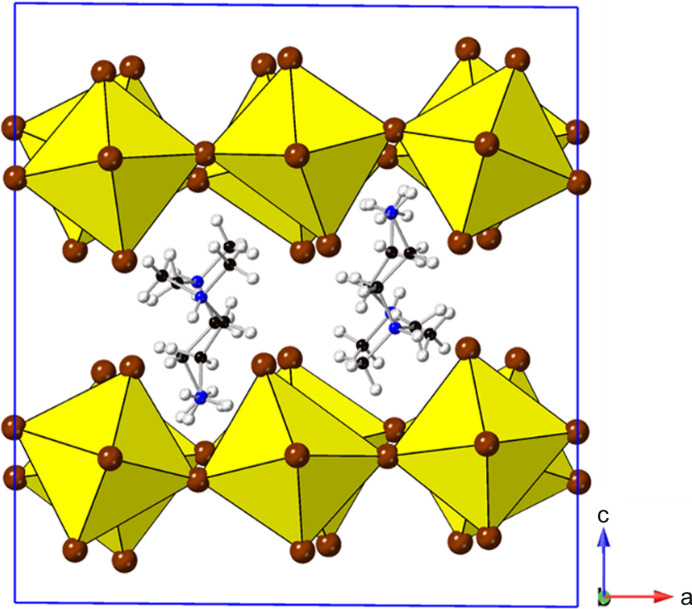
Unusual combination of in-phase and out-of-phase tilts/distortions observed in [C_4_H_14_N_2_]PbBr_4_ (1963065).

Structure 1937297 has essentially the same behaviour, with symmetry lowering caused by ordering of the organic moieties. Similar, unusual and complex tilt systems have been observed previously in traditional oxide perovskites such as NaNbO_3_ (Peel *et al.*, 2012 ▸), but we are unaware of any previous example of this type in layered perovskites. Structure 1963065 has the metrics 3*a*_RP_ × 2*a*_RP_ × *c*_RP_; this has a distinct, but equally unusual combination of in-phase and out-of-phase tilts/distortions (Fig. 16 ▸). Three structures have unit-cell volumes eight times the parent RP cell. Structure 1887281 has the metrics 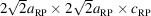 and space group *Pbcn*. The structure is nRP, incorporating a minor sinusoidal undulation along the *a* axis, with a repeat of four octahedra (Δ_3_ and Y_4_ modes). There is also a slight offset of octahedra along *a*, in addition to the M_5_^−^ shift along *b*, which can be compared with the slightly simpler situation in the *Pbca* structures above. Structures 1982717 and 1838611 appear to be polar analogues of this structure.

**Figure 17:**
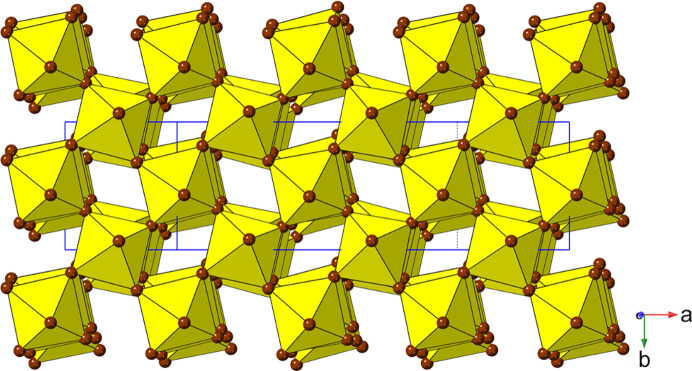
[C_4_H_10_O_2_N]_2_PbBr_4_ (1521060) viewed in the c* direction showing the large degree of pseudo-symmetry present.

The two largest supercell derivatives have unit-cell volumes of 12 and 16 times the RP parent, and both have four octahedral layers per unit cell repeat. The first is 1521060, having the metrics 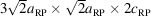 . This complex structure has seven independent Pb sites but does appear to have a relatively high pseudo-symmetry when viewed down the *c* axis (Fig. 17 ▸). The nDJ character is clear from this view, although there are slight shifts, consecutively from one layer to the next. Each layer also has the conventional M_3_^+^ rotation and M_5_^+^ tilt modes when derived from the DJ parent, but there is an additional complex mode which undulates the layers slightly.

**Figure 18:**
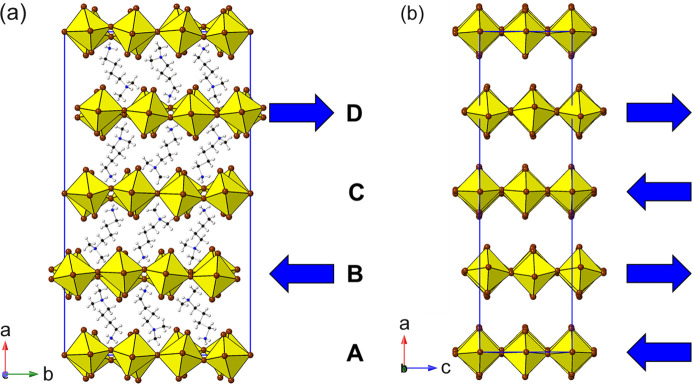
The complex structure of [C_6_H_18_N_2_]PbBr_4_ (1963067) (*a*) viewed down *c*, showing the complex C-mode tilt/distortion and effect of the unique Λ_5_ shift mode (note only the B and D layers are affected by this); (*b*) viewed down *b*, showing the effect of the common M_5_^−^ mode. The result of these superposed shifts is that the A and C layers are perfectly eclipsed (DJ) relative to each other, but all direct neighbouring layers are nDJ2.

The final structure, 1963067, has a unit-cell volume 16 times the RP parent, metrics 2*c*_RP_ × 4*a*_RP_ × 2*a*_RP_, with polar orthorhombic space group *Aba*2. It is perhaps not helpful to describe the full mode details for such complex structures. In fact, the underlying key modes are much simpler, and they reveal an interesting result. The primary-order parameters can be regarded as a C2 (1/4, 1/2, 0) mode: a complex combination of tilts/distortions around *c*, and a complex layer shift, designated Λ_5_, which acts along *b* and shifts only every alternate layer. Acting alone, the C2 mode would actually lead directly to the polar space group *Abm*2 and unit-cell metrics *c*_RP_ × 4*a*_RP_ × 2*a*_RP_ (Fig. 18 ▸). This phase may therefore be regarded as a potential improper ferroelectric. The additional doubling of the *a* axis arises from the combination C2 ⊕ Λ_5_, which directly produces the observed unit-cell metrics and space group. The familiar M_5_^−^ layer shift (acting along *c*) and the Γ_5_^−^ polar modes may be regarded as arising as secondary effects of C2. The C2 mode here is more complex than the C2 (1/3, 1/2, 0) mode observed in 1937299: in that case the C2 mode acting alone also leads to the observed unit-cell metrics and space group, but centrosymmetricity is retained.

## 4. Influence of the interlayer species on the octahedral layer architecture   

### 4.1. General observations   

All of the analysis in Sections 3.1–3.3 is based on the architecture of the [Pb*X*_4_]_∞_ layers only, and makes no reference to the nature of the interlayer organic moieties; indeed, in most cases, it was carried out without knowledge of these! In the crystallography of purely inorganic perovskites and layered perovskites (Benedek *et al.*, 2015 ▸; McCabe *et al.*, 2015 ▸; Aleksandrov & Bartolomé, 2001 ▸; Balachandran *et al.*, 2014 ▸) octahedral tilting is the primary influence on the resultant crystal symmetry, and space groups can be predicted and understood directly from the constituent tilts. In simple ‘Glazer-like’ tilt systems, these space groups are necessarily centrosymmetric (*i.e.* octahedral tilting retains inversion symmetry at the *B* site). However, more complex tilt combinations or combinations of tilts plus cation ordering or other modes can lead to non-centrosymmetric (NCS) space groups, especially in layered systems (Benedek *et al.*, 2012 ▸). Indeed, this fact has been used as a design principle in recent work in the burgeoning field of hybrid improper ferroelectrics [note that ‘hybrid’ does not refer to the inorganic organic system here but to the inducement of polarity by cooperative action of two distinct modes (Benedek & Fennie, 2011 ▸; Benedek *et al.*, 2012 ▸)]. From Tables 1–5 we can see that NCS and polar space groups are not uncommon in hybrid layered perovskites. However, any structure derived from the parent *n* = 1 RP or DJ phase with combinations of only two tilt modes and no layer shift are necessarily centrosymmetric. The structures observed in these compounds are obviously dictated by the total energetics of the system, which will depend on a subtle interplay and co-operation between the molecular moieties and the inorganic framework. An interesting recent example (Park *et al.*, 2019 ▸) which leads to unusual physical properties (Wang *et al.*, 2020 ▸) is that of [C_6_H_16_N_2_]PbI_4_ (1939809 and 1831525, Table 4 ▸), which exhibits an order–disorder transition of the 4-amino­piperidine, but this hardly changes the distortions within the [PbI_4_]_∞_ layer itself. In this section, we will consider structural trends across this family of compounds, and we highlight a few interesting cases of interlayer cation influence on the overall structure. This is not intended to be an exhaustive survey or rationalization, rather, we hope it encourages workers in the field to consider some of the structural principles we have introduced in this paper, in further understanding existing compounds, and possibly in designing-in particular features in future work.

#### . The most common tilt systems   

It can readily be seen from Tables 2 ▸ and 4 ▸ that some unit-cell metrics and space groups occur very frequently, and our analysis in Section 3.3 reveals clearly that these symmetries can be rationalized by the underlying octahedral tilt modes and resultant tilt systems. The two most common are based on 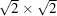 supercells within the layer plane, and space groups *P*2_1_/*a* (equivalently *P*2_1_/*c*) in Table 4 ▸ (commencing No. 641641) and *Pbca* and *C*2/*c* (plus *Cc*) in Table 2 ▸ (commencing 1938881). Together, these examples account for 99 of the total of 260 structures in our review. Each of these types has a combination of two octahedral tilts, specifically out-of-phase tilts out of the [Pb*X*_4_]_∞_ plane and rotations of octahedra perpendicular to this plane, leading to symbols *a* ^−^ *a* ^−^ *c*, *a* ^−^ *a* ^−^ *c*/−(*a* ^−^ *a* ^−^)*c* or *a* ^−^ *a* ^−^ *c*/*a* ^−^ *a* ^−^ *c*. On closer inspection of the types of amine that give rise to these structures, we see a marked tendency in Table 2 ▸ for the *Pbca* structure (*a* ^−^ *a* ^−^ *c*/−(*a* ^−^ *a* ^−^)*c*) to be directed by linear chain amines, RNH_3_^+^, resulting exclusively in *A*_2_Pb*X*_4_ stoichiometry. This corresponds to the ‘traditional’ idea of the RP phase in terms of both stoichiometry and (1/2, 1/2) layer staggering. Interestingly, however, the *a* ^−^ *a* ^−^ *c*/*a* ^−^ *a* ^−^ *c* derivatives (*C*2/*c* and other lower symmetry variants such as 1826587, 1119707, 961380 *etc*.) sometimes arise from aliphatic di­amines, *i.e.* these di­amines apparently introduce a symmetry-lowering layer shift variable and prompt a tendency away from idealized RP towards intermediate layer shifts. For the *a* ^−^ *a* ^−^ *c* systems in Table 4 ▸, a much wider variety of amines is accommodated, most commonly aliphatic amines, but also some aliphatic and other di­amines. It seems that this type of tilt system (which is closely related to the most common GdFeO_3_-type structure in conventional cubic perovskites) is intrinsically quite stable, and robust to many different types of interlayer species, especially given the additional degree of flexibility available via layer-shift modes.

#### 4.1.2. Cl versus Br versus I   

Several amines have been successfully incorporated within chloride, bromide and iodide systems. Some of these adopt the same structure type, some choose different structures, depending on the halide, and some have been shown to display temperature-dependent phase transitions. Obviously, care must always be taken in structural comparisons to ensure that the temperature of structure determination is considered. The amines that form the same structure type for each halide are cyclo­propyl­amine, cyclo­butyl­amine and 4-methyl­benzyl­amine (all of which adopt the most common *a* ^−^ *a* ^−^ *c* structure, *P*2_1_/*c*, Table 4 ▸) and *N,N*-Di­methyl-*p*-phenyl­enedi­amine, which exhibits the more unusual *a* × 2*a* × *c* (*P*2_1_/*n*) structure in Table 3 ▸. The cyclo­propyl­amine/cyclo­butyamine series forms part of a systematic series of studies by Billing & Lemmerer (2007*a* ▸, 2009 ▸) which reveal several interesting trends in terms of the effect of ring size on the position and orientation of the amine between the layers (also revealing that ring sizes larger than six prefer to form structures containing chain-like architectures rather than layered perovskites).

Some examples of amines that form different structures for different halides are cyclo­pentyl­amine (708568, 708562 and 609994), cyclo­hexyl­amine (*e.g.* 708569, 708563 and 609995), 1,6-di­amino­hexane (1914631 and 150501/150502) and cystamine (1841478, 628793 and 724583/4). The first two types here are part of Billing’s studies (Billing & Lemmerer, 2009 ▸, 2007*a* ▸) which draw some interesting observations regarding layer staggering and interlayer distances, which we shall not repeat here. However, it should be noted that the analysis of Billing is incomplete in its interpretation of ‘staggering’ (reported only as either ‘eclipsed’ or ‘staggered’).

In the case of 1,6-di­amino­hexane, the chloride (1914631, Table 2 ▸) adopts an nDJ2 structure type. Although this symmetry permits both octahedral rotations and tilts (tilt system formally *a* ^−^ *a* ^−^ *c*/−(*a* ^−^ *a* ^−^ *c* ^−^) the tilt mode amplitude is near zero, and the only significant modes are the X_2_^+^ rotation and the M_5_^−^ and Γ_5_^+^ shifts. In contrast, the bromide and iodide are isostructural, exhibiting nRP structures, with the common *a* ^−^ *a* ^−^ *c* tilt system (150501/2, Table 4 ▸), differing only slightly in the degree of layer shift. In each case the tilt mode is significant. A key difference between the chloride and the bromide/iodide is that, in the former, the cation adopts a fully stretched (all *trans*) conformation, whereas in the latter pair the terminal C—C bond has a *gauche* kink. This leads to an interesting ‘inverse’ variation in the interlayer distances: Cl (12.32 Å) > Br (12.02 Å) > I (11.86 Å).

For the cystamine derivatives, the differences in crystal structure (triggered by the differing conformations of the cation) have been related in some detail to the optoelectronic properties studied by Krishnamurthy *et al.* (2018 ▸): the chloride displays broadband white luminescence, despite having a low level of structural distortion of the inorganic layers. In fact, the chloride (1841478) exhibits a structure very similar to that of the 1,6-di­amino­hexane analogue just discussed: the tilt system and degree of layer shift are almost the same. Although it might be expected that this similarity arises from the similar nature of the eight-atom linear di­amine chain, in fact the chain conformations are very different, with a much ‘tighter’ configuration here (all torsion angles in the range −73 to −78°). The bromide (628793) has already been discussed in Section 3.1.2, and exhibits the unusual P_4_ tilt mode. Despite the difference apparent in unit-cell metrics and space group compared with the chloride, the resulting layer topology is actually very similar: this time the tilt mode is disallowed, rather than just very small. In contrast, the iodide exists in two polymorphs which co-exist over a wide temperature range (Louvain *et al.*, 2014 ▸). The high-temperature β-phase has the common *a* ^−^ *a* ^−^ *c*, *P*2_1_/*a* structure (724583) with disordered cystamine moieties, whereas the ambient-temperature α-phase has a more complex supercell discussed in Section 3.3. Note that there is a significant change in the inorganic layer from α to β, with a change of rotation mode from *c* to (−*c*), in addition to the changes in the conformation and positioning of the cystamine. Although the tilt system and layer shift in the α-phase result in a similar overall structure to that of the chloride and bromide, the expanded superlattice is caused by ordering of two distinct enantiomeric forms of the cystamine: all these interesting features are discussed in more detail by Louvain *et al.* (2014 ▸)

For *n*-butyl­amine a more diverse range of phases has been reported, which are discussed in Section 4.2.

#### 4.1.3. Homologues and isomers   

Differences in amine structure can have a significant impact on bonding motifs, spatial arrangements and subsequent structural distortions. Due to the large diversity in amines that has been utilized in these materials, only some of the general observations regarding structurally related amines will be discussed. The simplest of these are linear aliphatic amines which have been extensively studied by Billing and co-workers (Lemmerer & Billing, 2012*a* ▸; Billing & Lemmerer, 2007*b* ▸, 2008 ▸). In their work they prepared and characterized compositions of the general formula [C*_n_* H_2*n*+1_NH_3_]_2_PbI_4_, where *n* = 4–18. The ambient-temperature structures for all compositions adopt the RP-type *Pbca* structure [*a* ^−^ *a* ^−^ *c*/−(*a* ^−^ *a* ^−^)*c*] with no real structural change other than an increase in the interlayer distance, consistent with the increase in chain length. While there are phase transitions observed for *n* = 8, 10, 12, 14, 16 and 18 compositions at higher temperature, these are primarily related to changes in conformation of the amines. These structures seem systematic in nature suggesting that the primary effect of increasing chain length is to increase the interlayer distance. Although straight chain alkyl amines can be considered as the simplest amines used in these materials, various other amine types have been utilized. One of the most common structural features of the amines featured here is the inclusion of an aromatic component (>100 examples). Due to the large variability of substitution and type of these, only examples based on or closely related to the (2-amino­ethyl)­pyridine structure will be discussed here (see the supporting information for amine comparison). Recent work by Febriansyah, Lekina *et al.* (2020 ▸) compared the structural effect of positional isomers of the (2-amino­ethyl) substituent to the pyridine ring. In their work they reported an increase in the interlayer I–I separations consistent with the movement of the (2-amino­ethyl) substituent away from the N of the pyridine ring. However, comparison of the tilts and layer shift of these materials shows very little change with respect to the substitution position, with the 2-, 3- and 4-(2-amino­ethyl)­pyridine lead iodide compositions (1944786, 1944783 and 1944782, respectively) all adopting the *a* × 2*a* × *c* (*P*2_1_/*n*) structure (Table 3 ▸) with very similar layer shifts. There is slight variation in the *N*-(2-amino­ethyl)­pyridine composition (1841680, Section 3.1.3) which adopts the *P*2_1_/*c* variant, likely as a result of the closely situated positively charged N atoms.

**Figure 19:**
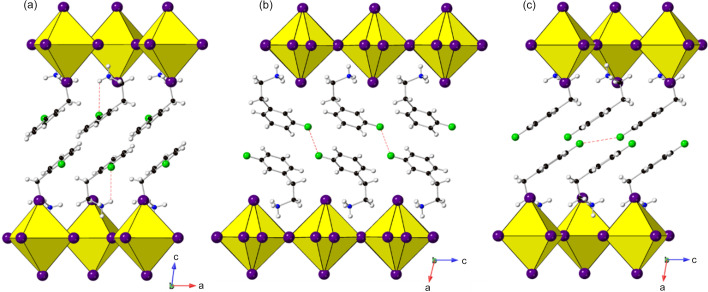
Structures of (*a*) *o*-, (*b*) *m*- and (*c*) *p*-positional isomers of [C_8_H_11_FN]_2_PbI_4_ (1893383, 1893384 and 1488195, respectively). Intramolecular F⋯H—N interactions in (*a*) and intermolecular F⋯F interactions in (*b*) and (*c*) are shown by the dashed red lines. Note the presence of disorder in the I positions in (*b*).

The effect of positional isomerism can be further explored by considering the different structures obtained via *o*-, *m*- or *p*- fluorine substitution in the closely related phenyl­ethyl­amine structure (1893383, 1893384 and 1488195) (Hu *et al.*, 2019*a* ▸,*b* ▸; Slavney *et al.*, 2017 ▸). Unlike the related (2-amino­ethyl)­pyridine compositions there are significant differences in the structures adopted depending on the position of the fluorine atom (Fig. 19 ▸). In both the *m*- and *p*-substituted compositions the amines are arranged with the aromatic group oriented in the middle of the organic bilayer region, with configurations that maximize intermolecular F⋯F interactions (F⋯F ≃ 3.04 and 3.42 Å, respectively). Both structures appear to be nRP with a greater degree of layer shift in the *m*-substituted composition, consistent with the enhanced F⋯F interaction present. In the *o*-substituted composition it is no longer possible for the amines to orient to maximize F⋯F interactions; however, the proximity of the electronegative F group to the NH_3_^+^ of the ethyl­ammonium chain induces a conformational change to maximize the F⋯H—N interaction (∼3.25 Å) resulting in a layer shift close to DJ2. This difference between closely related amines highlights the importance of the amine with regards to the structural behaviour observed.

#### 4.1.4. *A*Pb*X*_4_ and *AA*′Pb*X*_4_ compositions   

It can be noted that the vast majority of the structures presented here (190 out of 260) correspond to the *A*_2_Pb*X*_4_ composition. A smaller number (67) correspond to *A*Pb*X*_4_ types, where *A* is typically an aliphatic di­amine or a cyclic amine containing a single N, with an amine side-chain. A much smaller number (3) correspond to mixed-cation systems, *i.e.* *AA*′Pb*X*_4_. There is clearly considerable scope for further imaginative synthesis in targeting these less-represented stoichiometries. For the *A*Pb*X*_4_ types templated by linear di­amines [H_3_NC*_n_* H_2*n*_NH_3_]^2+^, early work (Lemmerer & Billing, 2012*a* ▸) showed a trend whereby the parity of the carbon number (*i.e. n* = odd or even) dictated the tilt system of the inorganic layers with compositions of *n* = 8, 10 and 12 adopting the *a* ^−^ *a* ^−^ *c* system. However, unlike the linear amine chains discussed in Section 4.1.3, there is a significant change in the layer shift of the analogous di­amines. As the chain length increases, the degree of layer shift correspondingly decreases from nRP (Δ = 0.42, 0.42) for *n* = 8 to almost perfectly DJ in *n* = 12 (Δ = 0.01, 0.01). Due to the increased chain length, it appears that the degree of octahedral tilting required to optimize hydrogen bonding is reduced, which subsequently reduces the need for layer shift in these materials. The absence of this effect in the related *A*_2_Pb*X*_4_ compositions is likely due to the presence of the ‘bilayer’ of amines in the interlayer site that cannot optimize the hydrogen bonding in the same way.

The few examples of *AA*′Pb*X*_4_ structures all exhibit a common feature of alternating cations in the interlayer sites along the [Pb*X*_4_]_∞_ direction and seem to adopt the nDJ2 structure. Both [CH_6_N][CH_6_N_3_]PbI_4_ and [CH_6_N][Cs]PbBr_4_ (1588974 and 1552603, respectively) adopt the *Imma* structure corresponding to the *a* ^+^ *b* ^0^ *c* ^0^/–(*a* ^+^)*b* ^0^ *c* ^0^ tilt system. Although the [CH_6_N_3_][Cs]PbI_4_ structure (1552604) shares similarities with the two related compositions, both M_5_^−^ and Γ_5_^+^ layer-shift modes are introduced and a more complex tilt system incorporating a rotation around the *c* axis is observed resulting in the adoption of the *Pnnm* space group.

### 4.2. Polymorphs and phase transitions   

We have already highlighted interesting polymorphic behaviour in the cystamine-based family. In addition, there are many more examples of competition between different phases (such as the layered versus chain options for Billing’s cyclic amines) or polymorphs [such as 1963065, which adopts both (001)- and (110)-oriented layered perovskite polymorphs (Mao *et al.*, 2017 ▸)]. Here we discuss a few further examples of temperature phase transitions, and some unusual examples of chemically induced structural rearrangements. In some papers, structural phase transitions are reported from the perspective of thermodynamic and spectroscopic behaviour, but full single-crystal determinations are not available for both phases (*e.g.* 1938883). Likewise, there are some papers that do appear to report single-crystal determinations at two temperatures but, unfortunately, they have not been deposited at the CCDC.

The most common cause of temperature-induced structural phase transitions in these materials appears to be order–disorder behaviour of the organic moieties. The study of fluorinated benzyl­amines (BA) by Shi *et al.* (2019 ▸) is a nice example. All four compounds, benzyl­amine, 2-fluoro­benzyl­amine, 3-fluoro­benzyl­amine and 4-fluoro­benzyl­amine (Table 1 ▸) adopt the aristotype RP structure *I*4/*mmm* in their highest temperature phase (*i.e.* above ambient) with disordered organic moieties. However, on cooling both BA itself and 2-FBA transform to the polar space group *Cmc*2_1_ via introduction of the X_2_^+^ rotation and a polar mode (Table 2 ▸, 120685 and 1944743). On the contrary, despite 3-FBA and 4-FBA also undergoing tilt transitions on cooling (1944741, *a* ^−^ *a* ^−^ *c* and 1944739, *a* ^0^ *a* ^0^ *c*/*a* ^0^ *a* ^0^ *c*, respectively) they retain centrosymmetricity. In each case the low-temperature phase has ordered organic moieties, but it is suggested that the lower steric hindrance in BA and 2-FBA permits the differences in crystal packing. Ferroelectricity has been confirmed in 2-FBA, and Xiong’s group have also observed related behaviour in several other fluorinated amine systems (Liao *et al.*, 2015 ▸; Sha *et al.*, 2019 ▸; Shi *et al.*, 2019 ▸). 3-Fluoro-*N*-methyl­benzyl­amine also displays an order–disorder transition, but retains centrosymmetricity in both phases, this time from an unusual high-temperature phase with a layer shift but no tilts (1852626, Table 2 ▸) to a room-temperature (RT) phase (1845548) with the X_3_^+^ tilt mode added. There are several other examples of order–disorder transitions, involving changes of tilt system, for example structures 1417497 (high-temperature, *Cmca*, Table 2 ▸) to 1417496 (low-temperature, *P*2_1_/*c*, Table 4 ▸). Structure 1962913 (Section 3.1.2) is an unusual case, which shows a transition from a high-symmetry phase with no octahedral tilting or layer shifts, but displays antiferrodistortive displacements of the Pb atoms and disordered piperidinium cations at high temperature (352 K) to a layered phase with five-coordinate Pb at low temperature (Chai *et al.*, 2020 ▸).

**Figure 20:**
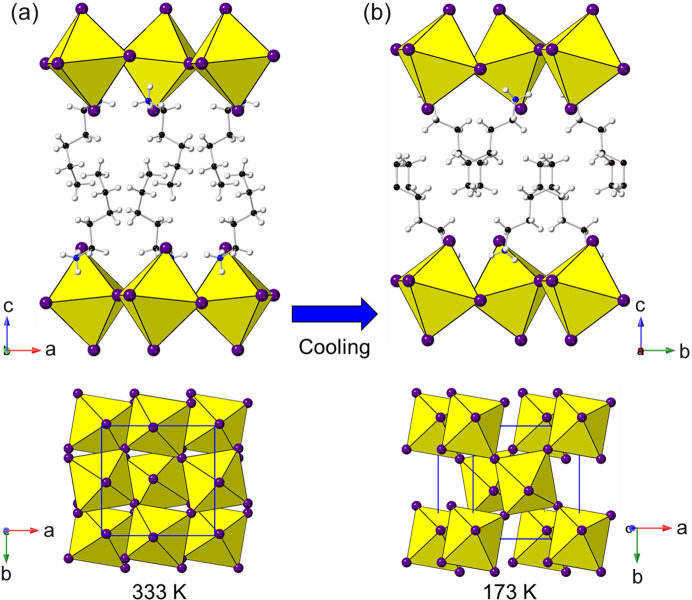
High (665693) and low (665691) temperature phases of [C_5_H_14_N]_2_PbI_4_ adopting *Pbca* and *P*2_1_/*a* structures, respectively. Note the change in degree of layer shift corresponding to the change in amine packing.

Billing described several phase transitions in the series of alkyl­ammonium-templated materials (RNH_3_)_2_PbI_4_, with *n*-butyl, *n*-pentyl or *n*-hexyl chains (Billing & Lemmerer, 2007*b* ▸). These do not involve disorder of the alkyl chain, but instead exhibit changes in packing of these chains, which induce shift/tilt transitions of the [PbI_4_]_∞_ layers. For example, structure 665693 (Table 2 ▸) transforms to 665691 (Table 4 ▸) on cooling, with a change from RP type, *Pbca*, *a* ^−^ *a* ^−^ *c*/−(*a* ^−^ *a* ^−^)*c* to nRP type, *P*2_1_/*c*, *a* ^−^ *a* ^−^ *c* (Fig. 20 ▸). Several independent studies have been carried out on (*n*-BA)_2_Pb*X*_4_ (*n*-BA = *n*-butyl­amine). In the case of the iodide two phases exist (665689), both adopting the common RP-related *Pbca* structure, but differing in the orientation and hydrogen bonding of the *n*-BA moiety (Billing & Lemmerer, 2007*b* ▸). The bromide (1455948) also adopts the *Pbca* structure at 100 K, but has been reported in a lower symmetry version of this structure (1903531) at room temperature. The chloride has three reported phases from different studies: 1952028, 2016195 and 2003637, all of which adopt an RP-like structure. Ji *et al.* (2019 ▸) report ferroelectric behaviour and a transition from *Cmca* to *Cmc*2_1_ at *T*_C_ = 328 K, with order–disorder of the *n*-BA, but no change in tilt system. Tu *et al.* (2020 ▸) also confirm *Cmc*2_1_ at RT, whereas McClure *et al.* (2020 ▸) suggest the lower symmetry space group *Pbca*, but this study is based on powder diffraction only.

(MHy)_2_PbI_4_ (MHy = methyl­hydrazinium) is of interest as MHy is the smallest organic cation to be incorporated into any structure in this review. It undergoes three phase transitions versus temperature, mediated by order–disorder of MHy, which also leads to some interesting physical properties (Mączka *et al.*, 2019 ▸). The phases are 1937296 (Table 2 ▸) which displays only the M_5_^−^ shift mode (note there is reported to be an isostructural phase transition between two phases with this symmetry), 1937299 and 1937297 (Table 5 ▸), both of which display the unique ‘triple tilt’ C-mode (see Section 3.3 and Fig. 15 ▸).

In addition to the differences in conformation of linear-chain amines which can affect Cl versus Br versus I analogues (Section 4.1.2), these changes may also occur within the same compound, as a function of temperature. An example is (DAB)_2_PbCl_4_ (DAB = 1,4-di­amino­butane). Courseille *et al.* (1994 ▸) reported a complex structure at ambient temperature (1305732), and suggested a simpler structure (DJ-like, *a* ^−^ *a* ^−^ *c*) above RT; however, a full structural analysis of this phase is required.

Finally, there are intriguing cases where an *in situ* chemical reaction takes place, for example, the reaction of alykynyl or alkenyl amines with Br_2_ or I_2_ (Solis-Ibarra *et al.*, 2015 ▸; Solis-Ibarra & Karunadasa, 2014 ▸) leads to the addition of Br_2_ across the unsaturated C—C bond. In both cases (955778 to 1048947 and 955776 to 955777) the tilt system (the apparently very robust *a* ^−^ *a* ^−^ *c*, Table 4 ▸) remains unchanged, but the chemical changes are manifest both in changes of the [Pb*X*_4_]_∞_ interlayer distances (approximate increase of around 3–5 Å in these cases) and in changes in layer shift from close to nDJ type towards DJ2 type. There are also reported examples of intercalation of intact I_2_ molecules in similar reactions; unfortunately, full single crystal details are not available.

## 5. Summary and conclusions   

The main aim of this comprehensive review is to provide a more systematic approach to understanding the crystallography of layered LHPs by introducing the concepts of symmetry mode analysis. Octahedral tilting and layer shifts are shown to be the key distortion modes underlying the nature of the inorganic perovskite-like layers in this family of materials. However, the complexity of these systems goes far beyond the octahedral tilt systems traditionally seen in purely inorganic layered perovskites. First and foremost, we use our analysis to show the relationships between apparently diverse groups of structures and compositions, by classifying in terms of unit-cell metrics and key distortion modes, relative to idealized parent phases. We hope this approach, which may be unfamiliar to many current workers in the field, will be of use in informing and directing future work. Although we have tried to be exhaustive in the analysis of each individual structure type, we have by no means analysed the underlying reasons for the adoption of the particular distorted variants for specific compositions. We have merely highlighted a few trends and interesting cases relating the nature of the interlayer species to the behaviour of the inorganic layer. For example, we have shown that certain tilt systems, such as *a* ^−^ *a* ^−^ *c*, are very robust and resilient to changes in the nature of the interlayer species, being able to accommodate different species by adjustments to tilt amplitudes and layer shift factors (from DJ towards RP and even DJ2) whist still retaining essentially the same structure type. We have also highlighted some less common distortion modes, such as antiferrodistortive Pb displacements, tilt systems (such as triple and quadruple repeats) that go beyond the standard Glazer-like systems, and undulations (‘ripples’) of the perovskite-like layers themselves. Of particular note are complex tilt modes not generally found in conventional perovskites [but see, for example the work by Peel *et al.* (2012 ▸) and Dixon & Lightfoot (2018 ▸)], such as the C2 modes in 1937299 and 1963067, which may naturally lead to non-centrosymmetric or polar space groups.

It is clear that much more structural analysis can be done on the vast array of existing structures, for example, we have not referred at all to the various octahedral distortion indices or interlayer penetration effects that are typically quoted in relation to physical properties, and which are undoubtedly an important factor in determining those properties. We hope our novel view of the structural chemistry of this important family of materials will enhance understanding of both structure–composition and structure–property relationships, thus adding to the design and development of materials with enhanced features of interest.

## Supplementary Material

Supporting figures and tables. DOI: 10.1107/S2052252521005418/yc5031sup1.pdf

## Figures and Tables

**Table 1:** Summary of experimentally known RP-derived structures with *a*_RP_ × *a*_RP_ × *c*_RP_ unit-cell metrics The parent structure type is the RP phase (*I*4/*mmm*). The ‘Type’ column in all Tables is merely intended to highlight the structures with divalent [A] or mixed-cation [A][A′] stoichiometries.

CCDC number	Amine	Type	Formula	Space group	Key modes	Tilt modes	Reference
1934895	4,4-Di­fluoro­piperidine		[C_5_H_10_F_2_N]_2_PbI_4_ 453 K	*I*4/*mmm*	–	*a* ^0^ *a* ^0^ *c* ^0^	Zhang, Song, Chen *et al.* (2020 ▸)
1944745	Benzyl­amine		[C_7_H_10_N]_2_PbCl_4_ 493 K	*I*4/*mmm*	–	*a* ^0^ *a* ^0^ *c* ^0^	Shi *et al.* (2019 ▸)
1944744	2-Fluoro­benzyl­amine		[C_7_H_9_FN]_2_PbCl_4_ 463 K	*I*4/*mmm*	–	*a* ^0^ *a* ^0^ *c* ^0^	Shi *et al.* (2019 ▸)
1944742	3-Fluoro­benzyl­amine		[C_7_H_9_FN]_2_PbCl_4_ 473 K	*I*4/*mmm*	–	*a* ^0^ *a* ^0^ *c* ^0^	Shi *et al.* (2019 ▸)
1944740	4-Fluoro­benzyl­amine		[C_7_H_9_FN]_2_PbCl_4_ 493 K	*I*4/*mmm*	–	*a* ^0^ *a* ^0^ *c* ^0^	Shi *et al.* (2019 ▸)
1992694	4,4-Di­fluoro­hexa­hydro­azepine		[C_6_H_12_F_2_N]_2_PbI_4_ 493 K	*I* 42*m*	–	*a* ^0^ *a* ^0^ *c* ^0^	Chen, Song, Zhang, Zhang *et al.* (2020 ▸)
1992693	4,4-Di­fluoro­hexa­hydro­azepine		[C_6_H_12_F_2_N]_2_PbI_4_ 423 K	*Imm*2	Γ_5_^−^	*a* ^0^ *a* ^0^ *c* ^0^	Chen, Song, Zhang, Zhang *et al.* (2020 ▸)
1937296	Methyl­hydrazine		[CH_7_N_2_]_2_PbI_4_ RT	*Pmmn*	M_5_^−^	*a* ^0^ *a* ^0^ *c* ^0^	Mączka *et al.* (2019 ▸)
1211182	1,4-Di­methyl­piperazine	[A]	[C_6_H_16_N_2_]PbBr_4_	*P*2_1_	M_5_^−^	*a* ^0^ *a* ^0^ *c* ^0^	Bonamartini, Corradi *et al.* (2001 ▸)
1186561	1-Phenyl­ethyl­amine		[C_8_H_12_N]_2_PbI_4_	*C*2/*m*	M_5_^−^	*a* ^0^ *a* ^0^ *c* ^0^	Calabrese *et al.* (1991 ▸)

**Table 2:** Summary of experimentally known structures with two octahedral layers per unit cell (derived from RP parent, *I*4/*mmm*) and \sqrt {2}a_{\rm RP} \times \sqrt {2}a_{\rm RP} cell metrics in the layer plane Separate tilt patterns are given for both layers when relevant; a minus sign precedes the second pattern if it acts opposite to the first pattern.

						Key modes					
CCDC number	Amine	Type	Formula	Space group	Layer direction	Tilt	Layer shift	Tilt system	Layer shift factor, Δ	Structure type	Reference
1852626	3-Fluoro-*N*-methyl­benzyl­amine		[C_8_H_11_FN]_2_PbBr_4_	*Cmcm*	*c*	–	M_5_^−^	*a* ^0^ *a* ^0^ *c* ^0^	0.15, 0.15	nDJ	Hao *et al.* (2019 ▸)
641642	2-Bromo­ethyl­amine		[C_2_H_7_BrN]_2_PbI_4_ 293 K	*C*2/*c*	*c*	–	M_5_^−^, Γ_5_^+^	*a* ^0^ *a* ^0^ *c* ^0^	0.5, 0.15	nDJ2	Sourisseau *et al.* (2007 ▸)
167103	2,2′-Bi­imidazole	[A]	[C_6_H_8_N_4_]PbI_4_	*C*2/*c*	*c*	–	M_5_^−^, Γ_5_^+^	*a* ^0^ *a* ^0^ *c* ^0^	0.47, 0.12	nDJ2	Tang *et al.* (2001 ▸)
1863837	Butan-2-amine		[C_4_H_12_N]_2_PbBr_4_	*P*4_2_/*ncm*	*c*	X_3_^+^	–	*a* ^−^ *b* ^0^c^0^/b^0^ *a* ^−^ *c* ^0^	0.5, 0.5	RP	Li, Dunlap-Shohl *et al.* (2019 ▸)
1934896	4,4-Di­fluoro­piperidine		[C_5_H_10_F_2_N]_2_PbI_4_ 300 K	*Aba*2	*b*	X_3_^+^	–	*a* ^−^ *a* ^−^c^0^/−(*a* ^−^ *a* ^−^)*c* ^0^	0.5, 0.5	RP	Zhang, Song, Chen *et al.* (2020 ▸)
1992692	4,4-Di­fluoro­hexa­hydro­azepine		[C_6_H_12_F_2_N]_2_PbI_4_ 343 K	*Cmc*2­_1_	*b*	X_3_^+^	–	*a* ^−^ *a* ^−^c^0^/−(*a* ^−^ *a* ^−^)*c* ^0^	0.5, 0.5	RP	Chen, Song, Zhang, Zhang *et al.* (2020 ▸)
2016195	Butyl­amine		[C_4_H_12_N]_2_PbCl_4_ 330 K	*Cmca*	*a*	X_2_^+^	–	*a* ^0^ *a* ^0^ *c*/*a* ^0^ *a* ^0^ *c*	0.5, 0.5	RP	Ji *et al.* (2019 ▸)
1417497	Iso­butyl­amine		[C_4_H_12_N]_2_PbBr_4_ 393 K	*Cmca*	*a*	X_2_^+^	–	*a* ^0^ *a* ^0^ *c*/*a* ^0^ *a* ^0^ *c*	0.5, 0.5	RP	Wang *et al.* (2015 ▸)
708568	Cyclo­pentyl­amine		[C_5_H_12_N]_2_PbCl_4_	*Cmca*	*a*	X_2_^+^	–	*a* ^0^ *a* ^0^ *c*/*a* ^0^ *a* ^0^ *c*	0.5, 0.5	RP	Billing & Lemmerer (2009 ▸)
1409219	Cyclo­hexyl­amine		[C_6_H_14_N]_2_PbBr_4_ 383 K	*Cmca*	*a*	X_2_^+^	–	*a* ^0^ *a* ^0^ *c*/*a* ^0^ *a* ^0^ *c*	0.5, 0.5	RP	Ye *et al.* (2016 ▸)
1409221	Cyclo­hexyl­amine		[C_6_H_14_N]_2_PbI_4_	*Cmca*	*a*	X_2_^+^	–	*a* ^0^ *a* ^0^ *c*/*a* ^0^ *a* ^0^ *c*	0.5, 0.5	RP	Ye *et al.* (2016 ▸)
1042749	Benzyl­amine		[C_7_H_10_N]_2_PbBr_4_	*Cmca*	*a*	X_2_^+^	–	*a* ^0^ *a* ^0^ *c*/*a* ^0^ *a* ^0^ *c*	0.5, 0.5	RP	Liao *et al.* (2015 ▸)
1482272	(Cyclo­hexyl­methyl)­amine		[C_7_H_16_N]_2_PbBr_4_ 433 K	*Cmca*	*a*	X_2_^+^	–	*a* ^0^ *a* ^0^ *c*/*a* ^0^ *a* ^0^ *c*	0.5, 0.5	RP	Li *et al.* (2017 ▸)
805432	Octyl­amine		[C_8_H_20_N]_2_PbI_4_ 314 K	*Acam*	*c*	X_2_^+^	–	*a* ^0^ *a* ^0^ *c*/*a* ^0^ *a* ^0^ *c*	0.5, 0.5	RP	Lemmerer & Billing (2012*b* ▸)
805440	Decyl­amine		[C_10_H_24_N]_2_PbI_4_ 343 K	*Acam*	*c*	X_2_^+^	–	*a* ^0^ *a* ^0^ *c*/*a* ^0^ *a* ^0^ *c*	0.5, 0.5	RP	Lemmerer & Billing (2012*b* ▸)
2003637	Butyl­amine		[C_4_H_12_N]_2_PbCl_4_ 293 K	*Cmc*2_1_	*a*	X_2_^+^	–	*a* ^0^ *a* ^0^ *c*/*a* ^0^ *a* ^0^ *c*	0.5, 0.5	RP	Tu *et al.* (2020 ▸)
1965897	4-Amino­tetra­hydro­pyran		[C_5_H_12_ON]_2_PbBr_4_	*Cmc*2_1_	*a*	X_2_^+^	–	*a* ^0^ *a* ^0^ *c*/*a* ^0^ *a* ^0^ *c*	0.5, 0.5	RP	Chen, Song, Zhang, Li *et al.* (2020 ▸)
1904977	4,4-Di­fluoro­cyclo­hexyl­amine		[C_6_H_12_F_2_N]_2_PbI_4_ RT	*Cmc*2_1_	*a*	X_2_^+^	–	*a* ^0^ *a* ^0^ *c*/*a* ^0^ *a* ^0^ *c*	0.5, 0.5	RP	Sha *et al.* (2019 ▸)
708563	Cyclo­hexyl­amine		[C_6_H_14_N]_2_PbBr_4_ 173 K	*Cmc*2_1_	*a*	X_2_^+^	–	*a* ^0^ *a* ^0^ *c*/*a* ^0^ *a* ^0^ *c*	0.5, 0.5	RP	Billing & Lemmerer (2009 ▸)
1894433	Hexyl­amine		[C_6_H_16_N]_2_PbI_4_	*Cmc*2_1_	*a*	X_2_^+^	–	*a* ^0^ *a* ^0^ *c*/*a* ^0^ *a* ^0^ *c*	0.5, 0.5	RP	Jung (2019 ▸)
1944743	2-Fluoro­benzyl­amine		[C_7_H_9_FN]_2_PbCl_4_ 300 K	*Cmc*2_1_	*a*	X_2_^+^	–	*a* ^0^ *a* ^0^ *c*/*a* ^0^ *a* ^0^ *c*	0.5, 0.5	RP	Shi *et al.* (2019 ▸)
1542460	Benzyl­amine		[C_7_H_10_N]_2_PbBr_4_ RT	*Cmc*2_1_	*a*	X_2_^+^	–	*a* ^0^ *a* ^0^ *c*/*a* ^0^ *a* ^0^ *c*	0.5, 0.5	RP	Du *et al.* (2017 ▸)
120685	Benzyl­amine		[C_7_H_10_N]_2_PbCl_4_ 293 K	*Cmc*2_1_	*a*	X_2_^+^	–	*a* ^0^ *a* ^0^ *c*/*a* ^0^ *a* ^0^ *c*	0.5, 0.5	RP	Braun & Frey (1999 ▸)
1542462	1-(2-Naphthyl)-methyl­amine		[C_11_H_12_N]_2_PbBr_4_	*Cmc*2_1_	*a*	X_2_^+^	–	*a* ^0^ *a* ^0^ *c*/*a* ^0^ *a* ^0^ *c*	0.5, 0.5	RP	Du *et al.* (2017 ▸)
1940831	2-(4-Bi­phenyl)­ethyl­amine		[C_14_H_16_N]_2_PbI_4_	*Cmc*2_1_	*a*	X_2_^+^	–	*a* ^0^ *a* ^0^ *c*/*a* ^0^ *a* ^0^ *c*	0.5, 0.5	RP	Venkatesan *et al.* (2019 ▸)
237190	2-Hy­droxy­ethyl­amine		[C_2_H_8_ON]_2_PbBr_4_ RT	*Pbcn*	*c*	X_3_^+^	M_5_^−^	*a* ^−^ *a* ^−^c^0^/−(*a* ^−^ *a* ^−^)*c* ^0^	0.35, 0.35	nRP	Mercier *et al.* (2004 ▸)
2016665	4-Fluoro-*N*-methyl­aniline		[C_7_H_9_FN]_2_PbI_4_	*Pbcn*	*c*	X_3_^+^	M_5_^−^	*a* ^−^ *a* ^−^c^0^/−(*a* ^−^ *a* ^−^)*c* ^0^	0.38, 0.38	nRP	Jang & Kaminsky (2020*a* ▸)
1845548	3-Fluoro-*N*-methyl­benzyl­amine		[C_8_H_11_FN]_2_PbBr_4_ RT	*Pbcn*	*c*	X_3_^+^	M_5_^−^	*a* ^−^ *a* ^−^c^0^/−(*a* ^−^ *a* ^−^)*c* ^0^	0.17, 0.17	nDJ	Hao *et al.* (2019 ▸)
1883324	3-Chloro-*N*-methyl­benzyl­amine		[C_8_H_11_ClN]_2_PbI_4_	*Pbcn*	*c*	X_3_^+^	M_5_^−^	*a* ^−^ *a* ^−^c^0^/−(*a* ^−^ *a* ^−^)*c* ^0^	0.21, 0.21	nDJ	Yao (2018 ▸)
1975113	3-Bromo-*N*-methyl­benzyl­amine		[C_8_H_11_BrN]_2_PbCl_4_	*Pbcn*	*c*	X_3_^+^	M_5_^−^	*a* ^−^ *a* ^−^c^0^/−(*a* ^−^ *a* ^−^)*c* ^0^	0.13, 0.13	nDJ	Yao (2020*b* ▸)
1975109	3-Bromo-*N*-methyl­benzyl­amine		[C_8_H_11_BrN]_2_PbBr_4_	*Pbcn*	*c*	X_3_^+^	M_5_^−^	*a* ^−^ *a* ^−^c^0^/−(*a* ^−^ *a* ^−^)*c* ^0^	0.17, 0.17	nDJ	Yao (2020*a* ▸)
1938882	2,2,2-Tri­fluoro­ethyl­amine		[C_2_H_5_F_3_N]_2_PbBr_4_	*Pnma*	*b*	X_2_^+^	M_5_^−^	*a* ^0^ *a* ^0^ *c*/*a* ^0^ *a* ^0^ *c*	0.48, 0.48	nRP	Luo, Guo, Xiao *et al.* (2019 ▸)
1938883	2-Fluoro­ethyl­amine		[C_2_H_7_FN]_2_PbCl_4_	*Pnma*	*b*	X_2_^+^	M_5_^−^	*a* ^0^ *a* ^0^ *c*/*a* ^0^ *a* ^0^ *c*	0.30, 0.30	nRP	Lermer, Birkhold *et al.* (2016 ▸)
705087	2-Cyano­ethyl­amine		[C_3_H_7_N_2_]_2_PbI_4_	*Pnma*	*b*	X_2_^+^	M_5_^−^	*a* ^0^ *a* ^0^ *c*/*a* ^0^ *a* ^0^ *c*	0.35, 0.35	nRP	Mercier *et al.* (2009 ▸)
1181686	Propyl­amine		[C_3_H_10_N]_2_PbCl_4_	*Pnma*	*b*	X_2_^+^	M_5_^−^	*a* ^0^ *a* ^0^ *c*/*a* ^0^ *a* ^0^ *c*	0.32, 0.32	nRP	Meresse & Daoud (1989 ▸)
1495877	3-Bromo­pyridine		[C_5_H_5_BrN]_2_PbBr_4_	*Pnma*	*b*	X_2_^+^	M_5_^−^	*a* ^0^ *a* ^0^ *c*/*a* ^0^ *a* ^0^(-*c*)	0.02, 0.02	nDJ	Gómez *et al.* (2016 ▸)
1944739	4-Fluoro­benzyl­amine		[C_7_H_9_FN]_2_PbCl_4_ 300 K	*Pnma*	*b*	X_2_^+^	M_5_^−^	*a* ^0^ *a* ^0^ *c*/*a* ^0^ *a* ^0^ *c*	0.33, 0.33	nRP	Shi *et al.* (2019 ▸)
956549	1-Cyclo­hexyl­ethyl­amine		[C_8_H_18_N]_2_PbBr_4_	*Pnma*	*b*	X_2_^+^	M_5_^−^	*a* ^0^ *a* ^0^ *c*/*a* ^0^ *a* ^0^ *c*	0.40, 0.40	nRP	Lemmerer & Billing (2013 ▸)
781211	1,4-Bis(amino­methyl)­cyclo­hexane	[A]	[C_8_H_20_N_2_]PbCl_4_	*Pnma*	*b*	X_2_^+^	M_5_^−^	*a* ^0^ *a* ^0^ *c*/*a* ^0^ *a* ^0^(-*c*)	0.17, 0.17	nDJ	Rayner & Billing (2010*c* ▸)
1914148	Ethyl-1,2-di­amine	[A]	[C_2_H_10_N_2_]PbI_4_	*Pbcm*	*c*	X_2_^+^	M_5_^−^	*a* ^0^ *a* ^0^ *c*/*a* ^0^ *a* ^0^ *c*	0.35, 0.35	nRP	Faza­yeli *et al.* (2020 ▸)
1938881	2,2-Di­fluoro­ethyl­amine		[C_2_H_6_F_2_N]_2_PbBr_4_	*Pbca*	*c*	X_2_^+^, X_3_^+^	–	*a* ^−^ *a* ^−^ *c*/−(*a* ^−^ *a* ^−^)*c*	0.5, 0.5	RP	Luo, Guo, Xiao *et al.* (2019 ▸)
267398	4-Amino­butanoic acid		[C_4_H_10_O_2_N]_2_PbI_4_	*Pbca*	*c*	X_2_^+^, X_3_^+^	–	*a* ^−^ *a* ^−^ *c*/−(*a* ^−^ *a* ^−^)*c*	0.5, 0.5	RP	Mercier (2005 ▸)
1952028	Butyl­amine		[C_4_H_12_N]_2_PbCl_4_	*Pbca*	*c*	X_2_^+^, X_3_^+^	–	*a* ^−^ *a* ^−^ *c*/−(*a* ^−^ *a* ^−^)*c*	0.5, 0.5	RP	McClure *et al.* (2020 ▸)
1455948	Butyl­amine		[C_4_H_12_N]_2_PbBr_4_ 100 K	*Pbca*	*c*	X_2_^+^, X_3_^+^	–	*a* ^−^ *a* ^−^ *c*/−(*a* ^−^ *a* ^−^)*c*	0.5, 0.5	RP	Dou *et al.* (2015 ▸)
665689	Butyl­amine		[C_4_H_12_N]_2_PbI_4_	*Pbca*	*c*	X_2_^+^, X_3_^+^	–	*a* ^−^ *a* ^−^ *c*/−(*a* ^−^ *a* ^−^)*c*	0.5, 0.5	RP	Billing & Lemmerer (2007*b* ▸)
1935994	2-Thio­phene­methyl­amine		[C_5_H_8_SN]_2_PbCl_4_	*Pbca*	*c*	X_2_^+^, X_3_^+^	–	*a* ^−^ *a* ^−^ *c*/−(*a* ^−^ *a* ^−^)*c*	0.5, 0.5	RP	Wei *et al.* (2019 ▸)
1935993	2-Thio­phene­methyl­amine		[C_5_H_8_SN]_2_PbBr_4_	*Pbca*	*c*	X_2_^+^, X_3_^+^	–	*a* ^−^ *a* ^−^ *c*/−(*a* ^−^ *a* ^−^)*c*	0.5, 0.5	RP	Wei *et al.* (2019 ▸)
187952	2-Thio­phene­methyl­amine		[C_5_H_8_SN]_2_PbI_4_	*Pbca*	*c*	X_2_^+^, X_3_^+^	–	*a* ^−^ *a* ^−^ *c*/−(*a* ^−^ *a* ^−^)*c*	0.5, 0.5	RP	Zhu *et al.* ( 2002 ▸)
665693	Pentyl­amine		[C_5_H_14_N]_2_PbI_4_ 333 K	*Pbca*	*c*	X_2_^+^, X_3_^+^	–	*a* ^−^ *a* ^−^ *c*/−(*a* ^−^ *a* ^−^)*c*	0.5, 0.5	RP	Billing & Lemmerer (2007*b* ▸)
1904976	4,4-Di­fluoro­cyclo­hexyl­amine		[C_6_H_12_F_2_N]_2_PbI_4_ 398 K	*Pbca*	*c*	X_2_^+^, X_3_^+^	–	*a* ^−^ *a* ^−^ *c*/−(*a* ^−^ *a* ^−^)*c*	0.5, 0.5	RP	Sha *et al.* (2019 ▸)
609995	Cyclo­hexyl­amine		[C_6_H_14_N]_2_PbI_4_	*Pbca*	*c*	X_2_^+^, X_3_^+^	–	*a* ^−^ *a* ^−^ *c*/−(*a* ^−^ *a* ^−^)*c*	0.5, 0.5	RP	Billing & Lemmerer (2007*a* ▸)
746130	6-Iodo­hexyl­amine		[C_6_H_15_IN]_2_PbI_4_	*Pbca*	*c*	X_2_^+^, X_3_^+^	–	*a* ^−^ *a* ^−^ *c*/−(*a* ^−^ *a* ^−^)*c*	0.5, 0.5	RP	Lemmerer & Billing (2010 ▸)
665695	Hexyl­amine		[C_6_H_16_N]_2_PbI_4_ 293 K	*Pbca*	*c*	X_2_^+^, X_3_^+^	–	*a* ^−^ *a* ^−^ *c*/−(*a* ^−^ *a* ^−^)*c*	0.5, 0.5	RP	Billing & Lemmerer (2007*b* ▸)
1493135	Benzyl­amine		[C_7_H_10_N]_2_PbI_4_	*Pbca*	*c*	X_2_^+^, X_3_^+^	–	*a* ^−^ *a* ^−^ *c*/−(*a* ^−^ *a* ^−^)*c*	0.5, 0.5	RP	Kamminga *et al.* (2016 ▸)
805428	Heptyl­amine		[C_7_H_18_N]_2_PbI_4_ 278 K	*Pbca*	*c*	X_2_^+^, X_3_^+^	–	*a* ^−^ *a* ^−^ *c*/−(*a* ^−^ *a* ^−^)*c*	0.5, 0.5	RP	Lemmerer & Billing (2012*b* ▸)
805431	Octyl­amine		[C_8_H_20_N]_2_PbI_4_ 293 K	*Pbca*	*c*	X_2_^+^, X_3_^+^	–	*a* ^−^ *a* ^−^ *c*/−(*a* ^−^ *a* ^−^)*c*	0.5, 0.5	RP	Lemmerer & Billing (2012*b* ▸)
805434	Nonyl­amine		[C_9_H_22_N]_2_PbI_4_ 293 K	*Pbca*	*c*	X_2_^+^, X_3_^+^	–	*a* ^−^ *a* ^−^ *c*/−(*a* ^−^ *a* ^−^)*c*	0.5, 0.5	RP	Lemmerer & Billing (2012*b* ▸)
1859258	Deca-3,5-diyn-1-amine		[C_10_H_16_N]_2_PbBr_4_	*Pbca*	*c*	X_2_^+^, X_3_^+^	–	*a* ^−^ *a* ^−^ *c*/−(*a* ^−^ *a* ^−^)*c*	0.5, 0.5	RP	Ortiz-Cervantes *et al.* (2018 ▸)
805436	Decyl­amine		[C_10_H_24_N]_2_PbI_4_ 268 K	*Pbca*	*c*	X_2_^+^, X_3_^+^	–	*a* ^−^ *a* ^−^ *c*/−(*a* ^−^ *a* ^−^)*c*	0.5, 0.5	RP	Lemmerer & Billing (2012*b* ▸)
692951	Do­decyl­amine		[C_12_H_28_N]­_2_PbI_4_ 293 K	*Pbca*	*c*	X_2_^+^, X_3_^+^	–	*a* ^−^ *a* ^−^ *c*/−(*a* ^−^ *a* ^−^)*c*	0.5, 0.5	RP	Billing & Lemmerer (2008 ▸)
1934872	2-[(5-Meth­oxy­naphthalen-1-yl)­oxy]ethan-1-amine		[C_13_H_16_O_2_N]_2_PbI_4_	*Pbca*	*c*	X_2_^+^, X_3_^+^	–	*a* ^−^ *a* ^−^ *c*/−(*a* ^−^ *a* ^−^)*c*	0.5, 0.5	RP	Passarelli *et al.* (2020 ▸)
692953	Tetra­decyl­amine		[C_14_H_32_N]­_2_PbI_4_ 293 K	*Pbca*	*c*	X_2_^+^, X_3_^+^	–	*a* ^−^ *a* ^−^ *c*/−(*a* ^−^ *a* ^−^)*c*	0.5, 0.5	RP	Billing & Lemmerer (2008 ▸)
692955	Hexa­decyl­amine		[C_16_H_36_N]­_2_PbI_4_ 293 K	*Pbca*	*c*	X_2_^+^, X_3_^+^	–	*a* ^−^ *a* ^−^ *c*/−(*a* ^−^ *a* ^−^)*c*	0.5, 0.5	RP	Billing & Lemmerer (2008 ▸)
692957	Octa­decyl­amine		[C_18_H_40_N]­_2_PbI_4_ 293 K	*Pbca*	*c*	X_2_^+^, X_3_^+^	–	*a* ^−^ *a* ^−^ *c*/−(*a* ^−^ *a* ^−^)*c*	0.5, 0.5	RP	Billing & Lemmerer (2008 ▸)
1826587	2-Methyl­pentane-1,5-di­amine	[A]	[C_6_H_18_N_2_]PbCl_4_	*Cc*	*a*	X_2_^+^, X_4_^+^	Γ_5_^+^	*a* ^−^ *a* ^−^ *c*/*a* ^−^ *a* ^−^ *c*	0.30, 0.30	nRP	Wang *et al.* (2018 ▸)
1119686	2-Methyl­pentane-1,5-di­amine	[A]	[C_6_H_18_N_2_]PbBr_4_	*Cc*	*a*	X_2_^+^, X_4_^+^	Γ_5_^+^	*a* ^−^ *a* ^−^ *c*/*a* ^−^ *a* ^−^ *c*	0.26, 0.26	nRP	Corradi *et al.* (1999 ▸)
1869673	1,9-di­amino­nonane	[A]	[C_9_H_24_N_2_]PbI_4_	*Cc*	*a*	X_2_^+^, X_4_^+^	Γ_5_^+^	*a* ^−^ *a* ^−^ *c*/*a* ^−^ *a* ^−^ *c*	0.42, 0.42	nRP	Li *et al.* (2018 ▸)
1840806	Pyrene-*O*-propyl­amine		[C_19_H_18_ON]_2_PbI_4_	*Cc*	*a*	X_2_^+^, X_4_^+^	Γ_5_^+^	*a* ^−^ *a* ^−^ *c*/*a* ^−^ *a* ^−^ *c*	0.27, 0.27	nRP	Passarelli *et al.* (2018 ▸)
853207	1,4-Di­amino­butane	[A]	[C_4_H_14_N_2_]PbI_4_	*C*2/*c*	*a*	X_2_^+^, X_4_^+^	Γ_5_^+^	*a* ^−^ *a* ^−^ *c*/*a* ^−^ *a* ^−^ *c*	0.22, 0.22	nDJ	Lemmerer & Billing (2012*a* ▸)
1521067	2-Methyl­pentane-1,5-di­amine	[A]	[C_6_H_18_N_2_]PbBr_4_	*C*2/*c*	*a*	X_2_^+^, X_4_^+^	Γ_5_^+^	*a* ^−^ *a* ^−^ *c*/*a* ^−^ *a* ^−^ *c*	0.24, 0.24	nDJ	Smith *et al.* (2017 ▸)
1977186	4-Chloro­phenethyl­amine		[C_8_H_11_ClN]_2_PbI_4_	*C*2/*c*	*a*	X_2_^+^, X_4_^+^	Γ_5_^+^	*a* ^−^ *a* ^−^ *c*/*a* ^−^ *a* ^−^ *c*	0.40, 0.40	nRP	Straus *et al.* (2019 ▸)
1977187	4-Bromo­phenethyl­amine		[C_8_H_11_BrN]_2_PbI_4_	*C*2/*c*	*a*	X_2_^+^, X_4_^+^	Γ_5_^+^	*a* ^−^ *a* ^−^ *c*/*a* ^−^ *a* ^−^ *c*	0.44, 0.44	nRP	Straus *et al.* (2019 ▸)
956552	(*RS*)-1-Cyclo­hexyl­ethyl­amine		[C_8_H_18_N]_2_PbCl_4_	*C*2/*c*	*a*	X_2_^+^, X_4_^+^	Γ_5_^+^	*a* ^−^ *a* ^−^ *c*/*a* ^−^ *a* ^−^ *c*	0.49, 0.49	nRP	Lemmerer & Billing (2013 ▸)
1856671	Hexa­decyl­amine		[C_16_H_36_N]_2_PbI_4_	*Pca*2_1_	*b*	X_2_^+^, X_3_^+^	M_5_^−^	*a* ^−^ *a* ^−^ *c*/−(*a* ^−^ *a* ^−^)*c*	0.5, 0.5	RP	Hong *et al.* (2019 ▸)
1934873	4-[(Naphthalen-1-yl)­oxy]butyl-1-amine		[C_14_H_18_ON]_2_PbI_4_	*Pca*2_1_	*c*	X_2_^+^, X_3_^+^	M_5_^−^	*a* ^−^ *a* ^−^ *c*/−(*a* ^−^ *a* ^−^)*c*	0.08, 0.08	nDJ	Passarelli *et al.* (2020 ▸)
1903531	Butyl­amine		[C_4_H_12_N]_2_PbBr_4_ RT	*P*2_1_/*c*	*a*	X_2_^+^, X_3_^+^	M_5_^−^	*a* ^−^ *a* ^−^ *b*/−(*a* ^−^ *a* ^−^)*c*		nRP	Gong *et al.* (2018 ▸)
1119707	Propane-1,3-di­amine	[A]	[C_3_H_12_N_2_]PbCl_4_	*P*2_1_2_1_2_1_	*a*	X_2_^+^, X_3_^+^	M_5_^−^	*a* ^−^ *a* ^−^ *c*/−(*a* ^−^ *a* ^−^)*c*	0.13, 0.13	nDJ	Corradi *et al.* (1999 ▸)
607740, 607741	(R)- or (S)-1-Phenyl­ethyl­amine		[C_8_H_12_N]_2_PbI_4_	*P*2_1_2_1_2_1_	*c*	X_2_^+^, X_3_^+^	M_5_^−^	*a* ^−^ *a* ^−^ *c*/−(*a* ^−^ *a* ^−^)*c*	0.29, 0.29	nRP	Billing & Lemmerer (2006*b* ▸)
1942543	3-(Amino­ethyl)­pyridine	[A]	[C_6_H_10_N_2_]PbI_4_	*Pn*	*c*	X_2_^+^, X_4_^+^	M_5_^−^, Γ_5_^+^			nDJ	Li, Ke *et al.* (2019 ▸)
2016669	3-Fluoro-*N*-methyl­aniline		[C_7_H_9_FN]_2_PbI_4_	*Pn*	*b*	X_3_^+^, X_4_^+^	M_5_^−^, Γ_5_^+^			nRP	Jang & Kaminsky (2020*d* ▸)
659021	*m*-Phenyl­enedi­amine	[A]	[C_6_H_10_N_2_]PbCl_4_	*P*2/*c*	*a*	X_2_^+^, X_4_^+^	M_5_^−^, Γ_5_^+^			nDJ	Dobrzycki & Woźniak (2008 ▸)
1305732	1,4-Di­amino­butane	[A]	[C_4_H_14_N_2_]PbCl_4_	*P*2_1_/*c*	*c*	X_2_^+^, X_3_^+^	M_5_^−^, Γ_5_^+^			nDJ	Courseille *et al.* (1994 ▸)
961380	*N*-Methyl­propane-1,3-di­amine	[A]	[C_4_H_14_N_2_]PbBr_4_	*P*2_1_/*c*	*c*	X_2_^+^, X_3_^+^	M_5_^−^, Γ_5_^+^			nDJ2	Dohner, Hoke & Karunadasa (2014 ▸)
1841478	2,2′-Di­thio­bis­(ethyl­ammonium)	[A]	[C_4_H_14_S_2_N_2_]PbCl_4_	*P*2_1_/*c*	*c*	X_2_^+^, X_3_^+^	M_5_^−^, Γ_5_^+^			nDJ2	Krishnamurthy *et al.* (2018 ▸)
1877264	1-Methyl­piperidin-4-amine	[A]	[C_6_H_16_N_2_]PbI_4_	*P*2_1_/*c*	*c*	X_2_^+^, X_3_^+^	M_5_^−^, Γ_5_^+^			nDJ	Pei *et al.* (2019 ▸)
											
1914631	1,6-Di­amino­hexane	[A]	[C_6_H_18_N_2_]PbCl_4_	*P*2_1_/*c*	*c*	X_2_^+^, X_3_^+^	M_5_^−^, Γ_5_^+^			nDJ2	Maris (1996 ▸)
1521055	3-(2-Amino­ethyl)­aniline	[A]	[C_8_H_14_N_2_]PbBr_4_	*P*2_1_/*c*	*c*	X_2_^+^, X_3_^+^	M_5_^−^, Γ_5_^+^			nDJ	Smith *et al.* (2017 ▸)
1525376	Histamine	[A]	[C_5_H_11_N_3_]PbI_4_	*P*2_1_/*n*	*b*	X_2_^+^, X_4_^+^	M_5_^−^, Γ_5_^+^			nDJ2	Mao *et al.* (2016 ▸)
1999302	5-Amino­pentanoic acid		[C_5_H_12_O_2_N]_2_PbBr_4_	*P*2_1_/*n*	*c*	X_2_^+^, X_4_^+^	M_5_^−^, Γ_5_^+^			nDJ2	Krummer (2020 ▸)
1819854	4-Fluoro­benzyl­amine		[C_7_H_9_FN]_2_PbI_4_	*P*2_1_/*n*	*c*	X_3_^+^, X_4_^+^	M_5_^−^, Γ_5_^+^			nDJ	Hao *et al.* (2018 ▸)
1934876	3-(Penta­chloro­phen­oxy)­propyl-1-amine		[C_9_H_9_Cl_5_ON]_2_PbI_4_	*P*2_1_/*n*	*c*	X_3_^+^, X_4_^+^	M_5_^−^, Γ_5_^+^			nRP	Passarelli *et al.* (2020 ▸)

**Table 3:** Experimental structures with two octahedral layers per unit cell (derived from RP parent, *I*4/*mmm*) and at least one axis in the layer plane doubled Separate tilt patterns are given for both layers when relevant; a minus sign precedes the second pattern if it acts opposite to the first pattern.

						Key modes					
CCDC number	Amine	Type	Formula	Space group	Metrics	Tilt	Layer shift	Tilt system	Layer shift factor, Δ	Structure type	Reference
1962913	Piperidine		[C_5_H­_12_N]_2_PbCl_4_	*C*2/*c*	*c* × *a* × 2*a*	–	Γ_5_^+^	none, see text		nRP	Chai *et al.* (2020 ▸)
1507154	Benzimidazole		[C_7_H_7_N_2_]_2_PbI_4_	*C*2/*c*	*c* × *a* × 2*a*	–	Γ_5_^+^	–		nDJ2	Lermer, Harm *et al.* (2016 ▸)
1893384	3-Fluoro­phenethyl­amine		[C_8_H_11_FN]_2_PbI_4_	*C*2/*c*	*c* × *a* × 2*a*	–	Γ_5_^+^	–		nRP	Hu *et al.* (2019*b* ▸)
1840805	Pyrene-*O*-butyl­amine		[C_20_H_20_ON]_2_PbI_4_	*C*2/*c*	*c* × *a* × 2*a*	–	Γ_5_^+^	–		nDJ2	Passarelli *et al.* (2018 ▸)
1846391	2-(3″′,4′-Di­methyl-[2,2′:5′,2″:5″,2″′-quaterthio­phen]-5-yl)ethan-1-amine		[C_20_H_20_S_4_N]_2_PbI_4_	*C*2/*c*	*c* × *a* × 2*a*	–	Γ_5_^+^	–		nRP	Gao *et al.* (2019 ▸)
1938883	2-Fluoro­ethyl­amine		[C_2_H_7_FN]_2_PbBr_4_	*Pnma*	2*a* × *c* × *a*	–	M_5_^−^	–	0.17, 0.43	nDJ2	Luo, Guo, Xiao *et al.* (2019 ▸)
746131	2-Bromo­ethyl­amine		[C_2_H_7_BrN]_2_PbI_4_ 73 K	*Pnma*	2*a* × *c* × *a*	–	M_5_^−^	–	0.16, 0.42	nDJ2	Lemmerer & Billing (2010 ▸)
1962914	Piperidine		[C_5_H_12_N]_2_PbBr_4_	*Pnma*	2*a* × *c* × *a*	–	M_5_^−^	–	0.48, 0.49	nRP	Chai *et al.* (2020 ▸)
641644	2-Chloro­ethyl­amine		[C_2_H_7_ClN]_2_PbI_4_	*Pbnm*	*a* × 2*a* × *c*	–	M_5_^−^	–	0.44, 0.17	nDJ2	Sourisseau *et al.* (2007 ▸)
641643	2-Bromo­ethyl­amine		[C_2_H_7_BrN]_2_PbI_4_ 293 K	*Pbnm*	*a* × 2*a* × *c*	–	M_5_^−^	–	0.46, 0.17	nDJ2	Sourisseau *et al.* (2007 ▸)
1588974	Methyl­amine, and guanidine	[A][A′]	[CH_6_N][CH_6_N_3_]PbI_4_	*Imma*	*a* × 2*a* × *c*	T_3_^+^	–	*a* ^+^ *b* ^0^ *c* ^0^/–(*a* ^+^)*b* ^0^ *c* ^0^	0.5, 0	nDJ2	Soe *et al.* (2017 ▸)
1552603	Guanidine and Cs	[A][A′]	[CH_6_N_3_]CsPbBr_4_	*Imma*	*a* × 2*a* × *c*	T_3_^+^	–	*a* ^+^ *b* ^0^ *c* ^0^/–(*a* ^+^)*b* ^0^ *c* ^0^	0.5, 0	nDJ2	Nazarenko *et al.* (2017 ▸)
1915486	4-(2-Amino­ethyl)­pyridine	[A]	[C_7_H_12_N_2_]PbBr_4_	*P*2_1_/*n*	*a* × 2*a* × *c*	Σ	M_5_^−^, Γ_5_^+^	complex: see text		nRP	Febriansyah, Giovanni *et al.* (2020 ▸)
1944786	2-(2-Amino­ethyl)­pyridine	[A]	[C_7_H_12_N_2_]PbI_4_	*P*2_1_/*n*	*a* × 2*a* × *c*	Σ	M_5_^−^, Γ_5_^+^			nDJ2	Febriansyah, Lekina *et al.* (2020 ▸)
1944783	3-(2-Amino­ethyl)­pyridine	[A]	[C_7_H_12_N_2_]PbI_4_	*P*2_1_/*n*	*a* × 2*a* × *c*	Σ	M_5_^−^, Γ_5_^+^			nDJ2	Febriansyah, Lekina *et al.* (2020 ▸)
1944782	4-(2-Amino­ethyl)­pyridine	[A]	[C_7_H_12_N_2_]PbI_4_	*P*2_1_/*n*	*a* × 2*a* × *c*	Σ	M_5_^−^, Γ_5_^+^			nDJ2	Febriansyah, Lekina *et al.* (2020 ▸)
659016	*N,N*-Di­methyl-*p*-phenyl­enedi­amine	[A]	[C_8_H_14_N_2_]PbCl_4_	*P*2_1_/*n*	*a* × 2*a* × *c*	Σ	M_5_^−^, Γ_5_^+^			nDJ2	Dobrzycki & Woźniak (2008 ▸)
1572154	*N,N*-Di­methyl-*p*-phenyl­enedi­amine	[A]	[C_8_H_14_N_2_]PbBr_4_	*P*2_1_/*n*	*a* × 2*a* × *c*	Σ	M_5_^−^, Γ_5_^+^			nDJ	Hautzinger *et al.* (2017 ▸)
1572156	*N,N*-Di­methyl-*p*-phenyl­enedi­amine	[A]	[C_8_H_14_N_2_]PbI_4_	*P*2_1_/*n*	*a* × 2*a* × *c*	Σ	M_5_^−^, Γ_5_^+^			nDJ	Hautzinger *et al.* (2017 ▸)
1841680	*N*-(2-Amino­ethyl)­pyridine	[A]	[C_7_H_12_N_2_]PbI_4_	*P*2_1_/*c*	*a* × *c* × 2*a*					nDJ2	Febriansyah *et al.* (2019 ▸)
628793	Cystamine	[A]	[C_4_H_14_S_2_N_2_]PbBr_4_	*C*2/*c*	2*a* × 2*a* × *c*	P_4_	M_5_^−^, Γ_5_^+^	*a* ^0^ *a* ^0^ *c*/*a* ^0^ *a* ^0^(–)*c*		nDJ2	Louvain *et al.* (2007 ▸)
1985833	1,2,4-Triazole		[C_2_H_4_N_3_]_2_PbBr_4_	*C*2/*c*	2*a* × 2*a* × *c*	P_4_, P_5_	M_5_^−^, Γ_5_^+^	complex: see text		nDJ	Guo, Yang, Biberger *et al.* (2020 ▸)
1043214	(2-Thio­phene)­ethyl­amine		[C_6_H_10_SN]_2_PbI_4_	*Cc*	2*a* × 2*a* × *c*	P_4_	M_5_^−^, Γ_5_^+^			nDJ2	Dammak *et al.* (2016 ▸)
1841681	Phenethyl­amine		[C_8_H_12_N]_2_PbI_4_	*Cc*	2*a* × 2*a* × *c*	P_4_	M_5_^−^, Γ_5_^+^			nDJ2	Febriansyah *et al.* (2019 ▸)
1840808	Naphthalene-*O*-ethyl­amine		[C_12_H_14_ON]_2_PbI_4_	*Cc*	2*a* × 2*a* × *c*	P_4_	M_5_^−^, Γ_5_^+^			nRP	Passarelli *et al.* (2018 ▸)
1840802	Pyrene-*O*-ethyl­amine		[C_18_H_16_ON]_2_PbI_4_	*Cc*	2*a* × 2*a* × *c*	P_4_	M_5_^−^, Γ_5_^+^			nRP	Passarelli *et al.* (2018 ▸)
2016668	*N*-Methyl­aniline		[C_7_H_10_N]_2_PbI_4_	*Cc*	2*a* × 2*a* × *c*	P_5_	M_5_^−^, Γ_5_^+^			nRP	Jang & Kaminsky (2020*c* ▸)
1542463	2-(2-Naphthyl)­ethyl­amine		[C_12_H_14_N]_2_PbI_4_	*Pn*	2*a* × 2*a* × *c*	P_4_	M_5_^−^, Γ_5_^+^			nRP	Du *et al.* (2017 ▸)
1552604	Guanidine and Cs	[A][A′]	[CH_6_N_3_]CsPbI_4_	*Pnnm*	2*a* × *c* × 2*a*	X_2_^+^, X_3_^+^	M_5_^−^, Γ_5_^+^			nDJ2	Nazarenko *et al.* (2017 ▸)
708569	Cyclo­hexyl­amine		[C_6_H_14_N]_2_PbCl_4_	*P*2_1_/*m*	2*a* × *c* × 2*a*	X_2_^+^	M_5_^−^			nRP	Billing & Lemmerer (2009 ▸)
1841683	*N*-(2-Amino­ethyl)­piperidine	[A]	[C_7_H_18_N_2_]PbI_4_	*P*2_1_/*n*	2*a* × *c* × 2*a*	Σ_3_	M_5_^−^			nDJ	Febriansyah *et al.* (2019 ▸)

**Table 4:** Summary of experimentally known structures with one octahedral layer per unit cell (derived from DJ parent, *P*4/*mmm*) Separate tilt patterns are given for both layers when relevant. [C_4_H_6_O_2_] = di­hydro­furan-2(3*H*)-one is solvated into the crystal structure.

						Key modes					
CCDC number	Amine	Type	Formula	Space group	Metrics	Tilt	Layer shift	Tilt system	Layer shift factor, Δ	Structure type	Reference
993479	2,2′-(Ethyl­ene­dioxy)­bis­(ethyl­amine)	[A]	[C_6_H_18_O_2_N_2_]PbCl_4_	*C*2	\sqrt {2}a \times \sqrt{2}a \times c	–	Γ_5_^+^	*a* ^0^ *a* ^0^ *c* ^0^	0.37, 0.37	nRP	Dohner, Jaffe *et al.* (2014 ▸)
1871404	3,5-Di­hydro­imidazo[4,5-*f*]benzimidazole	[A]	[C_8_H_8_N_4_]PbI_4_	*C*2/*m*	\sqrt {2}a \times \sqrt{2}a \times c	–	Γ_5_^+^	*a* ^0^ *a* ^0^ *c* ^0^	0.31, 0.31	nRP	Zimmermann *et al.* (2019 ▸)
120686	1-(2-Naphthyl)-methyl­amine		[C_11_H_12_N]_2_PbCl_4_	*Pbam*	\sqrt {2}a \times \sqrt{2}a \times c	M_3_^+^	–	*a* ^0^ *a* ^0^ *c*	0, 0	DJ	Braun & Frey (1999 ▸)
641641	2-Iodo­ethyl­amine		[C_2_H_7_IN]_2_PbI_4_ 293 K	*P*2_1_/*a*	\sqrt {2}a \times \sqrt{2}a \times c	M_3_^+^, M_5_^+^	Γ_5_^+^	*a* ^−^ *a* ^−^ *c*	0.19, 0.19	nDJ	Sourisseau *et al.* (2007 ▸)
237189	2-Hy­droxy­ethyl­amine		[C_2_H_8_ON]_2_PbI_4_ 293 K	*P*2_1_/*a*	\sqrt {2}a \times \sqrt{2}a \times c	M_3_^+^, M_5_^+^	Γ_5_^+^	*a* ^−^ *a* ^−^ *c*	0.20, 0.20	nDJ	Mercier *et al.* (2004 ▸)
724583	2,2′-Disulfanediyldiethanamine	[A]	[C_4_H_14_S_2_N_2_]PbI_4_ β-phase	*P*2_1_/*a*	\sqrt {2}a \times \sqrt{2}a \times c	M_3_^+^, M_5_^+^	Γ_5_^+^	*a* ^−^ *a* ^−^ *c*	0.33, 0.33	nRP	Louvain *et al.* (2014 ▸)
665691	Pentyl­amine		[C_5_H_14_N]_2_PbI_4_ 173 K	*P*2_1_/*a*	\sqrt {2}a \times \sqrt{2}a \times c	M_3_^+^, M_5_^+^	Γ_5_^+^	*a* ^−^ *a* ^−^ *c*	0.33, 0.33	nRP	Billing & Lemmerer (2007*b* ▸)
665694	Hexyl­amine		[C_6_H_16_N]_2_PbI_4_ 173 K	*P*2_1_/*a*	\sqrt {2}a \times \sqrt{2}a \times c	M_3_^+^, M_5_^+^	Γ_5_^+^	*a* ^−^ *a* ^−^ *c*	0.06, 0.06	nDJ	Billing & Lemmerer (2007*b* ▸)
805427	Heptyl­amine		[C_7_H_18_N]_2_PbI_4_ 253 K	*P*2_1_/*a*	\sqrt {2}a \times \sqrt{2}a \times c	M_3_^+^, M_5_^+^	Γ_5_^+^	*a* ^−^ *a* ^−^ *c*	0.24, 0.24	nDJ	Lemmerer & Billing (2012*b* ▸)
141193	4-Methyl­benzyl­amine		[C_8_H_12_N]_2_PbCl_4_	*P*2_1_/*a*	\sqrt {2}a \times \sqrt{2}a \times c	M_3_^+^, M_5_^+^	Γ_5_^+^	*a* ^−^ *a* ^−^ *c*	0.21, 0.21	nDJ	Papavassiliou *et al.* (2000 ▸)
141192	4-Methyl­benzyl­amine		[C_8_H_12_N]_2_PbBr_4_	*P*2_1_/*a*	\sqrt {2}a \times \sqrt{2}a \times c	M_3_^+^, M_5_^+^	Γ_5_^+^	*a* ^−^ *a* ^−^ *c*	0.32, 0.32	nRP	Papavassiliou *et al.* (2000 ▸)
141190	4-Methyl­benzyl­amine		[C_8_H_12_N]_2_PbI_4_	*P*2_1_/*a*	\sqrt {2}a \times \sqrt{2}a \times c	M_3_^+^, M_5_^+^	Γ_5_^+^	*a* ^−^ *a* ^−^ *c*	0.44, 0.44	nRP	Papavassiliou *et al.* (2000 ▸)
200737	(*RS*)-1-Phenyl­ethyl­amine		[C_8_H_12_N]_2_PbI_4_	*P*2_1_/*a*	\sqrt {2}a \times \sqrt{2}a \times c	M_3_^+^, M_5_^+^	Γ_5_^+^	*a* ^−^ *a* ^−^ *c*	0.18, 0.18	nDJ	Billing (2002 ▸)
2016667	4-Meth­oxy-*N*-methyl­aniline		[C_8_H_12_ON]_2_PbI_4_	*P*2_1_/*a*	\sqrt {2}a \times \sqrt{2}a \times c	M_3_^+^, M_5_^+^	Γ_5_^+^	*a* ^−^ *a* ^−^ *c*	0.50, 0.50	RP	Jang & Kaminsky (2020*b* ▸)
805430	Octyl­amine		[C_8_H_20_N]_2_PbI_4_ 173 K	*P*2_1_/*a*	\sqrt {2}a \times \sqrt{2}a \times c	M_3_^+^, M_5_^+^	Γ_5_^+^	*a* ^−^ *a* ^−^ *c*	0.24, 0.24	nDJ	Lemmerer & Billing (2012*b* ▸)
805433	Nonyl­amine		[C_9_H_22_N]_2_PbI_4_ 223 K	*P*2_1_/*a*	\sqrt {2}a \times \sqrt{2}a \times c	M_3_^+^, M_5_^+^	Γ_5_^+^	*a* ^−^ *a* ^−^ *c*	0.25, 0.25	nDJ/nRP	Lemmerer & Billing (2012*b* ▸)
805435	Decyl­amine		[C_10_H_24_N]_2_PbI_4_ 243 K	*P*2_1_/*a*	\sqrt {2}a \times \sqrt{2}a \times c	M_3_^+^, M_5_^+^	Γ_5_^+^	*a* ^−^ *a* ^−^ *c*	0.11, 0.11	nDJ	Lemmerer & Billing (2012*b* ▸)
692952	Do­decyl­amine		[C_12_H_28_N]_2_PbI_4_ 319 K	*P*2_1_/*a*	\sqrt {2}a \times \sqrt{2}a \times c	M_3_^+^, M_5_^+^	Γ_5_^+^	*a* ^−^ *a* ^−^ *c*	0.14, 0.14	nDJ	Billing & Lemmerer (2008 ▸)
692954	Tetra­decyl­amine		[C_14_H_32_N]_2_PbI_4_ 335 K	*P*2_1_/*a*	\sqrt {2}a \times \sqrt{2}a \times c	M_3_^+^, M_5_^+^	Γ_5_^+^	*a* ^−^ *a* ^−^ *c*	0.13, 0.13	nDJ	Billing & Lemmerer (2008 ▸)
692956	Hexa­decyl­amine		[C_16_H_36_N]_2_PbI_4_ 341 K	*P*2_1_/*a*	\sqrt {2}a \times \sqrt{2}a \times c	M_3_^+^, M_5_^+^	Γ_5_^+^	*a* ^−^ *a* ^−^ *c*	0.11, 0.11	nDJ	Billing & Lemmerer (2008 ▸)
692958	Octa­decyl­amine		[C_18_H_40_N]_2_PbI_4_ 348 K	*P*2_1_/*a*	\sqrt {2}a \times \sqrt{2}a \times c	M_3_^+^, M_5_^+^	Γ_5_^+^	*a* ^−^ *a* ^−^ *c*	0.10, 0.10	nDJ	Billing & Lemmerer (2008 ▸)
746126	2-Iodo­ethyl­amine		[C_2_H_7_IN]_2_PbI_4_	*P*2_1_/*c*	c \times \sqrt {2}a \times \sqrt{2}a	M_3_^+^, M_5_^+^	Γ_5_^+^	*a* ^−^ *a* ^−^ *c*	0.20, 0.20	nDJ	Lemmerer & Billing (2010 ▸)
1855044	Ethyl­amine		[C_2_H_8_N]_2_PbBr_4_	*P*2_1_/*c*	c \times \sqrt {2}a \times \sqrt{2}a	M_3_^+^, M_5_^+^	Γ_5_^+^	*a* ^−^ *a* ^−^ *c*	0.46, 0.46	nRP	Luo, Guo, Li *et al.* (2019 ▸)
746124	2-Hy­droxy­ethyl­amine		[C_2_H_8_ON]_2_PbI_4_ 173 K	*P*2_1_/*c*	c \times \sqrt {2}a \times \sqrt{2}a	M_3_^+^, M_5_^+^	Γ_5_^+^	*a* ^−^ *a* ^−^ *c*	0.21, 0.21	nDJ	Lemmerer & Billing (2010 ▸)
708566	Cyclo­propyl­amine		[C_3_H_8_N]_2_PbCl_4_	*P*2_1_/*c*	c \times \sqrt {2}a \times \sqrt{2}a	M_3_^+^, M_5_^+^	Γ_5_^+^	*a* ^−^ *a* ^−^ *c*	0.50, 0.50	RP	Billing & Lemmerer (2009 ▸)
708560	Cyclo­propyl­amine		[C_3_H_8_N]_2_PbBr_4_	*P*2_1_/*c*	c \times \sqrt {2}a \times \sqrt{2}a	M_3_^+^, M_5_^+^	Γ_5_^+^	*a* ^−^ *a* ^−^ *c*	0.46, 0.46	nRP	Billing & Lemmerer (2009 ▸)
609992	Cyclo­propyl­amine		[C_3_H_8_N]_2_PbI_4_	*P*2_1_/*c*	c \times \sqrt {2}a \times \sqrt{2}a	M_3_^+^, M_5_^+^	Γ_5_^+^	*a* ^−^ *a* ^−^ *c*	0.49, 0.49	nRP	Billing & Lemmerer (2007*a* ▸)
746127	3-Iodo­propyl­amine		[C_3_H_9_IN]_2_PbI_4_	*P*2_1_/*c*	c \times \sqrt {2}a \times \sqrt{2}a	M_3_^+^, M_5_^+^	Γ_5_^+^	*a* ^−^ *a* ^−^ *c*	0.27, 0.27	nRP	Lemmerer & Billing (2010 ▸)
746125	3-Hy­droxy­propyl­amine		[C_3_H_10_ON]_2_PbI_4_	*P*2_1_/*c*	c \times \sqrt {2}a \times \sqrt{2}a	M_3_^+^, M_5_^+^	Γ_5_^+^	*a* ^−^ *a* ^−^ *c*	0.06, 0.06	nDJ	Lemmerer & Billing (2010 ▸)
955776	3-Butyn-1-amine		[C_4_H_8_N]_2_PbBr_4_	*P*2_1_/*c*	c \times \sqrt {2}a \times \sqrt{2}a	M_3_^+^, M_5_^+^	Γ_5_^+^	*a* ^−^ *a* ^−^ *c*	0.09, 0.09	nDJ	Solis-Ibarra & Karunadasa (2014 ▸)
955778	But-3-en-1-amine		[C_4_H_8_I_2_N]_2_PbBr_4_	*P*2_1_/*c*	c \times \sqrt {2}a \times \sqrt{2}a	M_3_^+^, M_5_^+^	Γ_5_^+^	*a* ^−^ *a* ^−^ *c*	0.18, 0.18	nDJ	Solis-Ibarra & Karunadasa (2014 ▸)
708567	Cyclo­butyl­amine		[C_4_H_10_N]_2_PbCl_4_	*P*2_1_/*c*	c \times \sqrt {2}a \times \sqrt{2}a	M_3_^+^, M_5_^+^	Γ_5_^+^	*a* ^−^ *a* ^−^ *c*	0.47, 0.47	nRP	Billing & Lemmerer (2009 ▸)
708561	Cyclo­butyl­amine		[C_4_H_10_N]_2_PbBr_4_	*P*2_1_/*c*	c \times \sqrt {2}a \times \sqrt{2}a	M_3_^+^, M_5_^+^	Γ_5_^+^	*a* ^−^ *a* ^−^ *c*	0.50, 0.50	RP	Billing & Lemmerer (2009 ▸)
609993	Cyclo­butyl­amine		[C_4_H_10_N]_2_PbI_4_	*P*2_1_/*c*	c \times \sqrt {2}a \times \sqrt{2}a	M_3_^+^, M_5_^+^	Γ_5_^+^	*a* ^−^ *a* ^−^ *c*	0.45, 0.45	nRP	Billing & Lemmerer (2007*a* ▸)
746128	4-Iodo­butyl­amine		[C_4_H_11_IN]_2_PbI_4_	*P*2_1_/*c*	c \times \sqrt {2}a \times \sqrt{2}a	M_3_^+^, M_5_^+^	Γ_5_^+^	*a* ^−^ *a* ^−^ *c*	0.06, 0.06	nDJ	Lemmerer & Billing (2010 ▸)
1417496	Iso­butyl­amine		[C_4_H_12_N]_2_PbBr_4_	*P*2_1_/*c*	c \times \sqrt {2}a \times \sqrt{2}a	M_3_^+^, M_5_^+^	Γ_5_^+^	*a* ^−^ *a* ^−^ *c*	0.46, 0.46	nRP	Wang *et al.* (2015 ▸)
1876240	Iso­butyl­amine		[C_4_H_12_N]_2_PbI_4_	*P*2_1_/*c*	c \times \sqrt {2}a \times \sqrt{2}a	M_3_^+^, M_5_^+^	Γ_5_^+^	*a* ^−^ *a* ^−^ *c*	0.49, 0.49	nRP	Oswald *et al.* (2018 ▸)
1495869	3-Bromo­pyridine		[C_5_H_5_BrN]_2_PbI_4_	*P*2_1_/*c*	c \times \sqrt {2}a \times \sqrt{2}a	M_3_^+^, M_5_^+^	Γ_5_^+^	*a* ^−^ *a* ^−^ *c*	0.22, 0.22	nDJ	Gómez *et al.* (2016 ▸)
708562	Cyclo­pentyl­amine		[C_5_H_12_N]_2_PbBr_4_	*P*2_1_/*c*	c \times \sqrt {2}a \times \sqrt{2}a	M_3_^+^, M_5_^+^	Γ_5_^+^	*a* ^−^ *a* ^−^ *c*	0.43, 0.43	nRP	Billing & Lemmerer (2009 ▸)
609994	Cyclo­pentyl­amine		[C_5_H_12_N]_2_PbI_4_	*P*2_1_/*c*	c \times \sqrt {2}a \times \sqrt{2}a	M_3_^+^, M_5_^+^	Γ_5_^+^	*a* ^−^ *a* ^−^ *c*	0.50, 0.50	RP	Billing & Lemmerer (2007*a* ▸)
746129	5-Iodo­pentyl­amine		[C_5_H_13_IN]_2_PbI_4_	*P*2_1_/*c*	c \times \sqrt {2}a \times \sqrt{2}a	M_3_^+^, M_5_^+^	Γ_5_^+^	*a* ^−^ *a* ^−^ *c*	0.00, 0.00	DJ	Lemmerer & Billing (2010 ▸)
1863836	2-Amino­pentane		[C_5_H_14_N]_2_PbI_4_	*P*2_1_/*c*	c \times \sqrt {2}a \times \sqrt{2}a	M_3_^+^, M_5_^+^	Γ_5_^+^	*a* ^−^ *a* ^−^ *c*	0.49, 0.49	nRP	Li, Dunlap-Shohl *et al.* (2019 ▸)
2018083	3-Methyl­butyl-1-amine		[C_5_H_14_N]_2_PbI_4_	*P*2_1_/*c*	c \times \sqrt {2}a \times \sqrt{2}a	M_3_^+^, M_5_^+^	Γ_5_^+^	*a* ^−^ *a* ^−^ *c*	0.50, 0.50	RP	Hoffman *et al.* (2020 ▸)
249243	4-Chloro­aniline		[C_6_H_7_ClN]_2_PbI_4_	*P*2_1_/*c*	c \times \sqrt {2}a \times \sqrt{2}a	M_3_^+^, M_5_^+^	Γ_5_^+^	*a* ^−^ *a* ^−^ *c*	0.30, 0.30	nRP	Liu *et al.* (2004 ▸)
723486	4-Bromo­aniline		[C_6_H_7_BrN]_2_PbI_4_	*P*2_1_/*c*	c \times \sqrt {2}a \times \sqrt{2}a	M_3_^+^, M_5_^+^	Γ_5_^+^	*a* ^−^ *a* ^−^ *c*	0.30, 0.30	nRP	Dai *et al.* (2009 ▸)
1939732	6-Amino­hexanoic acid		[C_6_H_14_O_2_N]_2_PbBr_4_	*P*2_1_/*c*	c \times \sqrt {2}a \times \sqrt{2}a	M_3_^+^, M_5_^+^	Γ_5_^+^	*a* ^−^ *a* ^−^ *c*	0.39, 0.39	nRP	Li (2020*b* ▸)
150502	1,6-Di­amino­hexane	[A]	[C_6_H_18_N_2_]PbBr_4_ RT	*P*2_1_/*c*	c \times \sqrt {2}a \times \sqrt{2}a	M_3_^+^, M_5_^+^	Γ_5_^+^	*a* ^−^ *a* ^−^ *c*	0.39, 0.39	nRP	Mousdis *et al.* (2000 ▸)
150501	1,6-Di­amino­hexane	[A]	[C_6_H_18_N_2_]PbI_4_	*P*2_1_/*c*	c \times \sqrt {2}a \times \sqrt{2}a	M_3_^+^, M_5_^+^	Γ_5_^+^	*a* ^−^ *a* ^−^ *c*	0.38, 0.38	nRP	Mousdis *et al.* (2000 ▸)
1944741	3-Fluoro­benzyl­amine		[C_7_H_9_FN]_2_PbCl_4_ 300 K	*P*2_1_/*c*	c \times \sqrt {2}a \times \sqrt{2}a	M_3_^+^, M_5_^+^	Γ_5_^+^	*a* ^−^ *a* ^−^ *c*	0.15, 0.15	nDJ	Shi *et al.* (2019 ▸)
1950233	4-Iodo­benzyl­amine		[C_7_H_9_IN]_2_PbI_4_	*P*2_1_/*c*	c \times \sqrt {2}a \times \sqrt{2}a	M_3_^+^, M_5_^+^	Γ_5_^+^	*a* ^−^ *a* ^−^ *c*	0.45, 0.45	nRP	Tremblay *et al.* (2019 ▸)
1482271	(Cyclo­hexyl­methyl)­amine		[C_7_H_16_N]_2_PbBr_4_ RT	*P*2_1_/*c*	c \times \sqrt {2}a \times \sqrt{2}a	M_3_^+^, M_5_^+^	Γ_5_^+^	*a* ^−^ *a* ^−^ *c*	0.33, 0.33	nRP	Li *et al.* (2017 ▸)
1863839	2-Amino­heptane		[C_7_H_18_N]_2_PbI_4_	*P*2_1_/*c*	c \times \sqrt {2}a \times \sqrt{2}a	M_3_^+^, M_5_^+^	Γ_5_^+^	*a* ^−^ *a* ^−^ *c*	0.47, 0.47	nRP	Li, Dunlap-Shohl *et al.* (2019 ▸)
1986789	2-(3,5-Di­chloro­phenyl)­ethyl-1-amine		[C_8_H_10_Cl_2_N]_2_PbI_4_	*P*2_1_/*c*	c \times \sqrt {2}a \times \sqrt{2}a	M_3_^+^, M_5_^+^	Γ_5_^+^	*a* ^−^ *a* ^−^ *c*	0.36, 0.36	nRP	Tremblay *et al.* (2020 ▸)
1986786	2-(3,5-Di­bromo­phenyl)­ethyl-1-amine		[C_8_H_10_Br_2_N]_2_PbI_4_	*P*2_1_/*c*	c \times \sqrt {2}a \times \sqrt{2}a	M_3_^+^, M_5_^+^	Γ_5_^+^	*a* ^−^ *a* ^−^ *c*	0.19, 0.19	nDJ	Tremblay *et al.* (2020 ▸)
1488195	4-Fluoro­phenethyl­amine		[C_8_H_11_FN]_2_PbI_4_	*P*2_1_/*c*	c \times \sqrt {2}a \times \sqrt{2}a	M_3_^+^, M_5_^+^	Γ_5_^+^	*a* ^−^ *a* ^−^ *c*	0.32, 0.32	nRP	Slavney *et al.* (2017 ▸)
1885084	(*RS*)-1-(4-Chloro­phenyl)­ethyl­amine		[C_8_H_11_ClN]_2_PbI_4_	*P*2_1_/*c*	c \times \sqrt {2}a \times \sqrt{2}a	M_3_^+^, M_5_^+^	Γ_5_^+^	*a* ^−^ *a* ^−^ *c*	0.22,0.22	nDJ	Yang *et al.* (2019 ▸)
1863838	2-Ethyl­hexyl­amine		[C_8_H_20_N]_2_PbI_4_	*P*2_1_/*c*	c \times \sqrt {2}a \times \sqrt{2}a	M_3_^+^, M_5_^+^	Γ_5_^+^	*a* ^−^ *a* ^−^ *c*	0.20, 0.20	nDJ	Li, Dunlap-Shohl *et al.* (2019 ▸)
781210	1,4-Bis(amino­methyl)­cyclo­hexane	[A]	[C_8_H_20_N_2_]PbBr_4_	*P*2_1_/*c*	c \times \sqrt {2}a \times \sqrt{2}a	M_3_^+^, M_5_^+^	Γ_5_^+^	*a* ^−^ *a* ^−^ *c*	0.15, 0.15	nDJ	Rayner & Billing (2010*a* ▸)
781212	1,4-Bis(amino­methyl)­cyclo­hexane	[A]	[C_8_H_20_N_2_]PbI_4_	*P*2_1_/*c*	c \times \sqrt {2}a \times \sqrt{2}a	M_3_^+^, M_5_^+^	Γ_5_^+^	*a* ^−^ *a* ^−^ *c*	0.14, 0.14	nDJ	Rayner & Billing (2010*b* ▸)
1521059	1,8-Di­amino­octane	[A]	[C_8_H_22_N_2_]PbBr_4_	*P*2_1_/*c*	c \times \sqrt {2}a \times \sqrt{2}a	M_3_^+^, M_5_^+^	Γ_5_^+^	*a* ^−^ *a* ^−^ *c*	0.41, 0.41	nRP	Smith *et al.* (2017 ▸)
853209	1,8-Di­amino­octane	[A]	[C_8_H_22_N_2_]PbI_4_	*P*2_1_/*c*	c \times \sqrt {2}a \times \sqrt{2}a	M_3_^+^, M_5_^+^	Γ_5_^+^	*a* ^−^ *a* ^−^ *c*	0.44, 0.44	nRP	Lemmerer & Billing (2012*a* ▸)
853212	1,5-Di­amino­naphthalene	[A]	[C_10_H_12_N­_2_]PbI_4_	*P*2_1_/*c*	c \times \sqrt {2}a \times \sqrt{2}a	M_3_^+^, M_5_^+^	Γ_5_^+^	*a* ^−^ *a* ^−^ *c*	0.01, 0.01	nDJ	Lemmerer & Billing (2012*a* ▸)
1986788	2-(3,5-Di­methyl­phenyl)­ethyl-1-amine		[C_10_H_16_N]_2_PbI_4_	*P*2_1_/*c*	c \times \sqrt {2}a \times \sqrt{2}a	M_3_^+^, M_5_^+^	Γ_5_^+^	*a* ^−^ *a* ^−^ *c*	0.31, 0.31	nRP	Tremblay *et al.* (2020 ▸)
853210	1,10-Di­amino­decane	[A]	[C_10_H_26_N_2_]PbBr_4_	*P*2_1_/*c*	c \times \sqrt {2}a \times \sqrt{2}a	M_3_^+^, M_5_^+^	Γ_5_^+^	*a* ^−^ *a* ^−^ *c*	0.20, 0.20	nDJ	Lemmerer & Billing (2012*a* ▸)
2015614	(*RS*)-1-(Naphthalen-1-yl)ethan-1-aminium		[C_12_H_14_N]_2_PbBr_4_	*P*2_1_/*c*	c \times \sqrt {2}a \times \sqrt{2}a	M_3_^+^, M_5_^+^	Γ_5_^+^	*a* ^−^ *a* ^−^ *c*	0.01, 0.01	nDJ	Jana *et al.* (2020 ▸)
853211	1,12-Di­amino­dodecane	[A]	[C_12_H_30_N_2_]PbI_4_	*P*2_1_/*c*	c \times \sqrt {2}a \times \sqrt{2}a	M_3_^+^, M_5_^+^	Γ_5_^+^	*a* ^−^ *a* ^−^ *c*	0.01, 0.01	nDJ	Lemmerer & Billing (2012*a* ▸)
1840803	Naphthalene-*O*-propyl­amine		[C­_13_H_16_ON]_2_PbI_4_	*P*2_1_/*c*	c \times \sqrt {2}a \times \sqrt{2}a	M_3_^+^, M_5_^+^	Γ_5_^+^	*a* ^−^ *a* ^−^ *c*	0.26, 0.26	nRP	Passarelli *et al.* (2018 ▸)
1876191	1-(4-Amino­butyl)­pyrene		[C_20_H_20_N]_2_PbI_4_	*P*2_1_/*c*	c \times \sqrt {2}a \times \sqrt{2}a	M_3_^+^, M_5_^+^	Γ_5_^+^	*a* ^−^ *a* ^−^ *c*	0.13, 0.13	nDJ	Van Gompel *et al.* (2019 ▸)
1840807	Perylene-*O*-ethyl­amine		[C_22_H_18_ON]_2_PbI_4_	*P*2_1_/*c*	c \times \sqrt {2}a \times \sqrt{2}a	M_3_^+^, M_5_^+^	Γ_5_^+^	*a* ^−^ *a* ^−^ *c*	0.23, 0.23	nDJ	Passarelli *et al.* (2018 ▸)
1432453	4-Chloro­benzyl­amine		[C_7_H_9_ClN]_2_PbI_4_	*P*2_1_	\sqrt {2}a \times \sqrt{2}a \times c	M_3_^+^, M_5_^+^	Γ_5_^+^	*a* ^−^ *a* ^−^ *c*	0.18, 0.18	nDJ	Weber *et al.* (2015 ▸)
1947898	4-Bromo­benzyl­amine		[C_7_H_9_BrN]_2_PbI_4_	*P*2_1_	\sqrt {2}a \times \sqrt{2}a \times c	M_3_^+^, M_5_^+^	Γ_5_^+^	*a* ^−^ *a* ^−^ *c*	0.18, 0.18	nDJ	Schmitt *et al.* (2020 ▸)
2000017	2-Bromo­phenethyl­amine		[C_8_H_11_BrN]_2_PbI_4_	*P*2_1_	\sqrt {2}a \times \sqrt{2}a \times c	M_3_^+^, M_5_^+^	Γ_5_^+^	*a* ^−^ *a* ^−^ *c*	0.17, 0.17	nDJ	Straus *et al.* (2020 ▸)
956553	(R)-1-Cyclo­hexyl­ethyl­amine		[C_8_H_18_N]_2_PbCl_4_	*P*2_1_	\sqrt {2}a \times \sqrt{2}a \times c	M_3_^+^, M_5_^+^	Γ_5_^+^	*a* ^−^ *a* ^−^ *c*	0.41, 0.41	nRP	Lemmerer & Billing (2013 ▸)
956554	(S)-1-Cyclo­hexyl­ethyl­amine		[C_8_H_18_N]_2_PbCl_4_	*P*2_1_	\sqrt {2}a \times \sqrt{2}a \times c	M_3_^+^, M_5_^+^	Γ_5_^+^	*a* ^−^ *a* ^−^ *c*	0.37, 0.37	nRP	Lemmerer & Billing (2013 ▸)
2015620	(R)-1-(Naphthalen-1-yl)ethan-1-aminium		[C_12_H_14_N]_2_PbBr_4_	*P*2_1_	\sqrt {2}a \times \sqrt{2}a \times c	M_3_^+^, M_5_^+^	Γ_5_^+^	*a* ^−^ *a* ^−^ *c*	0.15, 0.15	nDJ	Jana *et al.* (2020 ▸)
2015618	(S)-1-(Naphthalen-1-yl)ethan-1-aminium		[C_12_H_14_N]_2_PbBr_4_	*P*2_1_	\sqrt {2}a \times \sqrt{2}a \times c	M_3_^+^, M_5_^+^	Γ_5_^+^	*a* ^−^ *a* ^−^ *c*	0.12, 0.12	nDJ	Jana *et al.* (2020 ▸)
1992691	4,4-Di­fluoro­hexa­hydro­azepine		[C_6_H_12_F_2_N]_2_PbI_4_ 293 K	*P*2_1_	c \times \sqrt {2}a \times \sqrt{2}a	M_3_^+^, M_5_^+^	Γ_5_^+^	*a* ^−^ *a* ^−^ *c*	0.41, 0.41	nRP	Chen, Song, Zhang, Zhang *et al.* (2020 ▸)
955777	3,4-Di­iodo­but-3-en-1-amine		[C_4_H_8_I_2_N]_2_PbBr_4_	*P* 1	\sqrt {2}a \times \sqrt{2}a \times c	M_3_^+^, M_5_^+^	Γ_5_^+^	*a* ^−^ *a* ^−^ *c*		nDJ	Solis-Ibarra & Karunadasa (2014 ▸)
1048947	3,4-Di­bromo­butan-1-amine		[C_4_H_10_Br_2_N]_2_PbBr_4_	*P* 1	\sqrt {2}a \times \sqrt{2}a \times c	M_3_^+^, M_5_^+^	Γ_5_^+^	*a* ^−^ *a* ^−^ *c*		nDJ2	Solis-Ibarra *et al.* (2015 ▸)
1048945	3,4-Di­chloro­butan-1-amine		[C_4_H_10_Cl_2_N]_2_PbBr_4_	*P* 1	\sqrt {2}a \times \sqrt{2}a \times c	M_3_^+^, M_5_^+^	Γ_5_^+^	*a* ^−^ *a* ^−^ *c*		nDJ2	Solis-Ibarra *et al.* (2015 ▸)
853206	1,4-Di­amino­butane	[A]	[C_4_H_14_N_2_]PbBr_4_	*P* 1	\sqrt {2}a \times \sqrt{2}a \times c	M_3_^+^, M_5_^+^	Γ_5_^+^	*a* ^−^ *a* ^−^ *c*		nRP	Lemmerer & Billing (2012*a* ▸)
1053651	1,4-Di­amino­butane	[A]	[C_4_H_14_N_2_]PbI_4_	*P* 1	\sqrt {2}a \times \sqrt{2}a \times c	M_3_^+^, M_5_^+^	Γ_5_^+^	*a* ^−^ *a* ^−^ *c*		nDJ	Xiong *et al.* (2015 ▸)
1999300	5-Amino­pentanoic acid		[C_5_H_12_O_2_N]_2_PbCl_4_	*P* 1	\sqrt {2}a \times \sqrt{2}a \times c	M_3_^+^, M_5_^+^	Γ_5_^+^	*a* ^−^ *a* ^−^ *c*		nRP	Krummer (2020 ▸)
1939731	5-Amino­pentanoic acid		[C_5_H_12_O_2_N]_2_PbBr_4_	*P* 1	\sqrt {2}a \times \sqrt{2}a \times c	M_3_^+^, M_5_^+^	Γ_5_^+^	*a* ^−^ *a* ^−^ *c*		nDJ2	Li (2020*a* ▸)
1893383	2-Fluoro­phenethyl­amine		[C_8_H_11_FN]_2_PbI_4_	*P* 1	\sqrt {2}a \times \sqrt{2}a \times c	M_3_^+^, M_5_^+^	Γ_5_^+^	*a* ^−^ *a* ^−^ *c*		nDJ2	Hu *et al.* (2019*a* ▸)
1515121	2-Phenethyl­amine		[C_8_H_12_N]_2_PbI_4_	*P* 1	\sqrt {2}a \times \sqrt{2}a \times c	M_3_^+^, M_5_^+^	Γ_5_^+^	*a* ^−^ *a* ^−^ *c*		nRP	Ma *et al.* (2017 ▸)
219791	5-ammonium­ethyl­sulfanyl)-2,2′-bi­thio­phene	[A]	[C_12_H_18_S_4_N_2_]PbI_4_	*P* 1	\sqrt {2}a \times \sqrt{2}a \times c	M_3_^+^, M_5_^+^	Γ_5_^+^	*a* ^−^ *a* ^−^ *c*		nRP	Zhu *et al.* (2003 ▸)
1934875	6-[(5-Meth­oxy­naphthalen-1-yl)­oxy]hexyl-1-amine		[C_17_H_24_O_2_N]_2_PbI_4_	*P* 1	\sqrt {2}a \times \sqrt{2}a \times c	M_3_^+^, M_5_^+^	Γ_5_^+^	*a* ^−^ *a* ^−^ *c*		nDJ	Passarelli *et al.* (2020 ▸)
1885085	(R)-1-(4-Chloro­phenyl)­ethyl­amine		[C_8_H_11_ClN]_2_PbI_4_	*P*1	\sqrt {2}a \times \sqrt{2}a \times c	M_3_^+^, M_5_^+^	Γ_5_^+^	*a* ^−^ *a* ^−^ *c*		nRP	Yang *et al.* (2019 ▸)
1885086	(S)-1-(4-Chloro­phenyl)­ethyl­amine		[C_8_H_11_ClN]_2_PbI_4_	*P*1	\sqrt {2}a \times \sqrt{2}a \times c	M_3_^+^, M_5_^+^	Γ_5_^+^	*a* ^−^ *a* ^−^ *c*		nRP	Yang *et al.* (2019 ▸)
1542464	2-(2-Naphthyl)­ethyl­amine		[C_12_H_14_N]_2_PbBr_4_	*P*1	\sqrt {2}a \times \sqrt{2}a \times c	M_3_^+^, M_5_^+^	Γ_5_^+^	*a* ^−^ *a* ^−^ *c*		nRP	Du *et al.* (2017 ▸)
1883687	(2-Aza­niumyl­ethyl)­tri­methyl­phospho­nium	[A]	[C_5_H_16_PN]PbBr_4_	*P*2_1_	*a* × 2*a* × *c*	X_3_^+^	Γ_5_^+^	*a* ^+^ *b* ^0^ *c*	0.01, 0.01	nDJ	Cheng & Cao (2019 ▸)
1942547	2-(Amino­methyl)­pyridine	[A]	[C_6_H_10_N_2_]PbI_4_	*Pn*	2*a* × *c* × 2*a*	M_3_^+^, X_3_^+^	Γ_5_^+^	*a* ^+^ *b* ^+^ *c*	0.00, 0.00	DJ	Li, Ke *et al.* (2019 ▸)
295291	4-(2-amino­ethyl)­imidazole	[A]	[C_5_H_11_N_3_]PbBr_4_ RT	*P*2_1_/*c*	*c* × 2*a* × 2*a*	M_3_^+^, M_5_^+^	Γ_5_^+^	*a* ^−^ *b* ^0^ *c*		nDJ2	Li, Lin *et al.* (2007 ▸)
1915484	2-(2-amino­ethyl)­imidazole	[A]	[C_5_H_11_N_3_]PbBr_4_	*P*2_1_/*c*	*c* × 2*a* × 2*a*	M_3_^+^, M_5_^+^	Γ_5_^+^	*a* ^−^ *b* ^0^ *c*		nDJ2	Febriansyah, Giovanni *et al.* (2020 ▸)
1982716	*N* ^1^,*N* ^1^-Di­methyl­propane-1,3-di­amine	[A]	[C_5_H_16_N_2_]PbCl_4_	*P*2_1_/*c*	*c* × 2*a* × 2*a*	M_3_^+^, M_5_^+^	Γ_5_^+^	*a* ^−^ *b* ^0^ *c*		nDJ2	Jing *et al.* (2020 ▸)
1963066	*N* ^1^,*N* ^1^-Di­methyl­propane-1,3-di­amine	[A]	[C_5_H_16_N_2_]PbBr_4_	*P*2_1_/*c*	*c* × 2*a* × 2*a*	M_3_^+^, M_5_^+^	Γ_5_^+^	*a* ^−^ *b* ^0^ *c*		nDJ2	Mao *et al.* (2017 ▸)
1939809	4-(Amino­methyl)­piperidine	[A]	[C_6_H_16_N_2_]PbI_4_ 373 K	*P*2_1_/*c*	*c* × 2*a* × 2*a*	M_3_^+^, M_5_^+^	Γ_5_^+^	*a* ^0^ *a* ^0^ *c*	0, 0	DJ	Park *et al.* (2019 ▸)
1831525	4-(Amino­methyl)­piperidine	[A]	[C_6_H_16_N_2_]PbI_4_ 293 K	*Pc*	*c* × 2*a* × 2*a*	M_3_^+^, M_5_^+^	Γ_5_^+^	*a* ^0^ *a* ^0^ *c*	0, 0	DJ	Mao, Ke *et al.* (2018 ▸)
1816279	Cyclo­hexane-1,2-di­amine	[A]	[C_6_H_16_N_2_]PbI_4_	*P*2_1_2_1_2	2*a* × 2*a* × *c*	M_3_^+^		*a* ^0^ *a* ^0^ *c*	0, 0	DJ	Zhang, Wei *et al.* (2020 ▸)
1498513	2-Phenyl­ethyl­amine		[C_8_H_12_N]_2_PbCl_4_	*P* 1	2*a* × 2*a* × *c*	A_3_^+^	Γ_5_^+^	*a* ^0^ *a* ^0^ *c*/ *a* ^0^ *a* ^0^(−*c*)		nDJ2	Thirumal *et al.* (2017 ▸)
754084	2-Phenyl­ethyl­amine		[C_8_H_12_N]_2_PbBr_4_	*P* 1	2*a* × 2*a* × *c*	A_3_^+^	Γ_5_^+^	*a* ^0^ *a* ^0^ *c*/ *a* ^0^ *a* ^0^(−*c*)		nDJ2	Shibuya *et al.* (2009 ▸)
616101	2-(1-Cyclo­hexenyl)ethyl­amine		[C_8_H_16_N]_2_PbI_4_	*P* 1	2*a* × 2*a* × *c*	A_3_^+^	Γ_5_^+^	*a* ^0^ *a* ^0^ *c*/ *a* ^0^ *a* ^0^(−*c*)		nDJ2	Billing & Lemmerer (2006*a* ▸)
2011085	2-(4-Meth­oxy­phenyl)­ethyl-1-amine		[C_9_H_14_ON]_2_PbI_4_	*P* 1	2*a* × 2*a* × *c*	A_3_^+^	Γ_5_^+^	*a* ^0^ *a* ^0^ *c*/ *a* ^0^ *a* ^0^(−*c*)		nRP	Jiahui (2020 ▸)
1861843	2-([2,2′-Bi­thio­phen]-5-yl)ethyl-1-amine		[C_10_H_12_S_2_N]_2_PbI_4_	*P*1	2*a* × 2*a* × *c*					nRP	Gao *et al.* (2019 ▸)
1840804	(Naphthalene-*O*-propyl­amine^*^		[C_13_H_16_ON]_2_[C_4_H_6_O_2_]PbI_4_	*P* 1	2*a* × 2*a* × *c*	A_3_^+^, A_5_^+^				nDJ2	Passarelli *et al.* (2018 ▸)
1846391	2-(2^4^,4^3^-Di­methyl[1^2^,2^2^:2^5^,3^2^:3^5^,4^2^-quaterthio­phen]-1^5^-yl)ethyl­amine		[C_24_H_22_S_4_N]_2_PbI_4_	*P* 1	2*a* × 2*a* × *c*					nRP	Gao *et al.* (2019 ▸)
1875165	4-(Amino­methyl)­piperidine	[A]	[C_6_H_16_N_2_]PbBr_4_	*Pca*2_1_	2\sqrt {2}a \times c \times \sqrt{2}a	M_3_^+^				DJ	Mao, Guo *et al.* (2018 ▸)
1934874	6-[(Naphthalen-1-yl)­oxy]hexyl-1-amine		[C_16_H_22_ON]_2_PbI_4_	*P* 1	c \times 3\sqrt {2}a \times \sqrt{2}a	M_3_^+^, M_5_^+^				nRP	Passarelli *et al.* (2020 ▸)

**Table 5:** Summary of experimentally known structures with at least one unit-cell metric larger than 2*a*_RP_ or 2*c*_RP_ The parent structure type is that of the RP phase (*I*4/*mmm*).

						Key modes				
CCDC number	Amine	Type	Formula	Space group	Metrics	Tilt	Layer shift	Other modes	Structure type	Reference
995699	*N* ^1^,*N* ^1^-Di­methyl­propane-1,3-di­amine	[A]	[C_5_H_16_N_2_]PbI_4_	*Pbca*	2\sqrt {2}a \times \sqrt {2}a \times c	X_2_^+^	M_5_^−^	Δ_3_, Y_4_	nDJ	Yu *et al.* (2014 ▸)
702987	4-Amidino­pyridine	[A]	[C_6_H_9_N_3_]PbBr_4_	*Pbca*	2\sqrt {2}a \times \sqrt {2}a \times c	X_2_^+^	M_5_^−^	Δ_3_, Y_4_	nDJ	Li *et al.* (2008 ▸)
1838616	2-(Amino­methyl)­pyridine	[A]	[C_6_H_10_N_2_]PbCl_4_ RT	*Pbca*	2\sqrt {2}a \times \sqrt {2}a \times c	X_2_^+^	M_5_^−^	Δ_3_, Y_4_	nRP	Lermer *et al.* (2018 ▸)
666178	2-(Amino­methyl)­pyridine	[A]	[C_6_H_10_N_2_]PbBr_4_	*Pbca*	2\sqrt {2}a \times \sqrt {2}a \times c	X_2_^+^	M_5_^−^	Δ_3_, Y_4_	nRP	Li, Zheng *et al.* (2007 ▸)
1838617	2-(Amino­methyl)­pyridine	[A]	[C_6_H_10_N_2_]PbI_4_	*Pbca*	2\sqrt {2}a \times \sqrt {2}a \times c	X_2_^+^	M_5_^−^	Δ_3_, Y_4_	nDJ	Lermer *et al.* (2018 ▸)
1048275	1-(2-Amino­ethyl)­piperazine	[A]	[C_6_H_17_N_3_]PbI_4_	*Pbca*	2\sqrt {2}a \times \sqrt {2}a \times c	X_2_^+^	M_5_^−^	Δ_3_, Y_4_	nDJ	Liu *et al.* (2015 ▸)
1915485	3-(2-Amino­ethyl)­pyridine	[A]	[C_7_H_12_N_2_]PbBr_4_	*Pbca*	2\sqrt {2}a \times \sqrt {2}a \times c	X_2_^+^	M_5_^−^	Δ_3_, Y_4_	nRP	Febriansyah, Giovanni *et al.* (2020 ▸)
724584	Cystamine	[A]	[C_4_H_14_S_2_N_2_]PbI_4_	*P*2_1_/*n*	2\sqrt {2}a \times \sqrt {2}a \times c	X_2_^+^	M_5_^−^,Γ_5_^+^	Y_3_	nDJ	Louvain *et al.* (2014 ▸)
1995236	(*RS*)-2-Phenyl­propyl-1-amine		[C_9_H_14_N]_2_PbBr_4_	*Pbca*	4\sqrt {2}a \times \sqrt {2}a \times c	X_2_^+^	M_5_^−^	Δ_3_, Y_2_, Y_4_	nDJ	Trujillo-Hernández *et al.* (2020 ▸)
1831521	3-(Amino­methyl)­piperidine	[A]	[C_6_H_16_N_2_]PbI_4_	*P*2_1_/*c*	\sqrt {2}a \times 2\sqrt {2}a \times c	X_2_^+^	M_5_^−^		nDJ	Mao, Ke *et al.* (2018 ▸)
1937299	Methyl­hydrazine		[CH_7_N_2_]_2_PbI_4_ 280 K	*Pccn*	*c* × 3*a* × 2*a*	C	M_5_^−^	Σ_4_	nRP	Mączka *et al.* (2019 ▸)
1937297	Methyl­hydrazine		[CH_7_N_2_]_2_PbI_4_ 100 K	*P* 1	*c* × 3*a* × 2*a*	C	M_5_^−^	Σ_4_	nRP	Mączka *et al.* (2019 ▸)
1963065	*N* ^1^,*N* ^1^-Di­methyl­ethyl-1,2-di­amine	[A]	[C_4_H_14_N_2_]PbBr_4_	*P*2_1_/*c*	3*a* × 2*a* × *c*				nDJ	Mao *et al.* (2017 ▸)
1887281	*N* ^1^,*N* ^1^-Di­methyl­ethyl-1,2-di­amine	[A]	[C_4_H_14_N_2_]PbCl_4_	*Pbcn*	2\sqrt {2}a \times 2\sqrt {2}a \times c		M_5_^−^	Σ_1_, Σ_4_, Δ_3_, Y_4_	nDJ	Rong *et al.* (2019 ▸)
1982717	*N* ^1^,*N* ^1^-Di­methyl­ethyl-1,2-di­amine	[A]	[C_4_H_14_N_2_]PbCl_4_	*Pca*2_1_	2\sqrt {2}a \times 2\sqrt {2}a \times c	X_3_^+^	M_5_^−^	Σ_1_, Σ_4_, Δ_3_, Y_4_	nDJ	Jing *et al.* (2020 ▸)
1838611	2-(Amino­methyl)­pyridine	[A]	[C_6_H_10_N_2_]PbCl_4_ 100 K	*Pna*2_1_	c \times 2\sqrt {2}a \times 2\sqrt {2}a		M_5_^−^	Σ_1_, Σ_4_, Δ_3_, Y_3_, Y_4_	nRP	Lermer *et al.* (2018 ▸)
1521060	4-Amino­butanoic acid		[C_4_H_10_O_2_N]_2_PbBr_4_	*P*2_1_/*c*	3\sqrt {2}a \times \sqrt {2}a \times 2c				nDJ	Smith *et al.* (2017 ▸)
1963067	*N* ^1^,*N* ^1^-Di­methyl­butyl-1,4-di­amine	[A]	[C_6_H_18_N_2_]PbBr_4_	*Aba*2	2*c* × 4*a* × 2*a*	C	M_5_^−^,Λ_5_	Γ_5_^−^	nDJ2	Mao *et al.* (2017 ▸)
