# Supplementary material for: Structural chemistry of layered lead halide perovskites containing single octahedral layers
Source: IUCrJ. 2021 Jun 30;8(Pt 4):485–513. doi: 10.1107/S2052252521005418 (PMC8256700; doi:10.1107/S2052252521005418)
Supplement: Supplementary file 1 [file m-08-00485-sup1.pdf]

IUCrJ

Volume 8 (2021)

Supporting information for article:

Structural Chemistry of Layered Lead Halide Perovskites Containing Single Octahedral Layers

Jason A. McNulty and Philip Lightfoot

S1. A brief introduction to isodistort

ISODISTORT (B. J. Campbell *et al*., *J. Appl. Cryst*., 2009, **39**, 607-614) is a freely-available on-line crystallographic tool (https://stokes.byu.edu/iso/isodistort.php). It uses representational analysis to describe the distortions of an experimentally-determined crystal structure in a way that reflects more 'naturally' the types of co-operative distortion present than a conventional crystallographic description expressed in terms of individual atomic coordinates. The number of variable parameters does not change: they are simply re-cast as basis functions of irreducible representations (irreps) of the parent space group symmetry, so that co-operative displacements of groups of atoms, rather than individual atoms, can be considered. This makes it easy to compare apparently unrelated structures and to quickly identify key 'distortion modes' such as, in our case, octahedral tilting and layer displacements, which are common within a structural family. In this work, ISODISTORT has been used in two ways:

1. Primarily, to compare the cif file for an experimentally-determined crystal structure to that of an idealised 'parent' phase.
2. To determine the permitted 'highest symmetry' space groups that can be derived from simple distortion modes, or combinations of distortions, from the parent phase.

For simplicity in comparing the 'BX_4' layer structure of the present compounds, without needing to consider the organic moieties, the cif for the parent phase has been adjusted from the aristotype inorganic $n = 1$ RP phase K_2NiF_4 (or DJ phase $TlAlF_4$) by removing the K(or Tl), renaming the Ni (or Al) and F positions to Pb and X (Cl, Br or I), respectively, and adjusting the unit cell parameters and z-coordinate of the apical X atom, to produce Pb-X bond lengths that are similar in dimensions to those expected. Since the ISODISTORT software uses an approach similar to normal mode analysis, none of these adjustments affect the key derived outputs, which are the symmetry-allowed distortion modes at the relevant points in the first Brillouin zone of the parent phase, and their magnitudes.

Further details and examples of the methods are given on-line (https://stokes.byu.edu/iso/isodistorthelp.php).

S1.1. Comparison of an experimental structure to a parent phase

We took the parent phases from ICSD. For example, the cif for K_2NiF_4 (#73450) has the space group *I*4/*mmm*, unit cell parameters a = 4.013, c = 13.088 Å, and atomic coordinates Ni (0,0,0), K (0,0,0.3538), F1 (0, ½, 0), F2 (0,0,0.1533). We adjusted this to correspond approximately to the unit cell dimensions of the experimental phase, and to give Pb-X bond lengths of approximately the expected magnitude. For example, for CCDC-1938881 (Table 2), the first example of the common

Pbca structure type, the parent cif is adapted to: a = 5.6783, c = 23.704 Å, and atomic coordinates Pb (0,0,0), Br1 (0, ½, 0), Br2 (0,0,0.12). [Note that the origin setting is important: we always use Pb at the origin]. In order to compare the experimental cif, we need to delete the coordinates and atomic displacement parameters of the organic moieties. Then, read the parent cif into ISODISTORT and choose 'Method 4' to read in the experimental cif:

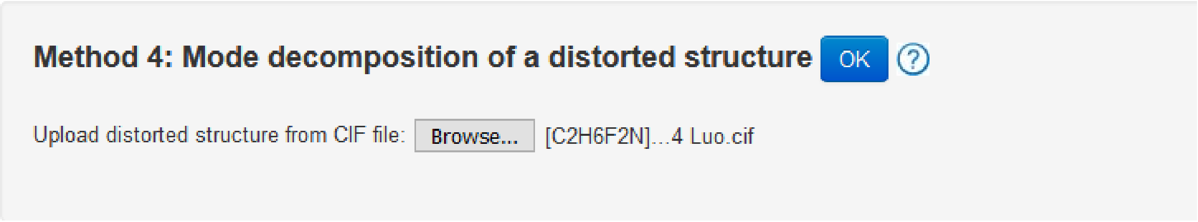

In most cases, ISODISTORT will suggest a correct basis which transforms the parent unit cell to the child:

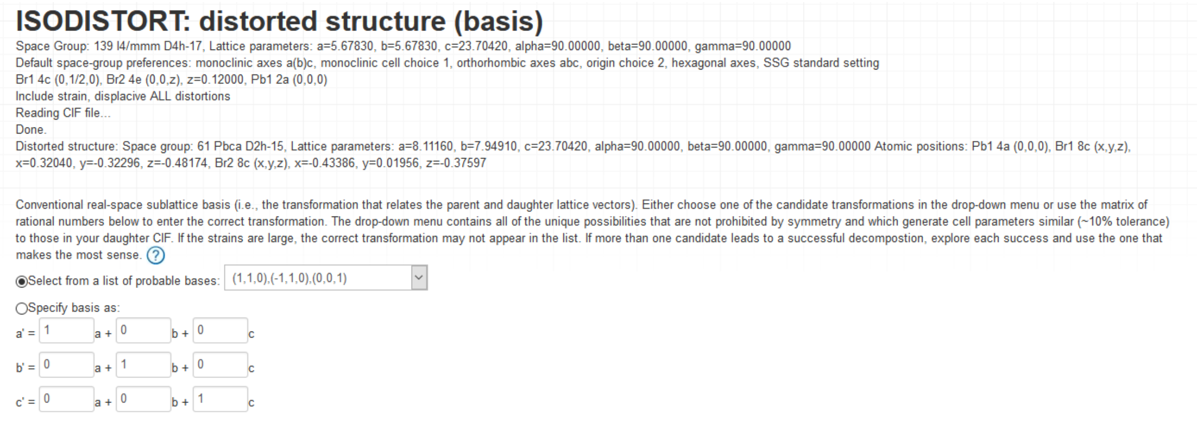

Click 'OK' and the next screen gives the derived mode amplitudes, which can then be visualised:

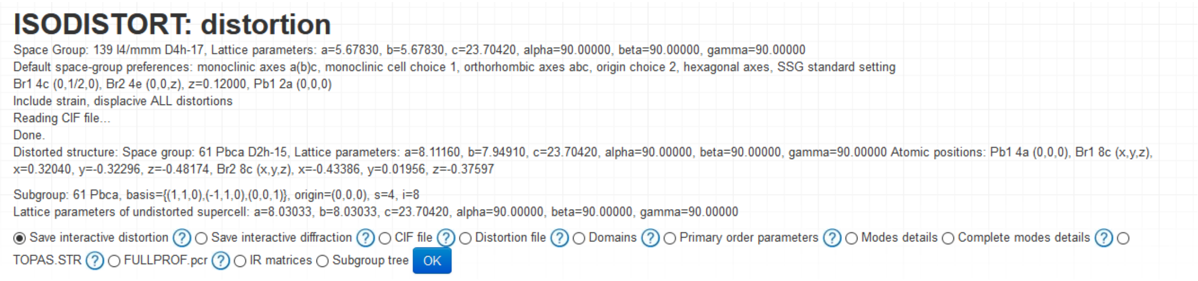

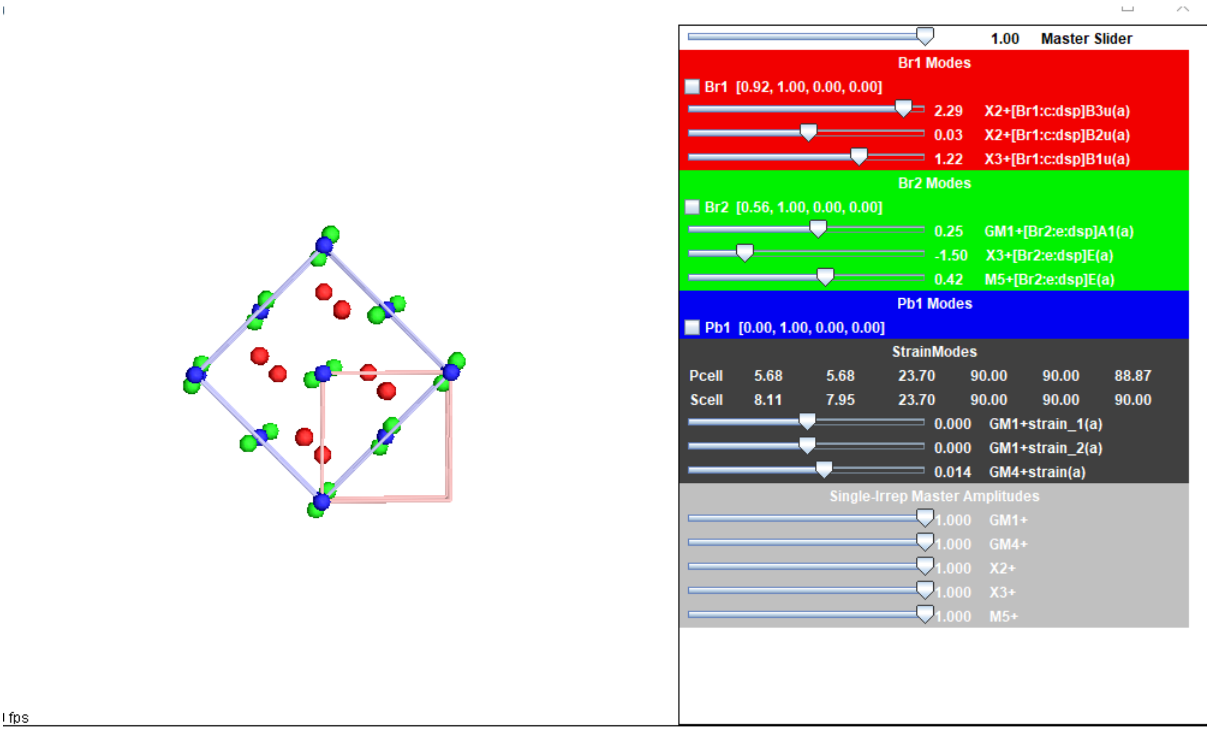

In this case, the X_2^+ (rotation) and X_3^+ (tilt) stand out clearly as the most significant modes.

Note that in many cases, structures that look quite complex (based on unit cell metrics and space group) will have many allowed distortion modes, but the genuinely significant ones are often a small subset of those allowed. An example is 1043214, below, where the P_4 and Γ_5^+ modes stand out, most of the others being close to zero activity:

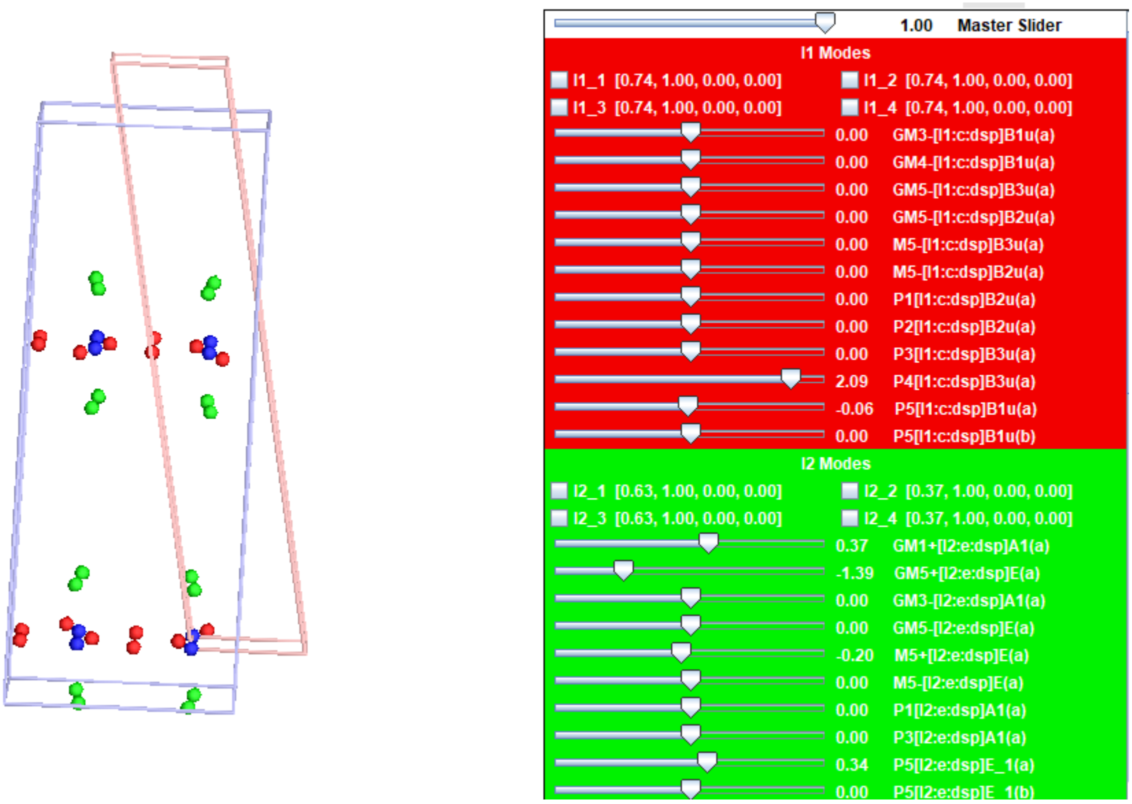

S1.2. Identifying crystallographic models from modes and mode combinations

Once the commonly-occuring distortion modes within a given family of compounds have been identified, ISODISTORT can be used to derive the simplest structural models compatible with these. For example, starting again from the RP parent phase (composition and unit cell size are now irrelevant), and considering only the single rotation mode X_2^+, we choose 'Method 2'and specify the *k*-point X (½, ½, 0):

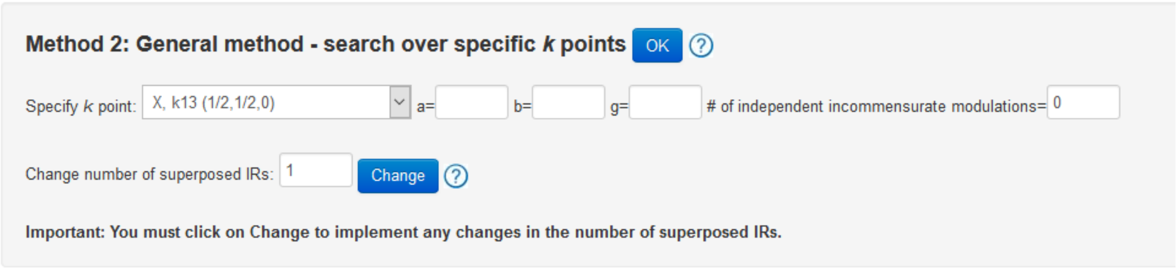

The next screen gives the options:

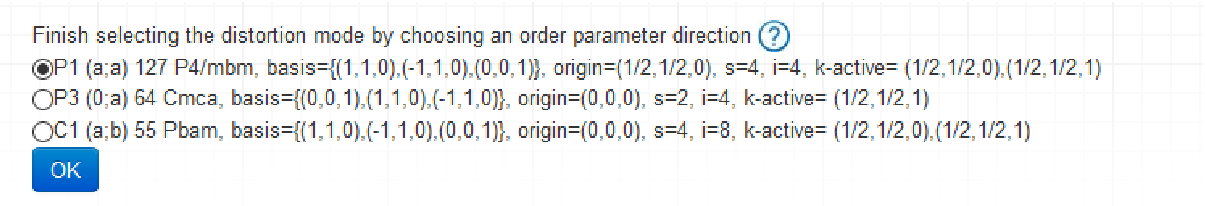

We see that there are three distinct options for this two-dimensional irrep, designated by the Order Parameter Directions (OPD) (a;a), (0;a) and (a;b). The second option, X_2^+(0;a) with unit cell metrics $c \times\sqrt{2}a \times\sqrt{2}a$, relative to the parent and space group *Cmca*, occurs quite commonly in our survey (examples commencing #2016195 in Table 2). This corresponds to the Glazer-like tilt system a^0a^0c/a^0a^0c. The other two options do not occur. All three options describe rotation of octahedra perpendicular to the layer plane. The differences are in the magnitudes of the rotation in the two layers, *viz*: equal magnitudes for (0;a); one layer rotated, one fixed for (a;a); and two different magnitudes of rotation for (a;b). Similar styles of option for OPD occur for the other simple tilt and layer-shift modes.

It is straightforward to use Method 2 to combine two or more irrep modes. For example, superposition of X_2^+ rotation and X_3^+ tilt ($X_2^+ \oplus X_3^+$) gives:

Finish selecting the distortion mode by choosing an order parameter direction ?
elapsed time:
◉ P3(1)P3(1) (0;a|0;b) 14 P2_1/c, basis={(1/2,1/2,1/2),(-1,1,0),(-1,-1,0)}, origin=(0,0,0), s=2, i=8, k-active= (1/2,1/2,1);(1/2,1/2,1)
○ P3(1)P3(2) (0;a|b;0) 61 Pbca, basis={(-1,1,0),(0,0,1),(1,1,0)}, origin=(0,0,0), s=4, i=8, k-active= (1/2,1/2,1);(1/2,1/2,0)
○ C1(1)P3(1) (a;b|0;c) 14 P2_1/c, basis={(0,0,1),(1,-1,0),(1,1,0)}, origin=(0,0,0), s=4, i=16, k-active= (1/2,1/2,0),(1/2,1/2,1);(1/2,1/2,1)
○ P1(1)P1(1) (a;a|b;b) 12 C2/m, basis={(0,2,0),(2,0,0),(0,0,-1)}, origin=(0,0,0), s=4, i=16, k-active= (1/2,1/2,0),(1/2,1/2,1);(1/2,1/2,0),(1/2,1/2,1)
○ P3(1)C1(1) (0;a|b;c) 14 P2_1/c, basis={(1,1,0),(-1,1,0),(0,0,1)}, origin=(0,0,0), s=4, i=16, k-active= (1/2,1/2,1);(1/2,1/2,0),(1/2,1/2,1)
○ C1(1)C1(1) (a;b|c;d) 2 P-1, basis={(1,1,0),(1,-1,0),(0,0,-1)}, origin=(0,0,0), s=4, i=32, k-active= (1/2,1/2,0),(1/2,1/2,1);(1/2,1/2,0),(1/2,1/2,1)
OK

Here, the OPD represents the combined contributions from X_2^+ (the first two symbols) and X_3^+ (the latter two). Here, the first option is actually an alternative route to derive the common $P2_1/c$ (a^-a^-c) system in Table 4 (for which we used the DJ parent) and the second option is the common $a^-a^-c/-(a^-a^-)c$ system in Table 2.

As a second example, X_2^+ rotation and M_5^- layer shift ($X_2^+ \oplus M_5^-$) produces:

Finish selecting the distortion mode by choosing an order parameter direction ?
elapsed time:
◉ P3(1)P1(1) (0;a|b,0) 57 Pbcm, basis={(1,1,0),(-1,1,0),(0,0,1)}, origin=(1/4,1/4,1/4), s=4, i=8, k-active= (1/2,1/2,1);(1,1,1)
○ P3(1)P1(2) (0;a|0,-b) 62 Pnma, basis={(1,1,0),(0,0,1),(1,-1,0)}, origin=(1/4,1/4,1/4), s=4, i=8, k-active= (1/2,1/2,1);(1,1,1)
○ C1(1)P1(1) (a;b|c,0) 26 Pmc2_1, basis={(0,0,1),(1,1,0),(-1,1,0)}, origin=(1/4,1/4,0), s=4, i=16, k-active= (1/2,1/2,0),(1/2,1/2,1);(1,1,1)
○ P1(1)P3(1) (a;a|b,b) 38 Amm2, basis={(0,0,1),(-2,0,0),(0,-2,0)}, origin=(0,0,0), s=4, i=16, k-active= (1/2,1/2,0),(1/2,1/2,1);(1,1,1)
○ P3(1)C1(1) (0;a|b,c) 11 P2_1/m, basis={(-1,1,0),(0,0,1),(1,1,0)}, origin=(1/4,1/4,1/4), s=4, i=16, k-active= (1/2,1/2,1);(1,1,1)
○ C1(1)C1(1) (a;b|c,d) 6 Pm, basis={(-1,1,0),(0,0,1),(1,1,0)}, origin=(0,0,0), s=4, i=32, k-active= (1/2,1/2,0),(1/2,1/2,1);(1,1,1)
OK

Several examples of the second option (*Pnma*) are seen in Table 2 (e.g. #193882) but the others do not occur. Note that not all the combinations that might be expected (e.g. (a,a|b,0) are listed, as they turn out to special cases of more general directions, e.g. (a,b|c,0). Note also that some of these combinations *do* naturally give rise to non-centrosymmetric or polar space groups (this does *not* occur for superposition of two ‘simple’ tilt or rotation modes, like $X_2^+ \oplus X_3^+$, in $n = 1$ RP or DJ phases).

Table S1 Structures of amines corresponding to those in Table 1 of the main text.

Amine	Formula	Amine	Formula
4,4-Difluoropiperidine 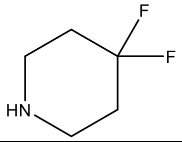	$[C_5H_{10}F_2N]_2PbI_4$	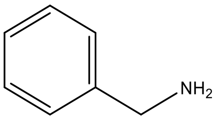 Benzylamine	$[C_7H_{10}N]_2PbCl_4$
2-Fluorobenzylamine 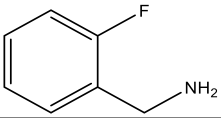	$[C_7H_9FN]_2PbCl_4$	3-Fluorobenzylamine 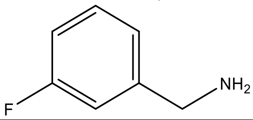	$[C_7H_9FN]_2PbCl_4$
4-Fluorobenzylamine 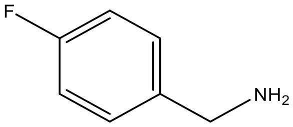	$[C_7H_9FN]_2PbCl_4$	4,4-Difluorohexahydroazepine 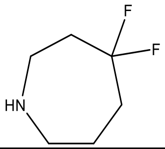	$[C_6H_{12}F_2N]_2PbI_4$
4,4-Difluorohexahydroazepine 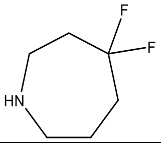	$[C_6H_{12}F_2N]PbI_4$	Methylhydrazine 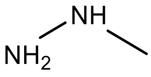	$[CH_7N_2]_2PbI_4$
1,4-Dimethylpiperazine 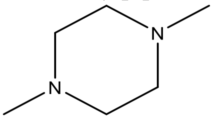	$[C_6H_{16}N_2]PbBr_4$	1-Phenylethylamine 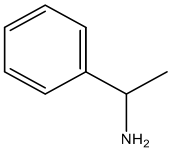	$[C_8H_{12}N]_2PbI_4$

Table S2 Structures of amines corresponding to those in Table 2 of the main text.

Amine	Formula	Amine	Formula
3-Fluoro-*N*-methylbenzylamine 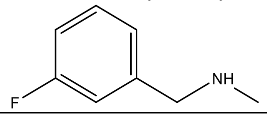	$[C_8H_{11}FN]_2PbBr_4$	2-Bromoethylamine 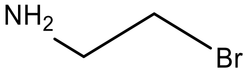	$[C_2H_7BrN]_2PbI_4$
2,2′-Biimidazole 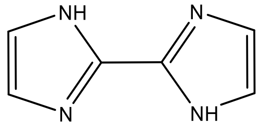	$[C_6H_8N_4]PbI_4$	Butan-2-amine 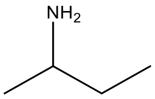	$[C_4H_{12}N]_2PbBr_4$
4,4-Difluoropiperidine 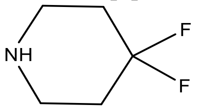	$[C_5H_{10}F_2N]PbI_4$	4,4-Difluorohexahydroazepine 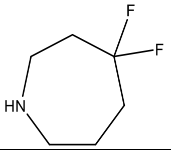	$[C_6H_{12}F_2N]PbI_4$
Butylamine 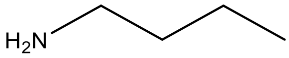	$[C_4H_{12}N]_2PbCl_4$	Isobutylamine 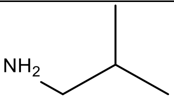	$[C_4H_{12}N]_2PbBr_4$
Cyclopentylamine 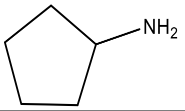	$[C_5H_{12}N]_2PbCl_4$	Cyclohexylamine 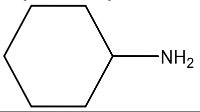	$[C_6H_{14}N]_2PbBr_4$
Cyclohexylamine 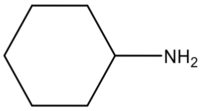	$[C_6H_{14}N]_2PbI_4$	Benzylamine 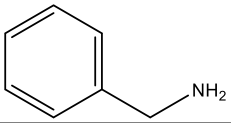	$[C_7H_{10}N]_2PbBr_4$
(Cyclohexylmethyl)amine 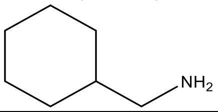	$[C_7H_{16}N]_2PbBr_4$	Octylamine 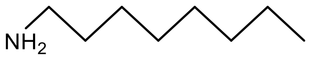	$[C_8H_{20}N]_2PbI_4$
Decylamine 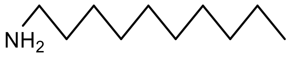	$[C_{10}H_{24}N]_2PbI_4$	Butylamine 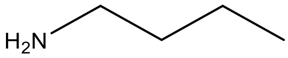	$[C_4H_{12}N]_2PbCl_4$
4-Aminotetrahydropyran 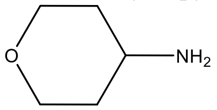	$[C_5H_{12}ON]_2PbBr_4$	4,4-Difluorocyclohexylamine 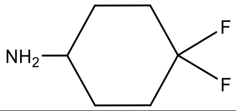	$[C_6H_{12}F_2N]_2PbI_4$
Cyclohexylamine 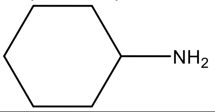	$[C_6H_{14}N]_2PbBr_4$	Hexylamine 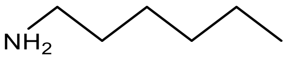	$[C_6H_{16}N]_2PbI_4$
2-Fluorobenzylamine 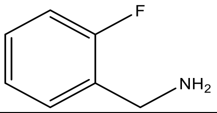	$[C_7H_9FN]_2PbCl_4$	Benzylamine 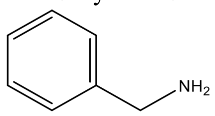	$[C_7H_{10}N]_2PbBr_4$

Benzylamine 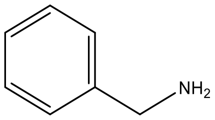	$[C_7H_{10}N]_2PbCl_4$	1-(2-Naphthyl)-methylamine 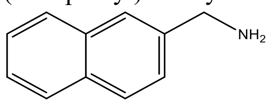	$[C_{11}H_{12}N]_2PbBr_4$
2-(4-Biphenyl)ethylamine 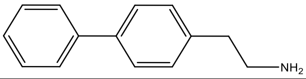	$[C_{14}H_{16}N]_2PbI_4$	2-Hydroxyethylamine 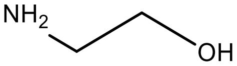	$[C_2H_8ON]_2PbBr_4$
4-Fluoro-*N*-methylaniline 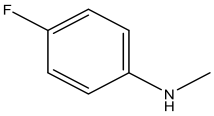	$[C_7H_9FN]_2PbI_4$	3-Fluoro-*N*-methylbenzylamine 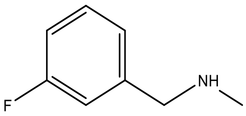	$[C_8H_{11}FN]_2PbBr_4$
3-Chloro-*N*-methylbenzylamine 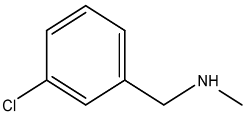	$[C_8H_{11}ClN]_2PbI_4$	3-Bromo-*N*-methylbenzylamine 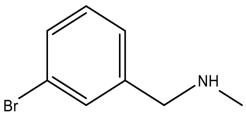	$[C_8H_{11}BrN]_2PbCl_4$
3-Bromo-*N*-methylbenzylamine 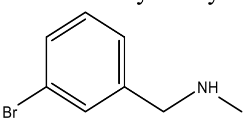	$[C_8H_{11}BrN]_2PbBr_4$	2,2,2-Trifluoroethylamine 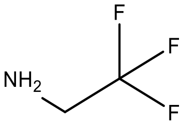	$[C_2H_5F_3N]_2PbBr_4$
2-Fluoroethylamine 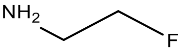	$[C_2H_7FN]_2PbCl_4$	2-Cyanoethylamine 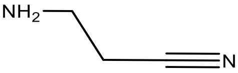	$[C_3H_7N_2]_2PbI_4$
Propylamine 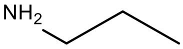	$[C_3H_{10}N]_2PbCl_4$	3-Bromopyridine 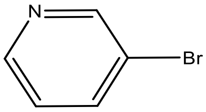	$[C_5H_5BrN]_2PbBr_4$
4-Fluorobenzylamine 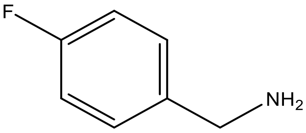	$[C_7H_9FN]_2PbCl_4$	1-Cyclohexylethylamine 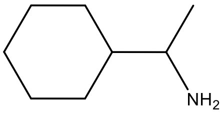	$[C_8H_{18}N]_2PbBr_4$
1,4-bis-(Aminomethyl)-cyclohexane 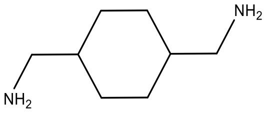	$[C_8H_{20}N_2]PbCl_4$	Ethyl-1,2-diamine 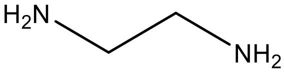	$[C_2H_{10}N_2]PbI_4$
2,2-Difluoroethylamine 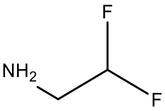	$[C_2H_6F_2N]_2PbBr_4$	4-Aminobutanoic acid 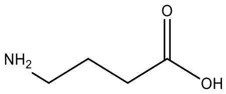	$[C_4H_{10}O_2N]_2PbI_4$
Butylamine 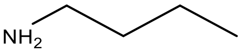	$[C_4H_{12}N]_2PbCl_4$	Butylamine 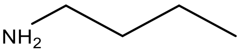	$[C_4H_{12}N]_2PbBr_4$
Butylamine 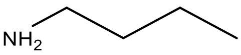	$[C_4H_{12}N]_2PbI_4$	2-Thiophenemethylamine 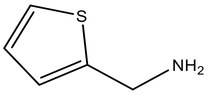	$[C_5H_8SN]_2PbCl_4$
2-Thiophenemethylamine 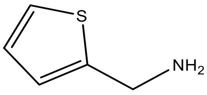	$[C_5H_8SN]_2PbBr_4$	2-Thiophenemethylamine 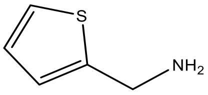	$[C_5H_8SN]_2PbI_4$

Pentylamine 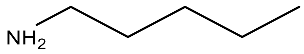	$[C_5H_{14}N]_2PbI_4$	4,4-Difluorocyclohexylamine 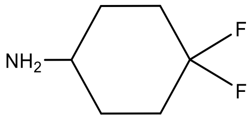	$[C_6H_{12}F_2N]_2PbI_4$
Cyclohexylamine 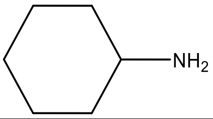	$[C_6H_{14}N]_2PbI_4$	6-Iodohexylamine 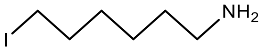	$[C_6H_{15}IN]_2PbI_4$
Hexylamine 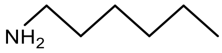	$[C_6H_{16}N]_2PbI_4$	Benzylamine 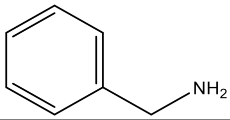	$[C_7H_{10}N]_2PbI_4$
Heptylamine 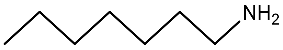	$[C_7H_{18}N]_2PbI_4$	Octylamine 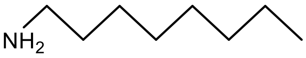	$[C_8H_{20}N]_2PbI_4$
Nonylamine 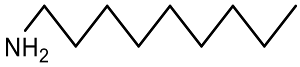	$[C_9H_{22}N]_2PbI_4$	Deca-3,5-diyn-1-amine 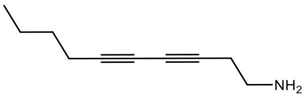	$[C_{10}H_{16}N]_2PbBr_4$
Decylamine 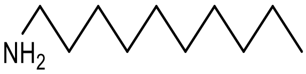	$[C_{10}H_{24}N]_2PbI_4$	Dodecylamine 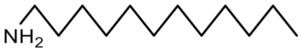	$[C_{12}H_{28}N]_2PbI_4$
2-((5-Methoxynaphthalen-1-yl)oxy)ethan-1-amine 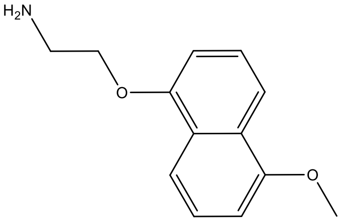	$[C_{13}H_{16}O_2N]_2PbI_4$	Tetradecylamine 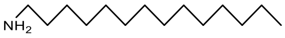	$[C_{14}H_{32}N]_2PbI_4$
Hexadecylamine 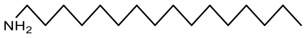	$[C_{16}H_{36}N]_2PbI_4$	Octadecylamine 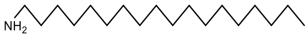	$[C_{18}H_{40}N]_2PbI_4$
2-Methylpentane-1,5-diamine 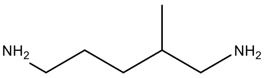	$[C_6H_{18}N_2]PbCl_4$	2-Methylpentane-1,5-diamine 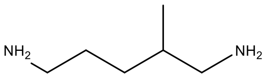	$[C_6H_{18}N_2]PbBr_4$
1,9-diaminononane 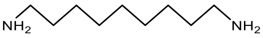	$[C_9H_{24}N_2]PbI_4$ Li	Pyrene-*O*-propylamine 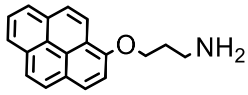	$[C_{19}H_{18}ON]_2PbI_4$
1,4-Diaminobutane 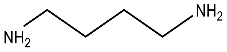	$[C_4H_{14}N_2]PbI_4$	2-Methylpentane-1,5-diamine 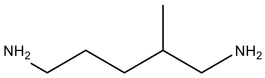	$[C_6H_{18}N_2]PbBr_4$
4-Chlorophenethylamine 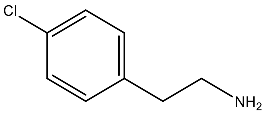	$[C_8H_{11}ClN]_2PbI_4$	4-Bromophenethylamine 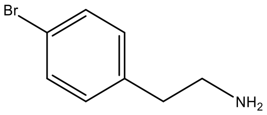	$[C_8H_{11}BrN]_2PbI_4$
(RS)-1-Cyclohexylethylamine 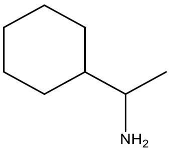	$[C_8H_{18}N]_2PbCl_4$	Hexadecylamine 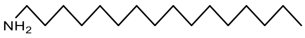	$[C_{16}H_{36}N]_2PbI_4$

4-[(Naphthalen-1-yl)oxy]butyl-1-amine 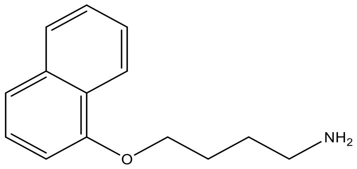	$[C_{14}H_{18}ON]_2PbI_4$	Butylamine 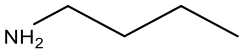	$[C_4H_{12}N]_2PbBr_4$
Propane-1,3-diamine 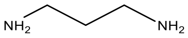	$[C_3H_{12}N_2]PbCl_4$	(R)-1-Phenylethylamine 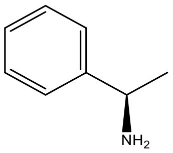	$[C_8H_{12}N]_2PbI_4$
(S)-1-Phenylethylamine 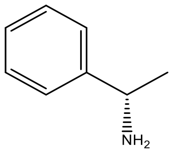	$[C_8H_{12}N]_2PbI_4$	3-(Aminoethyl)pyridine 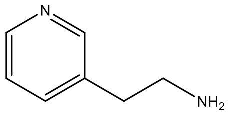	$[C_6H_{10}N_2]PbI_4$
3-Fluoro-*N*-methylaniline 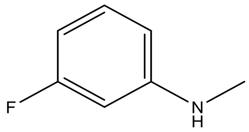	$[C_7H_9FN]_2PbI_4$	*m*-Phenylenediamine 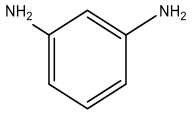	$[C_6H_{10}N_2]PbCl_4$
1,4-Diaminobutane 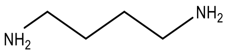	$[C_4H_{14}N_2]PbCl_4$	*N*-methylpropane-1,3-diamine 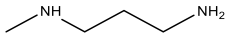	$[C_4H_{14}N_2]PbBr_4$
2,2′-Dithiobis(ethylamine) 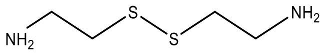	$[C_4H_{14}S_2N_2]PbCl_4$	1-Methylpiperidin-4-amine 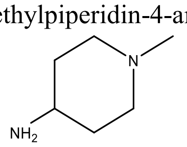	$[C_6H_{16}N_2]PbI_4$
1,6-Diaminohexane 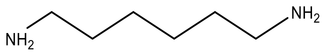	$[C_6H_{18}N_2]PbCl_4$	3-(2-Aminoethyl)aniline 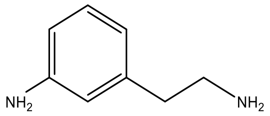	$[C_8H_{14}N_2]PbBr_4$
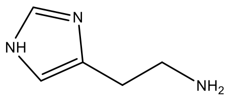 Histamine	$[C_5H_{11}N_3]PbI_4$	5-Aminopentanoic acid 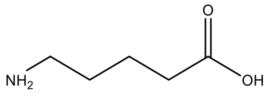	$[C_5H_{12}O_2N]_2PbBr_4$
4-Fluorobenzylamine 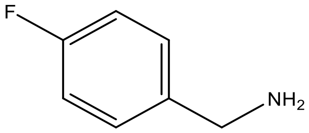	$[C_7H_9FN]_2PbI_4$	3-(Pentachlorophenoxy)propyl-1-amine 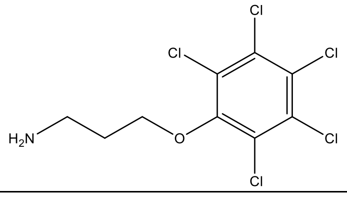	$[C_9H_9Cl_5ON]_2PbI_4$

Table S3 Structures of amines corresponding to those in Table 3 of the main text.

Amine	Formula	Amine	Formula
Piperidine 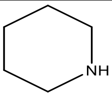	$[C_5H_{12}N]_2PbCl_4$	Benzimidazole 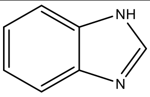	$[C_7H_7N_2]_2PbI_4$
3-Fluorophenethylamine 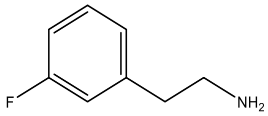	$[C_8H_{11}FN]_2PbI_4$	Pyrene-*O*-butylamine 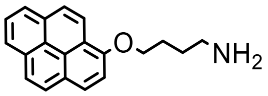	$[C_{20}H_{20}ON]_2PbI_4$
2-(3‴,4′-dimethyl-[2,2′:5′,2″:5″,2‴-quaterthiophen]-5-yl)ethan-1-amine 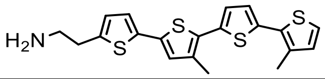	$[C_{20}H_{20}S_4N]_2PbI_4$	2-Fluoroethylamine 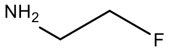	$[C_2H_7FN]_2PbBr_4$
2-Bromoethylamine 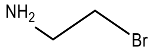	$[C_2H_7BrN]_2PbI_4$	Piperidine 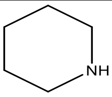	$[C_5H_{12}N]_2PbBr_4$
2-Chloroethylamine 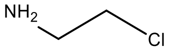	$[C_2H_7ClN]_2PbI_4$	2-Bromoethylamine 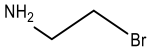	$[C_2H_7BrN]_2PbI_4$
Methylamine & Guanidine 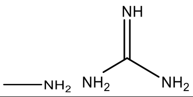	$[CH_6N][CH_6N_3]PbI_4$	Guanidine 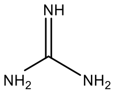	$[CH_6N_3]CsPbBr_4$
4-(2-Aminoethyl)pyridine 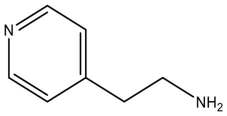	$[C_7H_{12}N_2]PbBr_4$	2-(2-aminoethyl)pyridine 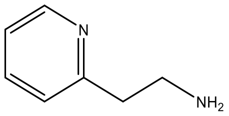	$[C_7H_{12}N_2]PbI_4$
3-(2-aminoethyl)pyridine 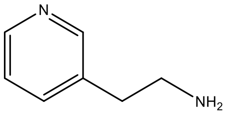	$[C_7H_{12}N_2]PbI_4$	4-(2-aminoethyl)pyridine 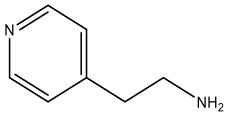	$[C_7H_{12}N_2]PbI_4$
N,*N*-Dimethyl-*p*-phenylenediamine 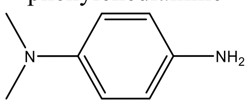	$[C_8H_{14}N_2]PbCl_4$	*N*,*N*-Dimethyl-*p*-phenylenediamine 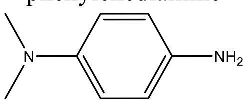	$[C_8H_{14}N_2]PbBr_4$
N,*N*-Dimethyl-*p*-phenylenediamine 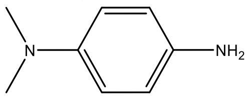	$[C_8H_{14}N_2]PbI_4$	*N*-(2-Aminoethyl)pyridine 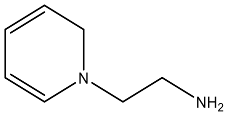	$[C_7H_{12}N_2]PbI_4$
Cystamine 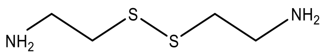	$[C_4H_{14}S_2N_2]PbBr_4$	1,2,4-Triazole 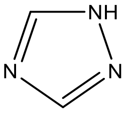	$[C_2H_4N_3]_2PbBr_4$

(2-Thiophene)ethylamine 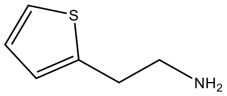	$[C_6H_{10}SN]_2PbI_4$	Phenethylamine 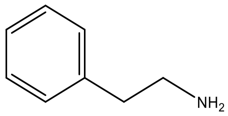	$[C_8H_{12}N]_2PbI_4$
Naphthalene-*O*-ethylamine 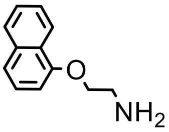	$[C_{12}H_{14}ON]_2PbI_4$	Pyrene-*O*-ethylamine 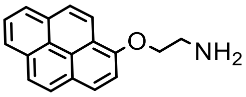	$[C_{18}H_{16}ON]_2PbI_4$
N-Methylaniline 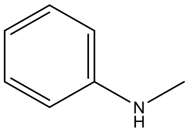	$[C_7H_{10}N]_2PbI_4$	2-(2-Naphthyl)ethylamine 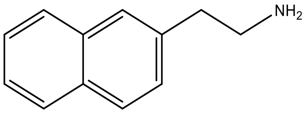	$[C_{12}H_{14}N]_2PbI_4$
Guanidine 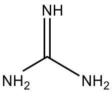	$[CH_6N_3]CsPbI_4$	Cyclohexylamine 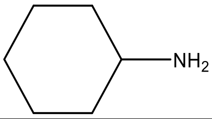	$[C_6H_{14}N]_2PbCl_4$
N-(2-Aminoethyl)piperidine 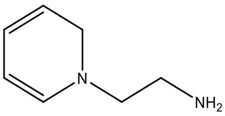	$[C_7H_{18}N_2]PbI_4$		

Table S4 Structures of amines corresponding to those in Table 4 of the main text.

Amine	Formula	Amine	Formula
2,2'-(Ethylenedioxy)bis(ethylamine) 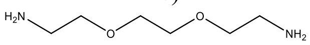	$[C_6H_{18}O_2N_2]PbCl_4$	3,5-Dihydroimidazo[4,5-f]benzimidazole 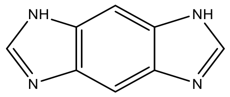	$[C_8H_8N_4]PbI_4$
1-(2-Naphthyl)-methylamine 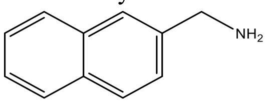	$[C_{11}H_{12}N]_2PbCl_4$	2-Iodoethylamine 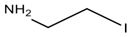	$[C_2H_7IN]_2PbI_4$
2-Hydroxyethylamine 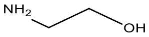	$[C_2H_8ON]_2PbI_4$	2,2'-Disulfanediyldiethanamine 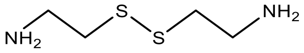	$[C_4H_{14}S_2N_2]PbI_4$
Pentylamine 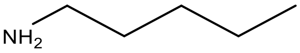	$[C_5H_{14}N]_2PbI_4$	Hexylamine 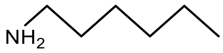	$[C_6H_{16}N]_2PbI_4$
Heptylamine 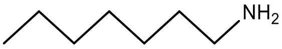	$[C_7H_{18}N]_2PbI_4$	4-Methylbenzylamine 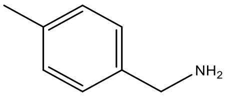	$[C_8H_{12}N]_2PbCl_4$
4-Methylbenzylamine 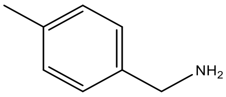	$[C_8H_{12}N]_2PbBr_4$	4-Methylbenzylamine 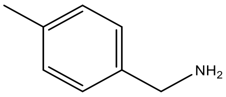	$[C_8H_{12}N]_2PbI_4$
(RS)-1-Phenylethylamine 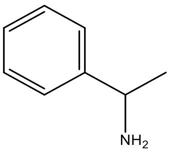	$[C_8H_{12}N]_2PbI_4$	4-Methoxy-*N*-methylaniline 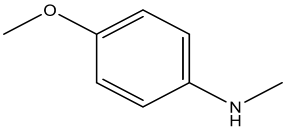	$[C_8H_{12}ON]_2PbI_4$
Octylamine 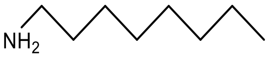	$[C_8H_{20}N]_2PbI_4$	Nonylamine 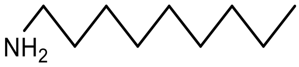	$[C_9H_{22}N]_2PbI_4$
Decylamine 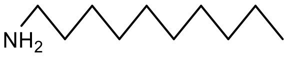	$[C_{10}H_{24}N]_2PbI_4$	Dodecylamine 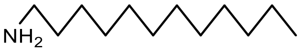	$[C_{12}H_{28}N]_2PbI_4$
Tetradecylamine 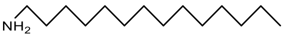	$[C_{14}H_{32}N]_2PbI_4$	Hexadecylamine 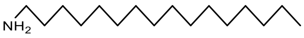	$[C_{16}H_{36}N]_2PbI_4$
Octadecylamine 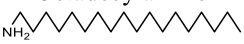	$[C_{18}H_{40}N]_2PbI_4$	2-Iodoethylamine 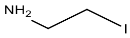	$[C_2H_7IN]_2PbI_4$
Ethylamine 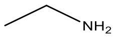	$[C_2H_8N]_2PbBr_4$	2-Hydroxyethylamine 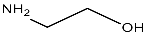	$[C_2H_8ON]_2PbI_4$
Cyclopropylamine 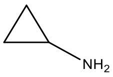	$[C_3H_8N]_2PbCl_4$	Cyclopropylamine 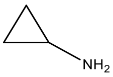	$[C_3H_8N]_2PbBr_4$
Cyclopropylamine 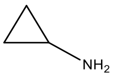	$[C_3H_8N]_2PbI_4$	3-Iodopropylamine 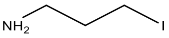	$[C_3H_9IN]_2PbI_4$

3-Hydroxypropylamine 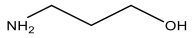	$[C_3H_{10}ON]_2PbI_4$	3-Butyn-1-amine 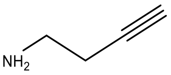	$[C_4H_8N]_2PbBr_4$
But-3-en-1-amine 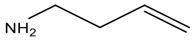	$[C_4H_8I_2N]_2PbBr_4$	Cyclobutylamine 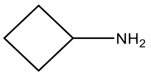	$[C_4H_{10}N]_2PbCl_4$
Cyclobutylamine 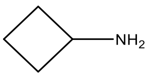	$[C_4H_{10}N]_2PbBr_4$	Cyclobutylamine 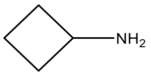	$[C_4H_{10}N]_2PbI_4$
4-Iodobutylamine 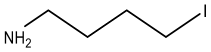	$[C_4H_{11}IN]_2PbI_4$	Isobutylamine 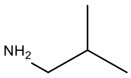	$[C_4H_{12}N]PbBr_4$
Isobutylamine 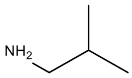	$[C_4H_{12}N]_2PbI_4$	3-Bromopyridine 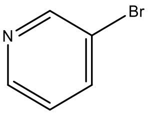	$[C_5H_5BrN]_2PbI_4$
Cyclopentylamine 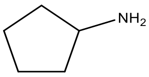	$[C_5H_{12}N]_2PbBr_4$	Cyclopentylamine 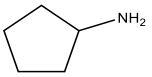	$[C_5H_{12}N]_2PbI_4$
5-Iodopentylamine 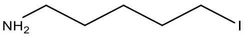	$[C_5H_{13}IN]_2PbI_4$	2-Aminopentane 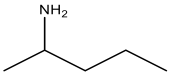	$[C_5H_{14}N]_2PbI_4$
3-Methylbutyl-1-amine 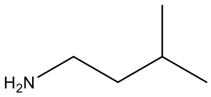	$[C_5H_{14}N]_2PbI_4$	4-Chloroaniline 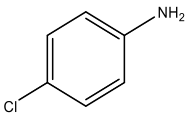	$[C_6H_7ClN]_2PbI_4$
4-Bromoaniline 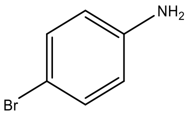	$[C_6H_7BrN]_2PbI_4$	6-Aminohexanoic acid 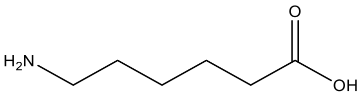	$[C_6H_{14}O_2N]$-$_2PbBr_4$
1,6-Diaminohexane 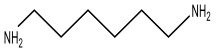	$[C_6H_{18}N_2]PbBr_4$	1,6-Diaminohexane 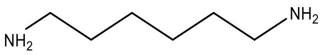	$[C_6H_{18}N_2]PbI_4$
3-Fluorobenzylamine 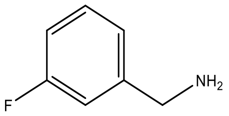	$[C_7H_9FN]_2PbCl_4$	4-Iodobenzylamine 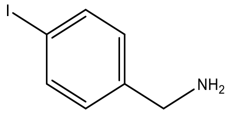	$[C_7H_9IN]_2PbI_4$
(Cyclohexylmethyl)amine 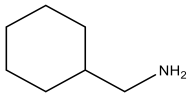	$[C_7H_{16}N]_2PbBr_4$	2-Aminoheptane 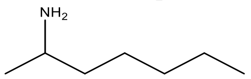	$[C_7H_{18}N]_2PbI_4$
2-(3,5-Dichlorophenyl)ethyl-1-amine 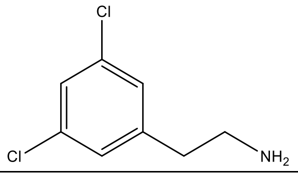	$[C_8H_{10}Cl_2N]_2PbI_4$	2-(3,5-Dichlorophenyl)ethyl-1-amine 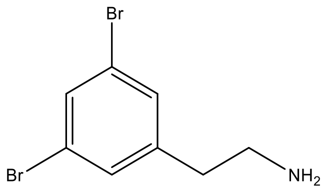	$[C_8H_{10}Br_2N]_2PbI_4$
4-Fluorophenethylamine 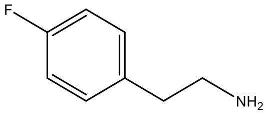	$[C_8H_{11}FN]_2PbI_4$	(RS)-1-(4-Chlorophenyl)ethylamine 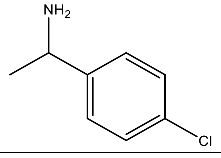	$[C_8H_{11}ClN]_2PbI_4$

2-Ethylhexylamine 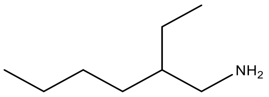	$[C_8H_{20}N]_2PbI_4$	1,4-Bis(aminomethyl)cyclohexane 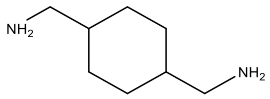	$[C_8H_{20}N_2]PbBr_4$
1,4-Bis(aminomethyl)cyclohexane 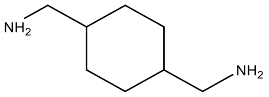	$[C_8H_{20}N_2]PbI_4$	1,8-Diaminooctane 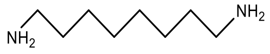	$[C_8H_{22}N_2]PbBr_4$
1,8-Diaminooctane 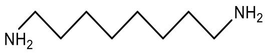	$[C_8H_{22}N_2]PbI_4$	1,5-Diaminonaphthalene 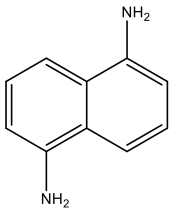	$[C_{10}H_{12}N_2]PbI_4$
2-(3,5-Dimethylphenyl)ethyl-1-amine 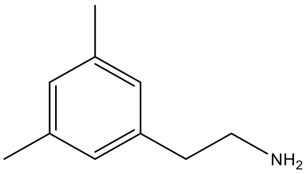	$[C_{10}H_{16}N]_2PbI_4$	1,10-Diaminodecane 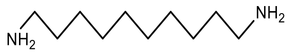	$[C_{10}H_{26}N_2]PbBr_4$
(RS)-1-(Naphthalen-1-yl)ethan-1-amine 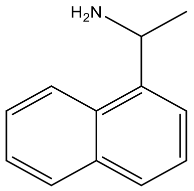	$[C_{12}H_{14}N]_2PbBr_4$	1,12-Diaminododecane 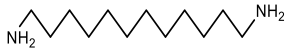	$[C_{12}H_{30}N_2]PbI_4$
Naphthalene-*O*-propylamine 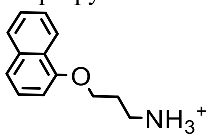	$[C_{13}H_{16}ON]_2PbI_4$	1-(4-Aminobutyl)pyrene 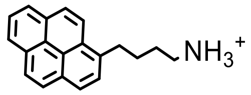	$[C_{20}H_{20}N]_2PbI_4$
Perylene-*O*-ethylamine 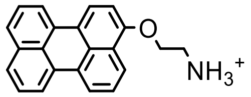	$[C_{22}H_{18}ON]_2PbI_4$	4-Chlorobenzylamine 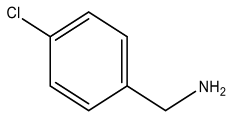	$[C_7H_9ClN]_2PbI_4$
4-Bromobenzylamine 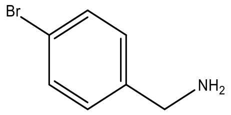	$[C_7H_9BrN]_2PbI_4$	2-Bromophenethylamine 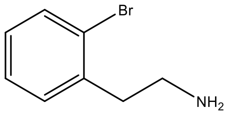	$[C_8H_{11}BrN]_2PbI_4$

(R)-1-Cyclohexylethylamine<br>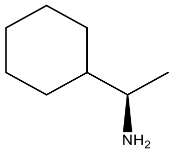	$[C_8H_{18}N]_2PbCl_4$	(S)-1-Cyclohexylethylamine<br>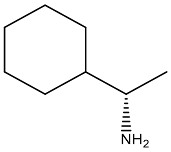	$[C_8H_{18}N]_2PbCl_4$
(R)-1-(Naphthalen-1-yl)ethyl-1-amine<br>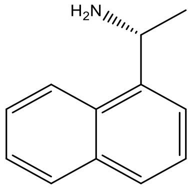	$[C_{12}H_{14}N]_2PbBr_4$	(S)-1-(Naphthalen-1-yl)ethyl-1-<br>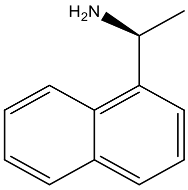<br>amine	$[C_{12}H_{14}N]_2PbBr_4$
4-4-Difluorohexahydroazepine<br>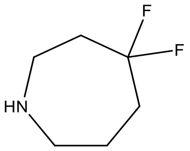	$[C_6H_{12}F_2N]_2PbI_4$	3,4-Diiodobut-3-en-1-amine<br>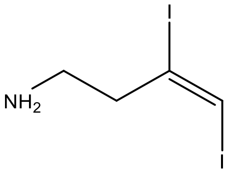	$[C_4\text{-}H_8I_2N]_2PbBr_4$
3,4-Dibromobutan-1-amine<br>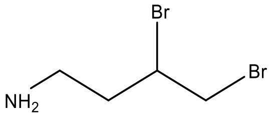	$[C_4H_{10}Br_2N]_2PbBr_4$	3,4-Dichlorobutan-1-amine<br>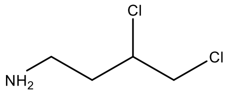	$[C_4H_{10}Cl_2N]_2PbBr_4$
1,4-Diaminobutane<br>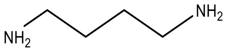	$[C_4H_{14}N_2]PbBr_4$	1,4-Diaminobutane<br>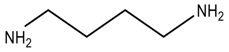	$[C_4H_{14}N_2]PbI_4$
5-Aminopentanoic acid<br>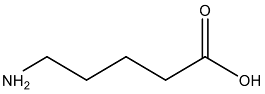	$[C_5H_{12}O_2N]_2PbCl_4$	5-Aminopentanoic acid<br>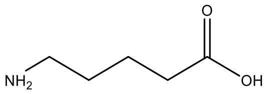	$[C_5H_{12}O_2N]_2PbBr_4$
2-Fluorophenethylamine<br>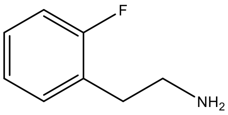	$[C_8H_{11}FN]_2PbI_4$	2-Phenethylamine<br>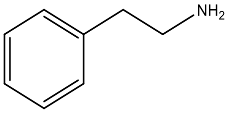	$[C_8H_{12}N]_2PbI_4$
5-ammoniumethylsulfanyl)-2,2'-bithiophene<br>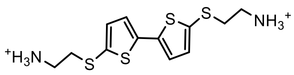	$[C_{12}H_{18}S_4N_2]PbI_4$	6-[(5-Methoxynaphthalen-1-yl)oxy]hexyl-1-amine<br>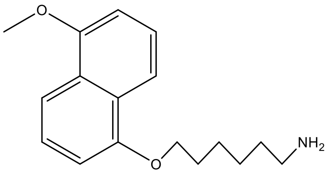	$[C_{17}H_{24}O_2N]_2PbI_4$
(R)-1-(4-Chlorophenyl)ethylamine<br>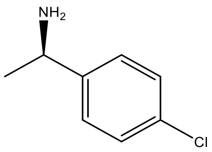	$[C_8H_{11}ClN]_2PbI_4$	S)-1-(4-Chlorophenyl)ethylamine<br>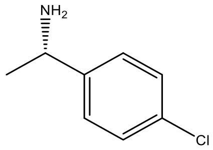	$[C_8H_{11}ClN]_2PbI_4$
2-(2-Naphthyl)ethylamine<br>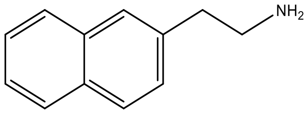	$[C_{12}H_{14}N]_2PbBr_4$	(2-Azaniumylethyl)trimethylphosphonium<br>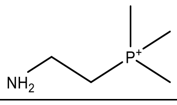	$[C_5H_{16}PN]PbBr_4$

2-(Aminomethyl)pyridine 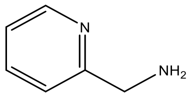	$[C_6H_{10}N_2]PbI_4$	4-(2-aminoethyl)imidazole 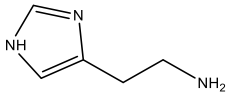	$[C_5H_{11}N_3]PbBr_4$
2-(2-aminoethyl)imidazole 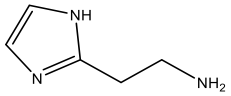	$[C_5H_{11}N_3]PbBr_4$	*N*^{1}, *N*^{1}-Dimethylpropane-1,3-diamine 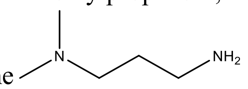	$[C_5H_{16}N_2]PbCl_4$
N^1, *N*^{1}-Dimethylpropane-1,3-diamine 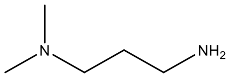	$[C_5H_{16}N_2]PbBr_4$	4-(Aminomethyl)piperidine 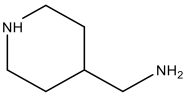	$[C_6H_{16}N_2]PbI_4$
Cyclohexane-1,2-diamine 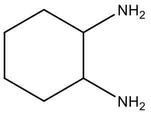	$[C_6H_{16}N_2]PbI_4$	2-Phenylethylamine 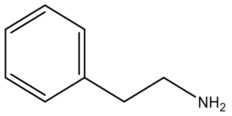	$[C_8H_{12}N]_2PbCl_4$
2-Phenylethylamine 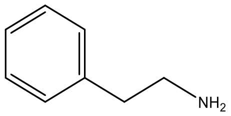	$[C_8H_{12}N]_2PbBr_4$	2-(1-Cyclohexenyl)ethylamine 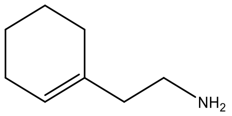	$[C_8H_{16}N]_2PbI_4$
2-(4-Methoxyphenyl)ethyl-1-amine 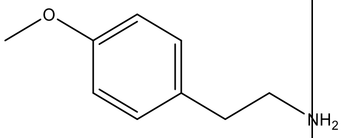	$[C_9H_{14}ON]_2PbI_4$	2-([2,2'-bithiophen]-5-yl)ethyl-1-amine 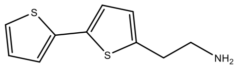	$[C_{10}H_{12}S_2N]_2PbI_4$
(Naphthalene-*O*-propylamine 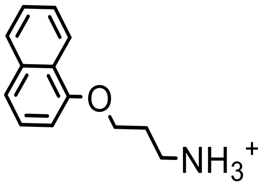	$[C_{13}H_{16}ON]_2[C_4H_6O_2]PbI_4$	2-(2^4,4^3-dimethyl[1^2,2^2:2^5,3^2:3^5,4^2-quaterthiophen]-1^5-yl)ethylamine 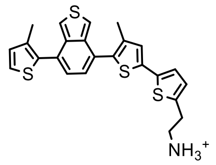	$[C_{24}H_{22}S_4N]_2PbI_4$
4-(Aminomethyl)piperidine 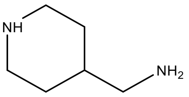	$[C_6H_{16}N_2]PbBr_4$	6-[(Naphthalen-1-yl)oxy]hexyl-1-amine 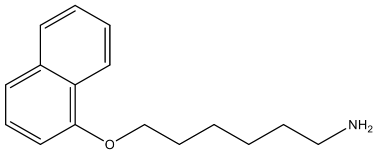	$[C_{16}H_{22}ON]_2PbI_4$

Table S5 Structures of amines corresponding to those in Table 5 of the main text.

Amine	Formula	Amine	Formula
N^1,N^1-dimethylpropane-1,3-diamine 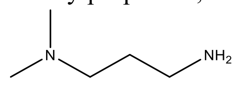	$[C_5H_{16}N_2]PbI_4$	4-Amidinopyridine 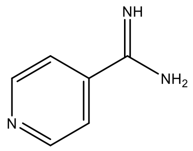	$[C_6H_9N_3]PbBr_4$
2-(Aminomethyl)pyridine 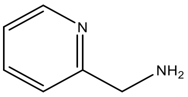	$[C_6H_{10}N_2]PbCl_4$	2-(Aminomethyl)pyridine 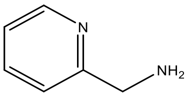	$[C_6H_{10}N_2]PbBr_4$
2-(Aminomethyl)pyridine 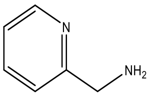	$[C_6H_{10}N_2]PbI_4$	1-(2-Aminoethyl)piperazine 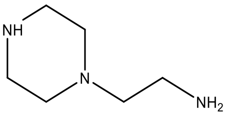	$[C_6H_{17}N_3]PbI_4$
3-(2-Aminoethyl)pyridine 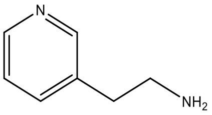	$[C_7H_{12}N_2]PbBr_4$	Cystamine 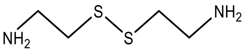	$[C_4H_{14}S_2N_2]PbI_4$
(RS)-2-Phenylpropyl-1-amine 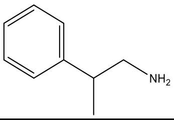	$[C_9H_{14}N]_2PbBr_4$	3-(Aminomethyl)piperidine 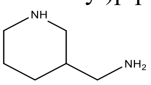	$[C_6H_{16}N_2]PbI_4$
Methylhydrazine 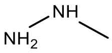	$[CH_7N_2]_2PbI_4$	Methylhydrazine 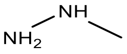	$[CH_7N_2]_2PbI_4$
N^1,N^1-dimethylethyl-1,2-diamine 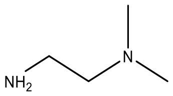	$[C_4H_{14}N_2]PbBr_4$	N^1,N^1-dimethylethyl-1,2-diamine 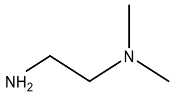	$[C_4H_{14}N_2]PbCl_4$
N^1,N^1-dimethylethyl-1,2-diamine 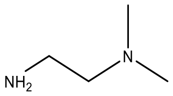	$[C_4H_{14}N_2]PbCl_4$	2-(Aminomethyl)pyridine 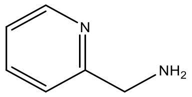	$[C_6H_{10}N_2]PbCl_4$
4-Aminobutanoic acid 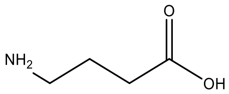	$[C_4H_{10}O_2N]_2PbBr_4$	N^1,N^1-dimethylbutyl-1,4-diamine 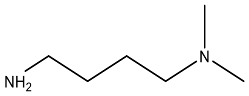	$[C_6H_{18}N_2]PbBr_4$
